# Fast non-line-of-sight imaging with high-resolution and wide field of view using synthetic wavelength holography

**DOI:** 10.1038/s41467-021-26776-w

**Published:** 2021-11-17

**Authors:** Florian Willomitzer, Prasanna V. Rangarajan, Fengqiang Li, Muralidhar M. Balaji, Marc P. Christensen, Oliver Cossairt

**Affiliations:** 1 Department of Electrical and Computer Engineering, Northwestern University, Evanston, IL 60208 USA; 2 Department of Electrical and Computer Engineering, Southern Methodist University, Dallas, TX 75205 USA; 3 Department of Computer Science, Northwestern University, Evanston, IL 60208 USA

**Keywords:** Imaging and sensing, Electrical and electronic engineering, Imaging techniques, Optical sensors

## Abstract

The presence of a scattering medium in the imaging path between an object and an observer is known to severely limit the visual acuity of the imaging system. We present an approach to circumvent the deleterious effects of scattering, by exploiting spectral correlations in scattered wavefronts. Our Synthetic Wavelength Holography (SWH) method is able to recover a holographic representation of hidden targets with sub-mm resolution over a nearly hemispheric angular field of view. The complete object field is recorded within 46 ms, by monitoring the scattered light return in a probe area smaller than 6 cm × 6 cm. This unique combination of attributes opens up a plethora of new Non-Line-of-Sight imaging applications ranging from medical imaging and forensics, to early-warning navigation systems and reconnaissance. Adapting the findings of this work to other wave phenomena will help unlock a wider gamut of applications beyond those envisioned in this paper.

## Abstract (web summary)

Here, the authors present Synthetic Wavelength Holography, an approach for Non-Line-of-Sight imaging. By exploiting spectral correlations in scattered light, the authors transform real world surfaces such as walls or scatterers into High-Resolution, Wide-Field-of-View imaging portals that provide holograms of objects obscured from view.

## Introduction

There are numerous instances of imaging within the physical sciences wherein an opaque barrier (such as a wall) or a scattering medium (such as fog or tissue) impedes direct view of the object. Over the years, many attempts^1–13^ have been made to non-invasively recover images of objects obscured from direct view. These techniques are collectively referred to as Non Line-of-Sight Imaging (NLoS) in our work. The problem is enjoying renewed attention due to potential applications in autonomous navigation, planetary exploration, industrial inspection, and early warning systems for first-responders^14–27^. Imaging through deep turbulence or fog, face identification around corners, or imaging through optically opaque barriers like skulls are just a few of the potential application scenarios.

Broadly speaking, current approaches to NLoS imaging circumvent the effect of scatter in one of two geometries: continuous scattering within a volume such as fog or tissue, and discrete scattering events distributed across multiple interfaces such as walls. A specific embodiment of the discrete scattering problem in NLoS imaging is the challenge of looking around corners. We use this task to motivate the proposed Synthetic Wavelength Holography (SWH) approach and provide a basis for the comparative assessment with competing approaches. We do not consider passive approaches^15,28–31^ and restrict our attention to active NLoS imaging schemes. Existing active schemes for imaging around corners attempt to recover the obscured scene by either exploiting the finite speed of light (time of flight (ToF) based techniques^16,20–22^) or spatial correlations in scattered light (memory effect (ME) based techniques^23,32–36^).

ToF based NLoS Imaging techniques recover a surface representation of the hidden scene by probing the scene with a temporally modulated source, and recording the response using fast detectors. The process is repeated across multiple spatial locations (so-called virtual sources and detectors - VS and VD) of an intermediary surface such as a wall or floor that is simultaneously visible to the obscured objects and the NLoS sensor unit. Recent work in the area^16,18–22^ has demonstrated results with cm-scale lateral resolution over a 1 m × 1 m × 1 m working volume, and in select cases providing near real-time reconstructions. In many cases the approach, however, is limited by the need for raster-scanning large areas on the intermediary VS/VD surface whose dimensions are comparable to the obscured volume.

The second class of techniques for imaging around corners exploits spatial or angular correlations in scattered light^23,32–37^. The images of obscured objects recovered using these techniques feature the highest lateral resolution (<100 μm at 1 m standoff), for the smallest probing area on the intermediary VS/VD surface (<few cm). The improved resolution, however, comes at the expense of a highly restricted angular FoV (<2^∘^), as determined by the angular decorrelation of scattered light (memory effect ME^38,39^). The ME does not only limit the FoV, but also the maximal possible size of the measured object which cannot exceed the respective working volume.

The wide disparity in the FoV and resolution of current NLoS Imaging schemes greatly limits their utility. We address the challenge by exploiting spectral correlations in scattered light to recover a high-resolution holographic representation of the obscured scene, over a wide angular field of regard, using only two measurements. The approach dubbed synthetic wavelength holography (SWH) represents an advancement of the state-of-the-art in NLoS imaging. SWH makes specific use of the observation that coherent light at two closely spaced wavelengths *λ*_1_, *λ*_2_ traversing near identical geometric paths in a scattering medium, preserves phase information at scales exceeding a synthetic wavelength (SWL) Λ > > *λ*_1_, *λ*_2_^40,41^. We establish that the optimal choice of the SWL scales with increasing scatter, and the synthetic phase computed at the distal end of the scattering medium encodes a holographic representation of the obscured objects. The mathematical principles underlying the proposed imager concept expand the understanding of light transport in any scattering geometry. However, the SWH idea is by no means restricted to the applications discussed above. The idea of utilizing wavelength diversity to alleviate the effects of unwanted aberrations in the detection of electromagnetic signals has potential applications that go far beyond the original scope of NLoS imaging. We conclude this paper by discussing benefits of applying the SWH principle in a diverse set of application areas.

## Results

### Synthetic wavelength holography

Our SWH approach draws inspiration from multi-wavelength interferometry on rough surfaces^40–46^: We exploit spectral correlations in scattered light at optical wavelengths *λ*_1_, *λ*_2_ to assemble a hologram of the obscured objects at the SWL ${{\Lambda }}=\frac{{\lambda }_{1}{\lambda }_{2}}{| {\lambda }_{1}\,-\,{\lambda }_{2}| }$. The approach provides a combination of capabilities that is unmatched by competing NLoS approaches:

- **Small probing area:** the majority of ToF based NLoS schemes use probing areas ~1m × 1m. SWH provides the ability to image obscured objects in tightly confined spaces by simultaneously illuminating and observing a small area (58 mm × 58 mm in our experiments).
- **Wide angular field of view:** ME-based approaches produce highly restricted FOVs (<2^∘^ for drywall), while SWH as a holographic method provides the ability to recover obscured objects over a nearly hemispherical FoV that far exceeds the limited angular extent of the memory effect (see Supplementary Sec. 5).
- **High spatial resolution:** ToF-based approaches generally produce low spatial resolutions (~cm). SWH provides the ability to resolve small features on obscured objects (up to <1 mm in our experiments) without requiring prior knowledge of the scattering geometry or attributes of the scattering medium such as the transmission matrix^47,48^.
- **High temporal resolution:** ToF-based approaches require point-wise raster-scanning, while we demonstrate the ability to recover holograms of the obscured object within two shots using conventional focal plane array (FPA) technology. The SWH principle even allows for single-shot acquisition (see Methods section).

Holographic approaches to imaging, including SWH, exploit the availability of a coherent source for illumination and interferometric sensing, which is used to capture the complex-valued representation of the optical field $E({\lambda }_{1})\,=\,{A}_{1}\cdot {e}^{i\phi ({\lambda }_{1})}$ with amplitude *A*_1_ and phase *ϕ*(*λ*_1_) at a given wavelength *λ*_1_. Repeated scattering irreversibly randomizes the phase *ϕ*(*λ*_1_) so that the speckle pattern recorded on the detector contains no information about the macroscopic structure of the object.

**Fig. 1:**
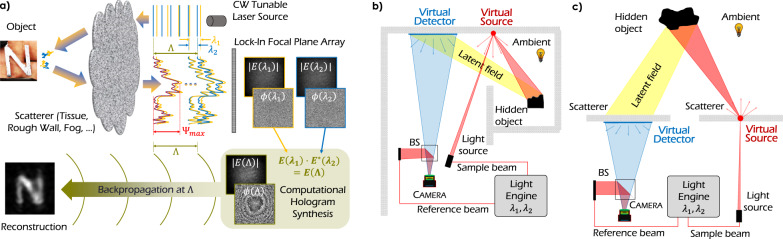
Schematics of SWH for NLoS imaging around corners and NLoS imaging through scattering media. **a** SWH image formation: A continuous wave (CW) tunable laser illuminates the scene at two slightly different wavelength *λ*_1_ and *λ*_2_. Each field *E*(*λ*_1_), *E*(*λ*_2_) is subject to multiple scattering processes in or at the scatterer (which could be wall, tissue, fog,...) and the rough object surface. The introduced maximal pathlength variation Ψ_*max*_ leads to a complete randomization of *E*(*λ*_1_), *E*(*λ*_2_) (with respective phasemaps *ϕ*(*λ*_1_), *ϕ*(*λ*_2_)) when arriving at the detector. However, computational mixing of the speckled fields *E*(*λ*_1_) ⋅ E^*^(*λ*_2_) = *E*(Λ), yields a complex-valued hologram of the object at a Synthetic Wavelength (SWL) ${{\Lambda }}=\frac{{\lambda }_{1}\cdot {\lambda }_{2}}{| {\lambda }_{1}-{\lambda }_{2}| }$. The object is reconstructed by backpropagating *E*(Λ) with the SWL Λ. **b** and **c** Schematic setups for NLoS imaging around corners (**b**) and NLoS imaging through scatterers (**c**) with the SWH principle: The sample beam illuminates a spot on the wall/scatterer (the Virtual Source VS), which scatters light towards the obscured object. A small fraction of the light incident on the object is scattered back to the wall/scatterer where it hits the Virtual Detector (VD). The VD is imaged by the camera, meaning that the synthetic hologram is captured at the VD surface. Details and internals of the light engine are specified in the Supplementary Fig. 4.

However, a small change in the interrogation wavelength produces another speckle pattern *E*(*λ*_2_). If the illumination beams at the two wavelengths originate from the same source position (such as from a single fiber) and the inhomogeneities in the scattering medium are quasi-static, the fields *E*(*λ*_1_), *E*(*λ*_2_) incident on the detector are highly correlated. This is because the light at the two wavelengths traverses nearly identical ray paths and experiences nearly identical path length fluctuations. Hence, the residual changes in phase ∣*ϕ*(*λ*_1_) − *ϕ*(*λ*_2_)∣ encode the macroscopic structure of the obscured object^34,40–44^. We exploit this fact to probe the scattering medium at two closely spaced wavelengths *λ*_1_ and *λ*_2_, and record the emerging speckle fields *E*(*λ*_1_) and *E*(*λ*_2_), as illustrated in Fig. 1a. Computational mixing of the fields *E*(*λ*_1_) ⋅ E^*^(*λ*_2_) = *E*(Λ), yields a complex-valued hologram of the object at the SWL ${{\Lambda }}=\frac{{\lambda }_{1}\cdot {\lambda }_{2}}{| {\lambda }_{1}\,-\,{\lambda }_{2}| }$ (see Fig. 1a).

1 $$E({{\Lambda }})=	 \, E({\lambda }_{1})\cdot {E}^{* }({\lambda }_{2})\\ =	 \, {A}_{1}{A}_{2}\cdot {e}^{i(\phi ({\lambda }_{1})-\phi ({\lambda }_{2}))}={A}_{1}{A}_{2}\cdot {e}^{i\phi ({{\Lambda }})}$$

For closely spaced optical wavelength *λ*_1_ and *λ*_2_, the SWL is orders of magnitude larger than *λ*_1_, *λ*_2_. Although the magnitude of the synthetic field given by ∣*E*(Λ)∣ = ∣*E*(*λ*_1_)∣ ⋅ ∣*E*(*λ*_2_)∣, still exhibits speckle artifacts (see e.g. Fig. 1a), the synthetic phase *∠E*(Λ) = *ϕ*(Λ) = *ϕ*(*λ*_1_) − *ϕ*(*λ*_2_) is robust to the deleterious effects of scattering and an image of the hidden object can be retrieved by numerical backpropagation of *E*(Λ) at the SWL Λ, as illustrated in Fig. 1a.

The complex-valued speckle fields *E*(*λ*_1_) and *E*(*λ*_2_) can be recorded in many different ways. Common procedures include phase-shifting of the reference beam with respect to the object beam, or spatial heterodyning^49,50^. In this work, we exploit *frequency heterodyning* paired with a focal plane array (FPA) lock-in sensor^51^ to retrieve the complex-valued synthetic field *E*(Λ). The technical details of heterodyne detection with a lock-in camera are described in the Methods section. The resulting ‘dual wavelength heterodyne’ or ‘superheterodyne’ interferometers have particularly advantageous properties under low light conditions, since they exploit the heterodyne gain^52^ afforded by a strong reference beam. This makes them a perfect fit for the NLoS problem, which frequently suffers from low light return due to multiple scattering. The SWH-based NLoS imaging strategy described above is strikingly simple and computationally inexpensive. The computational immunity to scatter afforded by the existence of spectral correlations, relies only on the wave nature of light. As a consequence, the principles underlying SWH can be readily extended to other wave phenomena such as radio waves and acoustic waves (ultrasound).

In the following we will experimentally validate our SWH principle for different NLoS imaging techniques

### Looking around corners

We use the scene arrangement depicted in Fig. 1b to elucidate the SWH principle and demonstrate the ability to record holograms of objects beyond the line-of-sight. The portions of the wall designated VS and VD are used to indirectly illuminate the hidden object, intercept the scattered latent field and relay it towards a focal plane array (FPA) that records the scattered field. Details of the image acquisition process and the imaging apparatus are provided in the Methods section and the Supplementary Material. An image of the obscured object is recovered as explained above, i.e., by mixing the scattered optical fields *E*(*λ*_1_) and *E*(*λ*_2_) and backpropagating the assembled synthetic wavelength hologram *E*(Λ) with the SWL Λ. The use of tunable lasers allows us to accommodate a variety of scattering scales by freely tuning the SWL over a wide interval ranging from hundreds of μm to hundreds of *m*.

**Fig. 2:**
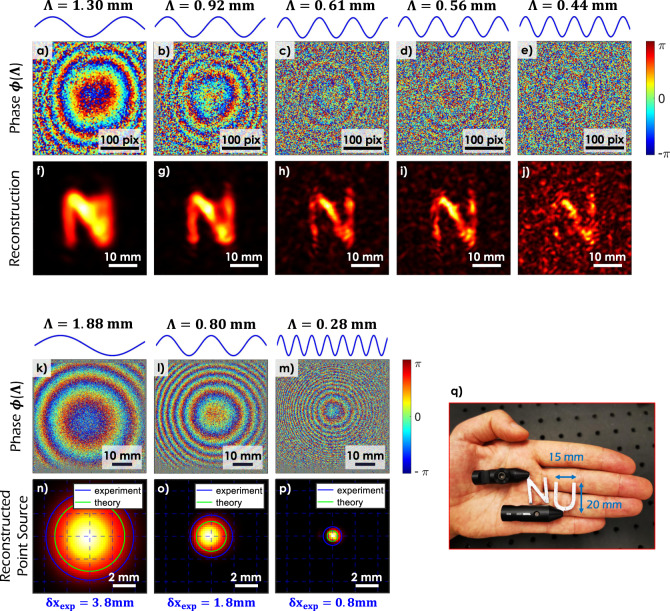
Experimental demonstration of looking around corners using SWH and an assessment of its resolving power. **a**–**j** Imaging the character ‘N’ (~15 mm × 20 mm) around the corner at five different SWLs. A schematic of the experimental setup is available in Fig. 1b. **a**–**e** The phase of the synthetic holograms at the VD surface. **f**–**j** Respective reconstructions (squared magnitude of the backpropagated synthetic fields at the standoff distance of the character). The resolution of the reconstructions increases with decreasing SWL. However, the speckle-artifacts increase due to the decorrelation of the two optical fields at *λ*_1_ and *λ*_2_. **k**–**p** Synthetic diffraction disc: reconstruction of an obscured point source for three different SWLs. **k**–**m** Phase of the synthetic holograms at the VD surface. **n**–**p** Reconstruction of the ‘synthetic diffraction disc’. As in classical optics, the disc size varies linearly with the wavelength (in this case the SWL). The experimental value is close to the theoretical expectation. For **p**, the point source is reconstructed with sub-mm precision. **q** Image of the targets used in the experiments of this paper: Two characters ‘N’ and ‘U’ with dimensions ~ 15 mm × 20 mm (plus black mountings).

Figure 2a–e illustrates the phase of the computationally assembled synthetic wavelength hologram, for a specific set of SWLs. In each case, we are able to recover optical phase information, despite the pronounced scattering at the wall and object surface. The outcome of backpropagating the synthetic holograms is shown in Fig. 2f–j. The reconstructions show the squared magnitude of the backpropagated synthetic fields at the standoff distance of the character. As expected, this is the bakpropagation distance that produces the sharpest image of the character for the respective SWL. The results confirm the ability to recover an image of a small object, character ‘N’ (dimensions 15 mm × 20 mm, see Fig. 2q) that is obscured from view. Furthermore, the *phase information* encapsulated in the synthetic hologram allows us to locate the hidden object within the obscured volume (illustrated in Fig. 4 and described later).

Figure 2f–j confirms that the resolution of the reconstruction improves with decreasing SWL Λ. This behavior is in agreement with results from classical holography. It implies that the synthetic wave, although a computational construct, has distinct characteristics that it shares with a physical wave at the respective wavelength Λ. The notion is corroborated in Fig. 2k–p wherein we attempt to localize a point-source obscured from view. It is observed that a diffraction limit may be ascribed to the computationally assembled synthetic hologram, in much the same manner as classical holography. The radius of the resulting synthetic diffraction disc is given by:

2 $$\delta x\approx {{\Lambda }}\frac{z}{D},$$

where *D* is the diameter of the probing area at the VD, and *z* is the propagation distance to the object. The ratio *D*/2*z* defines the numerical aperture of the computational NLoS-imager. In the present example, a lateral resolution limit of *δx* = 800 μm is reached for a SWL of Λ = 280 μm. Details of the experiment are included in the Methods section.

The expression for the resolving power of SWH disclosed in Eq. (2), suggests that the resolution may be indefinitely improved by reducing the SWL Λ. This however is not the case. For increasingly small values of Λ, the reconstructions exhibit speckle-like artifacts (see e.g. Fig. 2f–j). The artifacts are attributed to a loss in the spectral correlation of the scattered fields observed at *λ*_1_, *λ*_2_. The physical origins of this decorrelation are well documented in literature^39,42–44,53–61^. The Supplementary Material includes a derivation for the specific case of looking around corners.

### Looking through scatter

**Fig. 3:**
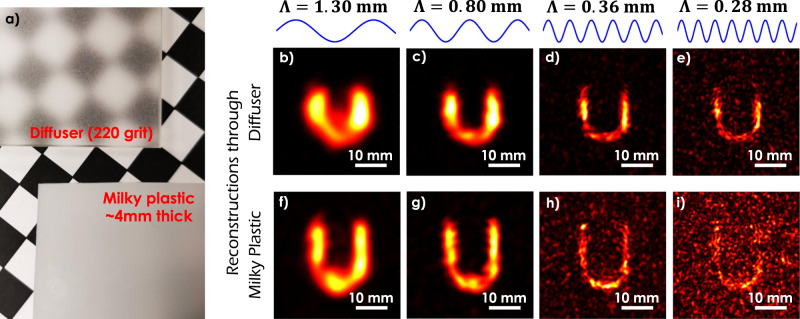
Experimental demonstration of imaging through scattering media using SWH. A schematic of the experimental setup is available in Fig. 1c. **a** Scatterers obscuring the object: A 220-grit ground glass diffuser and a milky white plastic plate of ~4 mm thickness, both placed ~1 cm over a checker pattern to demonstrate the degradation in visibility. **b**–**e** Reconstructions of measurements taken through the ground glass diffuser for different SWLs Λ. **f**–**i** Reconstructions of measurements taken through the milky plastic plate for different SWLs Λ. The character can be reconstructed with impressive quality. The larger OPD in the plastic plate leads to greater decorrelation if the SWL is decreased.

The notion of exploiting spectral correlations for NLoS imaging is by no means restricted to the looking around corners problem. To highlight the versatility of the SWH approach, we recover holograms of objects embedded beneath a scattering medium, as illustrated in the schematic of Fig. 1c. In a first set of measurements, we image the small character ‘U’ (dimensions 15 mm × 20 mm, see Fig. 2q) through a 220-grit diffuser (Fig. 3a top). Since scattering occurs only at one surface of the diffuser, this experiment can be considered as the transmissive equivalent of the looking around corners experiment described above. The holographic reconstructions are displayed in Fig. 3b–e. As the SWL falls below 300 μm, we begin to notice ‘synthetic speckle’ artifacts in the reconstructed image, suggesting that wavelength separation has increased to the point that the captured optical holograms are uncorrelated for this specific scene.

In a second set of measurements, we swap the diffuser in the imaging path with a 4 mm thick milky white plastic plate. Figure 3a illustrates the impact of pronounced multiple scattering on the visibility of a checkerboard that is viewed through the plastic plate and the 220-grit ground glass diffuser. It is clear from the results of Fig. 3f–i that we are able to reconstruct the character ‘U’ for SWLs Λ ≥ 360 μm. The results confirm the ability to recover image information at visibility levels that are far below the perceptual threshold, due in large part to scattering.

A comparison of the reconstruction results for the plastic plate and the diffuser reveals only a marginal change in the smallest achievable SWL, as we switch from thin scattering surfaces to thick scattering media. The implications of this observation are best understood by recognizing that the visibility of ballistic light paths decays exponentially with the propagation distance through a scattering volume (in accordance with Beer’s law^62^). To quantify the severity of scattering in the 220-grit diffuser and the plastic plate, we performed additional experiments that examine the decay rate of spectral correlations in the speckle patterns emerging from the scattering medium as the frequency of the laser emission is swept. The experiments are detailed in the Supplementary Sec. 6 and directly evaluate the smallest achievable SWL that delivers completely speckle-artifact free reconstructions. It is observed that the smallest SWL for the plastic plate is Λ = 400 μm, and Λ = 300 μm for the 220-grit diffuser. This is in accordance with our with our experimental results which begin to exhibit speckle artifacts in Fig. 3e, h.

### Synthetic pulse holography

The principles underlying the proposed SWH concept can be extended to include multiple illumination wavelengths. By recording fields at many regularly spaced wavelengths, we can computationally engineer a pulsed light source, much like those found in transient imaging, but using continuous wave measurements. The difference to transient ToF approaches is that our detection procedure allows for much better localization of this pulse, leading to an improvement in the longitudinal resolution, in much the same manner as optical coherence tomography (OCT)^63–66^ and white-light interferometry (WLI)^67,68^. However, unlike OCT and WLI, we do not need to match the power and pathlengths in the two inteferometer arms.

**Fig. 4:**
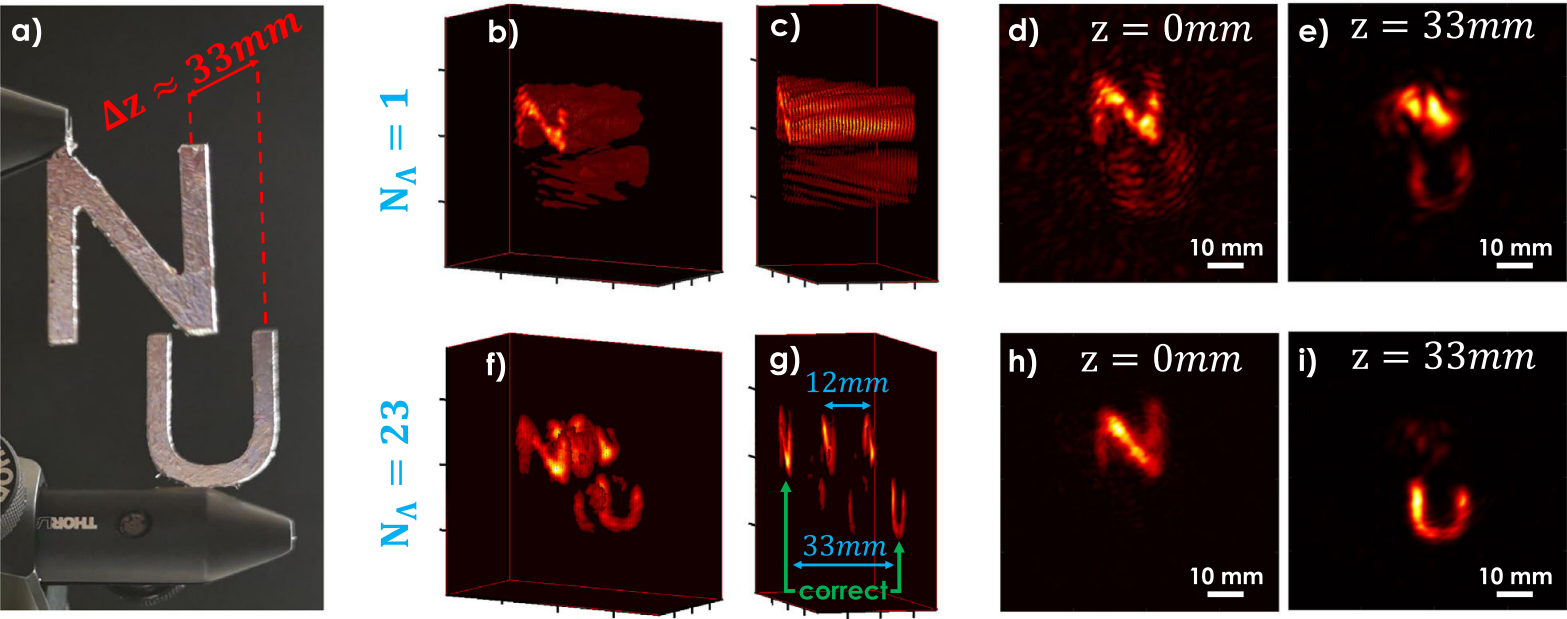
Experimental demonstration of synthetic pulse holography: generation of a ’synthetic pulse train’ using SWH. **a** Target, consisting of two characters with a longitudinal separation of 33 mm. **b**–**e** Reconstruction of the characters, using only *N*_Λ_ = 1 SWL (Λ = 0.8 mm). Due to the properties of holographic backpropagation, a separation of the characters for different depths *z* is not possible. **f**–**i** Reconstructions obtained from the coherent superposition of the backpropagated fields at *N*_Λ_ = 23 SWLs. Letters are separable. The pulse distance of the synthesized pulse train can be seen in **f** and **g**.

To demonstrate the improvement in longitudinal resolution afforded by the use of multiple SWLs, we computationally section a three-dimensional NLoS scene comprised of the two previously introduced characters ‘N’ and ‘U’ (both 15 mm × 20 mm) that are offset in depth by Δ*z* ≈ 33 mm. Using a single SWL of Λ = 800 μm it is possible to separate the characters laterally, but with limited longitudinal resolution, as shown in Fig. 4b–e. Since we have access to the complex-valued field information at each synthetic wavelength, it is possible to improve the longitudinal resolution of SWH by coherently combining the synthetic fields recorded at a multitude of synthetic wavelengths (*N*_Λ_ = 23 in Fig. 4). The computational approach mimics scene interrogation by a periodic pulse train, and the replicas observed in the reconstructions of Fig. 4f, g are consistent with the periodicity of the computationally engineered pulse train (frequency offset of 25 GHz corresponding to a depth ambiguity of 12 mm). An unambiguous measurement range in excess of 33 mm requires a frequency increment of ~1 GHz, which has been experimentally verified with our laser system as well. It is anticipated that locking the tunable laser source to a frequency ruler such as a frequency comb will further improve the longitudinal resolution, due in large part to the precise phase relationship between the individual comb teeth^69–71^.

### Wavefront sensing through scatter

**Fig. 5:**
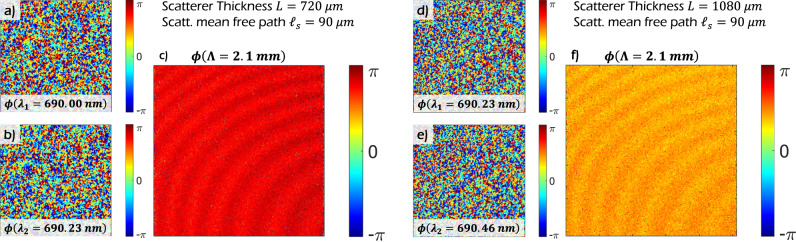
Experimental demonstration of wavefront sensing through scatter using SWH. The data for this experiment where captured by the authors of Kadobianskyi et al.^72^ without the intention to be used for our approach. Nevertheless, our SWH reconstruction mechanism is able to recover residual phase variations in speckled wavefronts emerging from volumetric scattering samples. **a** and **b** Scattered (speckled) phasemaps *ϕ* after the volumetric scattering sample with thickness *L* = 720 μm and scattering mean free path *ℓ*_*s*_ = 90 μm for two different wavelengths *λ*_1_ = 690.00 nm and *λ*_1_ = 690.23 nm. **c** Calculated synthetic phase map for Λ = 2.1 mm. **d**–**f** Same experiment with scatterer of different thickness (*L* = 1080 μm) and optical wavelengths *λ*_1_ = 690.23 nm and *λ*_1_ = 690.46 nm.

The experiments in SWH described thus far have restricted attention to recovering objects obscured by scattering media. However, the principle underlying SWH, namely spectral correlations in scattered light, is rather general and has broader appeal. In particular, the availability of phase information at the optical wavelength of interrogation makes it possible to recover the phasefront of light incident on the other side of a scattering material. Possible applications include, e.g., wavefront shaping through scattering media. We demonstrate the ability to recover residual phase variations in the wavefronts emerging from a volumetric scattering sample. The data for this experiment where captured by the authors of Kadobianskyi et al.^72^ without the original intention to be used for our approach. Details of the experimental apparatus are available in Kadobianskyi et al.^72^. The authors recorded speckle fields emerging from 720 μm (Fig. 5a, b) and 1080 μm (Fig. 5d, e) thick scattering samples with a scattering mean free path of 90 μm. In each case, the sample is interrogated by a quasi-monochromatic collimated beam at 801 equally spaced wavelength steps spanning the range 690 nm to 940 nm. By computationally mixing speckle holograms recorded at adjacent wavelengths, we are able to identify a synthetic hologram at the SWL of Λ = 2.1 mm. Results from the experiment are shown in Fig. 5. The phase of the synthetic hologram exhibits a distinct spatial structure that is consistent with the observation of interference fringes due to inter-reflections between the laser aperture and a polarized beam splitter in the illumination path; according to the authors of Kadobianskyi et al.^72^. It is worth emphasizing that the wavefront sensing approach described above, relies only on the scattered light paths as the ballistic paths are expected to be extinguished by factors of 10^−8^ and 10^−12^ in the 720 μm and 1080 μm sample respectively.

## Discussion

The present work combines the expressive power of holography with spectral correlations in scattered light to address the challenging problem of NLoS imaging. Using only a small probing area (58 mm × 58 mm in our experiments), we are able to recover a high-resolution (up to <1 mm) holographic representation of obscured objects, over a short time interval within a nearly hemispherical angular FoV. The technique is robust enough to accommodate different NLoS imaging scales and scattering geometries.

The use of continuous wave sources at different wavelengths allowed us to bypass the need for ultrafast sources (picosecond pulses) and fast detectors (SPAD detectors, streak cameras), both of which are routinely employed in competing NLoS approaches. The use of full-field focal plane arrays additionally allowed us to bypass the need for raster scanning.

**Fig. 6:**
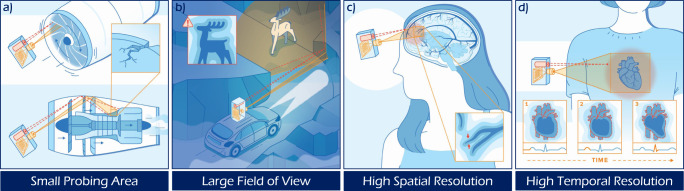
Key attributes and potential future applications of SWH. The SWH approach combines four key attributes highlighted in the following potential future NLoS application scenarios: in each example, a scattering surface or medium is used to indirectly illuminate, and intercept light scattered by the hidden objects. **a** A small probing area allows to inspect defects in tightly confined spaces, e.g., in running aircraft engines. **b** A wide angular FoV allows to measure/detect hidden objects without previous knowledge of their position as, e.g., important when navigating in degraded visual environments. **c** High spatial resolution allows for the measurement of small structures, such as non-invasive imaging of brain vessels through the skull. **d** High temporal resolution allows to image objects in motion, e.g., to discern cardiac arrhythmia through the chest. The integration of these attributes in a single approach to NLoS imaging distinguishes SWH from the current state of the art.

We have demonstrated that SWH combines a set of important imaging attributes including: a wide angular field of view at a small probing area, as well as high spatial and temporal resolution. Each of these attributes has the potential to enable new and exciting NLoS imaging applications in the future with examples envisioned in Fig. 6: A small probing area potentially allows for inline defect detection and inspection in heavy machinery such as running turbines, as well as endoscopic and keyhole^17^ imaging applications (Fig. 6a). A wide angular field of view is needed for the design of early warning systems in automotive sensing (Fig. 6b). High spatial resolution could potentially enable the non-invasive imaging of blood vessels through the skull (Fig. 6c). High temporal resolution could help in resolving motion of obscured objects such as sensing cardiac arrhythmias through the chest. Although the experimental results shown in this work merely represent the first step towards demonstrating each of the aforementioned potential applications, the combination of all four important attributes in one approach (as provided by SWH) is unmatched by competing NLoS approaches. It should be emphasized however, that competing approaches share different subsets of those attributes. For example, one experiment shown in^73^ impressively combines the first three attributes, however, at the cost of a very long acquisition time. Another advantage of the proposed SWH approach emerges in enhanced capabilities such as wavefront sensing through scatter, which transcend conventional NLoS imaging.

Despite the advantages of SWH, the method is not without limitations, chief of which is the inability to recover phase information when the optical fields at the two wavelengths *E*(*λ*_1_) and *E*(*λ*_2_) are uncorrelated. The effect is analogous to the memory effect for angular decorrelation and hence can be interpreted as a spectral memory effect. SWL measurements outside this spectral ME lead to speckle-like artifacts in the reconstructed images (‘synthetic speckle’), such as those observed in Figs. 2j and 3i. The problem may be avoided by judicious selection of the interrogation wavelengths *λ*_1_, *λ*_2_. It is observed that phase fluctuations exceeding the SWL may be unambiguously recovered if the largest wavefront error Ψ_max_ introduced during light transport through the scattering scene fulfills a Rayleigh Quarter Wavelength criterion (RQWR^74^) for the SWL, i.e.,

3 $${{{\Psi }}}_{{{{{{\mathrm{max}}}}}}}\le \frac{{{\Lambda }}}{4}$$

The wavefront error Ψ_max_ represents the worst-case spread in the physical lengths of scattered light paths that share a common source location, object location, and detector pixel. For surface scattering processes, the spread in path lengths is limited by 2*σ*_*h*_, where *σ*_*h*_ represents the RMS surface roughness (see Supplementary Eq. 40).

The Supplementary Material puts forth mathematical arguments supporting the existence of RQWR (Eq. (3)) for a single realization of a scattering surface (see Supplementary Sec. 1.6). The analysis shows how the RQWR fundamentally limits performance of a large class of NLoS imagers, including ToF techniques. Furthermore, the analysis may be generalized to include volumetric scatter by adopting a diffusive approach to light propagation^60^. It is observed that the spread in path lengths as determined by the ratio of the squared thickness of the medium *L*^2^ to the transport mean free path ℓ^*^, plays a role analogous to the RMS roughness *σ*_*h*_ of scattering surfaces.

The principal distinction between scattering at discrete interfaces such as walls and continuous scattering through a volume, lies in the scale of wavefront error Ψ_max_. For instance, the typical wavefront error Ψ_max_ for imaging around corners is less than 1 millimeter, several centimeters for imaging through tissue, and many meters for long-range imaging through fog. It is expected that diffusive scatter over long propagation distances will severely limit the maximal achievable resolution. In the specific case of imaging through fog, we anticipate that the time-gating ability of FMCW LiDAR^75^ may be combined with SWH to see farther with a higher resolution than is otherwise possible. In the case of imaging through tissue, it is anticipated that ultrasound focusing aids may be combined with SWH to see farther into the brain with a higher resolution than is currently possible.

The potential applications of SWH described so far have largely restricted attention to optical carrier frequencies. However, the broader potential of our approach can be unlocked by transferring the notion to other wave phenomena. For instance, we envision the possibility of adapting the SWH principle to ultrasound imaging of biological features embedded deep within layers of tissue. Another example is coherent X-ray diffraction imaging of specimens embedded in thick, inhomogeneous samples. In both the examples, SWH has the potential to decouple the resolution of the reconstruction (determined by the synthetic frequency) from the penetration depth (determined by the carrier frequency). We also envision the use of SWH in repurposing radio antenna arrays (e.g., the “Very Large Array” VLA) for space-based astronomical imaging at microwave and radio frequencies through dense atmosphere, and possibly below the surface of a planet for remote geological exploration. Using photonic mixers driven by continuous wave laser sources, it may be possible to simultaneously probe both optical reflectance and spectroscopic information of specimens by sensing THz synthetic wavelengths with optical detection techniques coupled together with direct sensing of THz electromagnetic signals. Moreover, we believe that the SWH concept has huge potential for material science, may it be to see deeper through materials or for the precise analysis of inhomogeneous or multi-layered structures.

There is much to be gained from exploiting spectral correlations in coherent light transport. Examining the SWH approach through the lens of Gabor holography provides newer insights into its operation and scope. Gabor originally conceived holography as a two-step process that involved recording an electron wave hologram and subsequently replaying it via optical diffraction. SWH can be interpreted as a generalization of Gabor’s original analysis/synthesis technique, with an additional computation step. We hope that this alternative view of SWH will help usher a diverse array of exciting research directions, in much the same manner as the invention of holography did decades ago.

## Methods

### Interferometer design and lock-in detection of the synthetic hologram

In practice, poor signal-to-background or signal-to-noise ratios, or both, can limit our ability to measure objects at the smallest possible SWL that is defined by the RQWR (Eq. (3)). Interferometric approaches exploiting frequency heterodyning have particularly advantageous properties with respect to this problem. The principal benefit of adopting these approaches to record holograms is the ability to exploit the heterodyne gain^52^ afforded by the use of a strong reference beam, whose baseband optical frequency is slightly detuned from the frequency of light in the object arm. The difference in frequency *ν*_m_ is chosen in the RF frequency range (3 kHz for our experiments) and realized by using a cascade of acousto-optic or electro-optic modulators (AOM or EOM). Fig. 4a, b in the Supplementary Material depicts the two interferometer designs that we use to acquire the holograms at the two optical wavelengths. Each design is an adaptation of a Michelson Interferometer, and incorporates a small difference *ν*_m_ in the baseband frequency of light in the two arms of the interferometer. It is emphasized that the RF modulation frequency *ν*_m_ is *fully decoupled* from the choice of SWL (and therefore from the resolution of our method!), and can be chosen independent of the SWL.

A Lock-In Focal Plane Array camera (LI-FPA)^51^ capable of synchronously demodulating the received irradiance at each detector pixel, is operated to detect the RF frequency *ν*_m_. The process directly yields the interferogram at the SWL Λ. The method avoids the need for time consuming raster scanning as necessary in ToF-based techniques, and phase-shifting of the optical signal. It also vastly improves the Signal-to-Background ratio of our measurements by suppressing the unmodulated ambient illumination. The Heliotis C3 LI-FPA^51^ used in our experiments yields a 300 × 300pix image per measurement. The exposure time of each measurement is *t*_exp_ = 23 ms corresponding to 70 cycles of the RF frequency *ν*_m_ = 3 kHz. Two independently tunable narrow linewidth CW lasers (Toptica DFB pro 855 nm) are used to illuminate and interrogate the scene. The center wavelength of each laser is 855 nm, and the maximum tuning range is ~2.6 nm. This allows us to achieve SWLs Λ ⪆ 300 μm, corresponding to a beat frequencies ⪅ 1THz.

The holograms in our proof-of-principle experiments were recorded using two specific heterodyne interferometer architectures: a dual-wavelength heterodyne interferometer (Fig. 4a in the Supplementary Material), and a superheterodyne interferometer (Fig. 4b in the Supplementary Material). The dual-wavelength heterodyne interferometer is preferred when light loss in the interferometer should be minimized, which is important for many NLoS applications. Light from the two lasers operating at *λ*_1_, *λ*_2_ is coupled together, before being split into the reference and sample arm. The reference arm is additionally modulated by *ν*_m_ = 3 kHz, using a cascade of two fiber AOM’s. During acquisition, each laser is shuttered independently and the lock-in camera records the holograms at the two optical wavelengths, in a time-sequential manner. The LI-FPA provides two images: in-phase (I) and quadrature (Q), each of which represents the real and imaginary parts of the speckle fields incident on the image sensor. The expression for the I- and Q-images recorded by the LI-FPA for the wavelength *λ*_*n*_ is:

4 $${I}_{I}({\lambda }_{n})= \, {A}_{n}\cos (\phi ({\lambda }_{n}))\\ {I}_{Q}({\lambda }_{n})= \, {A}_{n}\sin (\phi ({\lambda }_{n})),$$

where *A*_*n*_ is the amplitude at *λ*_*n*_ and *ϕ*(*λ*_*n*_) is the difference in the phase of light in the object and reference arms. Please note that Eq. (4) omits any reference to spatial locations, in the interest of clarity. Subsequently, the synthetic hologram *E*(Λ) is assembled as follows:

5 $$E({{\Lambda }})=	 \, [{I}_{I}({\lambda }_{1})+i{I}_{Q}({\lambda }_{1})]\cdot {[{I}_{I}({\lambda }_{2})+i\cdot {I}_{Q}({\lambda }_{2})]}^{* }\\ =	 \, {A}_{1}{A}_{2}\exp (i\underbrace{(\phi ({\lambda }_{1})-\phi ({\lambda }_{2}))}_{{\phi ({{\Lambda }})}})$$

An attractive feature of the time-sequential approach to hologram acquisition described above is that it does not require the use of two tunable lasers. Identical results can be achieved with one laser that is tuned between the two measurements. Possible extensions include: one tunable and one fixed wavelength laser, and one fixed wavelength laser that is split in two arms, one of which includes an additional frequency modulator.

Unfortunately, the simplicity of the time-sequential approach comes at the expense of increased sensitivity to object motion between measurements, and time-varying fluctuations in the environmental conditions. Increased robustness to these fluctuations is afforded by the Superheterodyne Interferometer design, wherein light from both lasers is used to simultaneously illuminate the target and scene. A possible realization is shown in Fig. 4b of the Supplementary Material: each laser beam is split into two arms, where one of which is independently modulated with an AOM. The RF drive frequencies for AOMs 1A and 1B (see Fig. 4b in the Supplementary Material) are identically set to *ν*_AOM1_, but include a phase offset Δ*φ*_AOM_ that is user controlled. Light leaving the two AOMs is combined and modulated with a third AOM (frequency *ν*_AOM2_), which produces the desired modulation frequency *ν*_m_ = *ν*_AOM1_ − *ν*_AOM2_ = 3 kHz. The expression for the I- and Q-images (in-phase and quadrature) recorded by the LI-FPA after locking in at *ν*_m_ are:

6 $${I}_{I}({\lambda }_{1},{\lambda }_{2})= \, {A}_{1}\cos (\phi ({\lambda }_{1})+{{\Delta }}{\varphi }_{{{{{{\mathrm{AOM}}}}}}})+{A}_{2}\cos (\phi ({\lambda }_{2}))\\ {I}_{Q}({\lambda }_{1},{\lambda }_{2})= \, {A}_{1}\sin (\phi ({\lambda }_{1})+{{\Delta }}{\varphi }_{{{{{{\mathrm{AOM}}}}}}})+{A}_{2}\sin (\phi ({\lambda }_{2}))$$

The synthetic hologram *E*(Λ) is assembled by calculating:

7 $$\, {I}_{I}^{2}+{I}_{Q}^{2} \, ={A}_{1}^{2}+{A}_{2}^{2}+{A}_{1}{A}_{2}\cos (\underbrace{\phi ({\lambda }_{1})-\phi ({\lambda }_{2})}_{\phi ({{\Lambda }})}+{{\Delta }}{\varphi }_{{{{{{\mathrm{AOM}}}}}}})$$

The synthetic phase map is eventually recovered from the interferograms recorded with three or more phase shifts Δ*φ*_AOM_ introduced between measurements. It should be emphasized that the use of two tunable lasers is also not a pre-requisite for this approach. Identical results can be achieved with one fixed and one tuned laser, or similar combinations discussed above. The principal benefit of the superheterodyne approach is the robustness to environmental fluctuations and object motion. However, it requires an additional AOM and fiber splitters that significantly reduce the available output power compared to the dual wavelength heterodyne interferometer discussed previously. The loss of power presents light throughput challenges for NLoS experiments that are intrinsically light starved.

In practice, there exists a trade-off between light throughput and robustness to environmental fluctuations, which depends on multiple factors including stand-off distance, reflectivity of the involved surfaces, and laser power.

It should be mentioned that the Superheterodyne approach described above even allows for single-shot acquisition and reconstruction of the hidden object. In this case, the synthetic fringe pattern calculated from Eq. (7) is treated as an ‘synthetic amplitude-only hologram’ that can be backpropagated with the SWL to reconstruct the object. Multiple exposures with different phaseshifts Δ*φ*_AOM_ are not required. However, this capability comes at the expense of having a twin-term which could potentially be digitally eliminated by further filtering.

### Reference beam injection with reduced radiometric losses

The reference beam required for interferometric sensing of the speckle fields at the optical wavelengths is directed towards the lock-in FPA. In one possible embodiment, a lensed fiber needle (WT&T Inc.) positioned in the front focal plane of the imaging optic (see Fig. 4f in the Supplementary Material) produces a near planar reference beam on the FPA. The use of a lensed fiber provides two distinct advantages over a beam-splitter: (1) the imaging optic can be directly threaded to the camera (eliminates the need for inserting beam splitter between optic and sensor) and easily swapped during operation, and (2) improved light throughput (see Table 1).

**Table 1:** Light loss at beam combiner (reference and sample arm): lensed fiber needle vs. conventional 50/50 beam splitter.

Light loss in:	Reference beam (%)	Sample beam (%)
Lensed fiber needle	~30	~0
50/50 Beam splitter	~50	~50

### Experimental setup and image formation for reflective NLoS imaging (looking around corners)

The experimental apparatus schematically displayed in Fig. 1b, and shown in Fig. 4c of the Supplementary Material is used to demonstrate the ability of SWH to discern objects obscured from view, in this case a cutout of the character ‘N’ with dimensions ~20 mm × 15 mm. The size of the object was deliberately chosen to be smaller than the typical size of a resolution cell (~2 cm) in competing wide-field ToF-based approaches. The disadvantage when using a small object is that it emits less light than the background. The problem is additionally compounded by the limited laser power in the object arm (~30 mW). In an effort to bypass these engineering limitations, we glued a thin sheet of silver foil to the sandblasted (280 grit) surface of the object ‘N’ and repeated the process for the VS surface. An image of object ‘N’ under ambient light can be seen in Fig. 2q. The fields reflected by these materials are fully developed speckle patterns. The VD wall surface is constructed from a standard dry-wall panel that has been painted white (Beer Eggshell paint).

Our approach to reflective NLoS imaging relies on the availability of an intermediary scattering surface (such as the wall in Fig. 1b) that serves to indirectly illuminate the obscured target and intercept the light scattered by the target. Accordingly, the intermediary surface may be viewed as a virtualized source (VS) of illumination and a virtualized detector (VD) for the obscured object.

Laser light from the physical source (at wavelengths *λ*_1_ and *λ*_2_) is directed towards the VS surface using a focusing optic. This light is scattered by the VS surface so as to illuminate the obscured object with a fully developed objective speckle pattern. A fraction of the light incident on the obscured object is redirected towards the VD surface. A second scattering event at the VD surface directs a tiny fraction of the object light towards the collection aperture, and subsequently the LI-FPA. The speckle fields impinging on the LI-FPA are synchronously demodulated to recover the real and imaginary parts of the holograms at the optical wavelengths *λ*_1_ and *λ*_2_. Each of these holograms is additionally subject to diffraction due to the finite collection aperture. However, the diffraction effects are observed at optical wavelengths and have little impact on the SWL Λ. After assembling the synthetic hologram, the hidden object can be reconstructed by backpropagating the synthetic hologram, using a propagator (Free-Space propagator) at the SWL Λ. In the shown experiments, we used an angular spectrum propagator.

Figure 2 includes the result of processing the NLoS measurements acquired using the experimental setup of Fig. 1b. The measurements were captured at different SWLs ranging from 280 μm to 2.6 mm. Figure 2 shows five exemplary results for Λ = 1.30 mm, Λ = 920 μm, Λ = 610 μm, Λ = 560 μm, and Λ = 440 μm. The phase of the synthetic hologram associated with each SWL is shown in Fig. 2a–e. The phasemaps have been low-pass filtered with kernel size ≈ Λ for better visualization.

The reconstruction resolution improves with decreasing SWL. However, decreasing the SWL leads to an increased spectral decorrelation of the speckle fields at the two optical wavelengths. The decorrelation manifests as excessive phase fluctuations in the SWH, which in turn produces increased speckle artifacts in the reconstructed images. The problem can be mitigated (to an extent) by exploiting speckle diversity at the VS, specifically by averaging over multiple speckle realizations of the virtualized illumination. In our experiment, we realized the speckle diversity by small movements of the VS position. The image insets in Fig. 2f–j represent the result of incoherent averaging (intensity-averaging) of the backpropagated images, for five different VS positions. The improvement in reconstruction quality comes at the expense of increased number of measurements, but not unlike competing ToF-based approaches (e.g. >20,000 VS positions are used in Liu et al.^20^). The distinction is that we need far fewer images. We conclude our discussion by observing that for static objects, the reconstruction quality may be further improved by increasing the number of VS positions used to realize speckle diversity.

### Synthetic diffraction discs and lateral resolution

As seen in Fig. 2f–j, the resolution of the NLoS reconstruction improves with decreasing SWL Λ. This behavior is in complete agreement with results from classical holography. The diffraction limited resolution (minimum resolvable spot radius *δx*) of SWH can be quantified using Eq. (2), which succinctly captures the relationship between the SWL Λ and the highest resolution that can be achieved. A smaller SWL is clearly desirable since it leads to higher resolution *or* allows for a smaller VD surface (probing area) while keeping the resolution constant. We experimentally validate the above claim (and Eq. (2)) by localizing a point-like source in the hidden volume, using a VD diameter of only *D* = 58 mm. An exposed fiber connector positioned *z* = 95 mm behind the VD surface serves as a point-source. Holograms at the VD surface acquired with multiple optical wavelengths are processed to recover a multitude of synthetic holograms, each of which is digitally replayed to recover an image of the point-source. The experimentally observed spot sizes or ‘synthetic diffraction discs’, shown in Fig. 2n–p, are consistent with theoretical predictions (green circles, calculated from Eq. (2)), and increase with increasing SWL. For a SWL of 280 μm, we are able to achieve sub-millimeter resolution around the corner.

### Experimental setup and image formation for transmissive NLoS imaging

The experimental apparatus schematically displayed in Fig. 1c is used to demonstrate the ability of SWH to image through scattering media. In a first experiment, we illuminate and image the character ‘U’ (see Fig. 2q) through an optically rough ground glass diffuser (220 grit). The geometry is unlike other transmission mode experiments wherein the object is illuminated directly^33^ or sandwiched between two diffusers. The current choice of geometry is deliberate and designed to mimic the imaging of a target embedded in a scattering medium. Measurements were acquired for different SWLs ranging from 280 μm to 2.6 mm. Figure 3b–e shows four exemplary reconstructions for Λ = 1.30 mm, Λ = 800 μm, Λ = 360 μm, and Λ = 280 μm. In each instance, we incoherently averaged the reconstruction results for two VS positions. A comparison of the image insets in Fig. 3 confirms the increased decorrelation for decreasing SWL. The smallest possible SWL for a completely speckle artifact free reconstruction was quantified for this experiment to Λ = 300 μm (see Supplementary Sec. 6), and hence the results for Λ = 280 μm demonstrate performance close to the limit expressed by Eq. (3).

In a second experiment the ground glass diffuser within the imaging path is swapped with a milky plastic plate of ~4 mm thickness. The plastic plate exhibits pronounced multiple scattering, representative of imaging through volumetric scatter. Figure 3a compares the visibility of a checkerboard viewed through the 220 grit ground glass diffuser and the plastic plate. In both cases, the checkerboard is positioned 1*cm* under the scattering plate and viewed under ambient illumination. It is evident from Fig. 3a that the visibility of the checkerboard pattern is vastly diminished when viewed through the plastic plate, whereas the pattern is still visible when viewed through the diffuser.

Figure 3f–i shows reconstruction results for the same character ‘U’ as imaged through the plastic plate, for the same set of SWLs as the diffuser. In each instance, we incoherently averaged the reconstruction results for two VS positions. The character is reconstructed with high fidelity despite pronounced multiple scattering, suggesting the potential of SWH for imaging through volumetric scatter. A comparison of the image insets in Fig. 3 confirms the diminished fidelity of imaging through volumetric scattering when compared to surface scatter.

## Supplementary information

Supplementary Information

## Data Availability

Measured raw data used to produce Figs. 1, 2, 3, and 4 are available upon request. Please contact the corresponding author.

## Code availability

Computer code, used to produce Figs. 1, 2, 3, and 4 is available upon request. Please contact the corresponding author.
