# Supplementary material for: Fast non-line-of-sight imaging with high-resolution and wide field of view using synthetic wavelength holography
Source: Nat Commun. 2021 Nov 17;12:6647. doi: 10.1038/s41467-021-26776-w (PMC8599621; doi:10.1038/s41467-021-26776-w)
Supplement: Supplementary file 1 — Supplementary Information [file 41467_2021_26776_MOESM1_ESM.pdf]

Supplementary Information for the manuscript
"Fast Non-Line-of-Sight Imaging with High-Resolution and Wide Field of View using Synthetic Wavelength Holography"

Supplementary Introduction

It is common knowledge in holography and interferometry that peak-to-valley excursions in the wavefront error (wave aberration) that are limited to ¼ of the optical wavelength (Rayleigh criterion) introduce minimal errors in the retrieved intensity and phase of an optical field. However, it is not apparent that computational mixing of scattered fields recorded at two closely spaced wavelengths λ_1, λ_2, each of which exhibits wavefront aberrations far in excess of ¼ of the optical wavelength, do indeed preserve phase information at scales smaller than the synthetic wavelength $\Lambda = \frac{|\lambda_1 - \lambda_2|}{\lambda_1 \lambda_2}$. The present document puts forth a mathematical basis for the aforementioned claim. The analysis accommodates scattering at optically rough surfaces, optical blurring in an imaging optic and sampling at the detector. The analysis while broad in scope, focuses on the application of imaging objects that are beyond the imager line-of-sight (LoS) and obscured from view. The canonical scene arrangement of **Supplementary Figure1** is used to introduce relevant concepts.

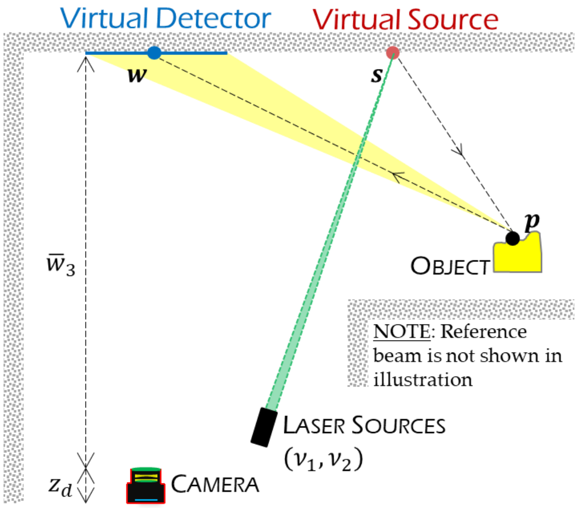

Supplementary Figure1: Canonical Scene arrangement for indirect imaging of obscured objects

Our approach exploits the availability of an intermediary scattering surface (such as the wall in **Supplementary Figure1**) in serving the dual purpose of illuminating objects obscured from view and intercepting the light scattered by the obscured objects. Accordingly, the intermediary surface may be viewed as a virtualized source of illumination and detection for the obscured objects. Herein, we seek to simultaneously recover a spatially resolved image of the obscured objects and their position within the hidden volume. To this end, we record the optical field emerging from the intermediary surface, using a holographic imaging apparatus (camera in **Supplementary Figure1**). The recorded hologram, however, bears little resemblance to the macroscopic structure of the obscured object, due in large part to the randomzied dephasing of light following a reflection at the intermediary surface. Circumventing this problem without any knowledge of the scattering properties of the intermediary surface is a key

contribution of our work. It is made possible by interrogating the hidden scene using two closely spaced optical frequencies and exploiting the spectral correlation in scattering at the intermediary surface.

The notion of exploiting spectral correlations in mitigating the deleterious effects of randomzied dephasing due to repeated scattering, has broad applications ranging from imaging through scattering/turbid media, imaging through tissue, imaging through fog/smoke, and imaging under brownout conditions. This document describes the mathematical principles underpinning the use of spectral field correlations in adapting scattering as degree of freedom from the standpoint of imaging.

In the interest of clarity, attention is restricted to the modeling the propagation of coherent light in the canonical scene of **Supplementary Figure1**, and the subsequent recovery of a hologram of the obscured object(s). The task of accurately modeling light propagation within the canonical scene is exceedingly difficult, due in large part to the multiplicity of scattering surfaces and numerous light bounces. The task is further complicated by factors such as multiple scattering at interfaces, shadowing, and Fresnel reflections. Incorporating these effects into a comprehensive model for imaging is mathematically intractable. Imposing specific restrictions and simlyfing assumptions allows us to develop a mathematically tractable framework for light transport in the canonical scene. The list of restrictions and simplifying assumtions are enumerated below:

1. A linearly polarized narrow-linewidth tunable CW laser source with center frequency $\bar{\nu}$ is used to interrogate the hidden scene by illuminating the Virtual Souce surface.
2. Scalar diffraction is sufficient to model field transport through the scene.
3. The temporal fluctuations of the CW laser source are statistically uncorrelated with those of ambient light sources in the scene.
4. The principal contribution to the irradiance observed at the sensor is restricted to three bounce ray paths originating at the physical source, bouncing off the Virtual Source surface, the hidden object and the Virtual Detector surface, prior to terminating at the physical detector. The radiometric throughput of fourth and higher order bounces is assumed to be negligibly small, a fact borne out in experiments.
5. The coherence length of the source exceeds the cumulative length of all three-bounce ray originating at the physical source, traversing the hidden scene and terminating at the physical sensor. Consequently, the indirectly illuminated object can be expressed as a countably-finite collection of secondary point sources that are mutually coherent.
6. The propagation medium is free space and devoid of inhomogenities. The propagation distances exceed the spatial extent of the Virtual Source and the Virtual Detector.
7. Physical objects in the scene are optically rough at the scale of the optical wavelength of the CW laser source. We assume that path length variations induced by fluctuations in the surface height are the sole source of scattering, consistent with Goodman's approach [1].
8. A diffraction limited optic is used to relay the image of the Virtual Detector surface onto an image sensor. The coordinate system used to develop the model is centered about entrance pupil of the imaging optic, XY plane is aligned with image sensor and Z-axis is aligned with the optical axis
9. A lock-in sensor whose operation is described in [2], records interference of light scattered by obscured object and a planar reference beam. The lock-In camera (Lock-In sensor + imaging optics) independently acquires holograms of coherently illuminated scene/object
10. The reference beam envelope and phase does not change appreciably over the finite extent of a single detector pixel.

11. The illumination source subtends a small solid angle with respect to the obscured object. The spectral reflectance of source is unchanged for small change in optical frequency of illumination source.
12. The object albedo is unchanged for small change in optical frequency of illumination source
13. The defocus error introduced by the microscopic roughness of the Virtual Detector surface is negligible.

Key findings/claims

1. A small change $\Delta\nu$ in the optical frequency of the CW source used to interrogate the hidden scene of **Supplementary Figure1** imparts an additional spherical phase component to the field contribution of each obscured object point. The excess spherical phase encodes the position of the obscured object at the "synthetic" wavelength $c\Delta\nu^{-1}$ meters. **(Eqs.(28),(29))**
2. Computational mixing of holograms recorded at two closely spaced optical frequencies encapsulates field information at sub-millimeter synthetic wavelength scales that are insensitive to scattering at the optical frequency of interrogation. **(Sections.1.5,1.6)**
3. Wavefront errors in the computational hologram are negligible if the change in optical path length stemming from a change $\Delta\nu$ in the optical frequency of interrogation is limited to $\frac{1}{4^{\text{th}}}$ synthetic wavelength $c\Delta\nu^{-1}$ meters, for any ray path originating at the source and terminating at the sensor. **(Section.1.6)**
4. Optical holograms acquired at regularly spaced optical frequencies may be combined to computationally filter light paths with a prescribed round-trip distance from the physical source to the physical detector, so long as condition-3 holds for the largest frequency separation. **(Section.2)**

Notation

The mathematical analysis furnished in this document oftentimes involves multiple integrals and summations over spatial and time dimensions that can be continuous or discrete. In an effort to improve the clarity of the analysis we adopt the following convention in describing quantities of interest.

$\boldsymbol{x}$	Position vector of a point on the sensor plane	$\in \mathbb{R}^2$	meters
$\boldsymbol{d}$	Position vector associated with a point on the Virtual Detector	$\in \mathbb{R}^3$	meters
$\boldsymbol{s}$	Position vector associated with a point on the Virtual Source	$\in \mathbb{R}^3$	meters
$\boldsymbol{p}$	Position vector associated with the obscured object point	$\in \mathbb{R}^3$	meters
$\bar{\nu}$	Optical frequency of the CW source used to interrogate the hidden scene		THz
$\bar{\omega} = 2\pi\bar{\nu}$	Angular frequency of the CW source used to interrogate the hidden scene		radians
t	Time		seconds
I	Irradiance (real valued & non-negative)	$\in \mathbb{R}^{2+}$	arbitrary units
$\mathcal{U}$	Optical field (complex-valued)	$\in \mathbb{C}^2$	arbitrary units
Δ	Sensor pixel pitch (in μm)		Microns
F	Fill factor of sensor pixel ($0 < F \leq 1$)	$\in \mathbb{R}^+$	dimensionless

Key expressions

Key expressions from the mathematical analysis are summarized in **Supplementary Figure2**. These include the expression for the sampled holograms at two closely optical frequencies, and a mathematical formulation of Claim-1.

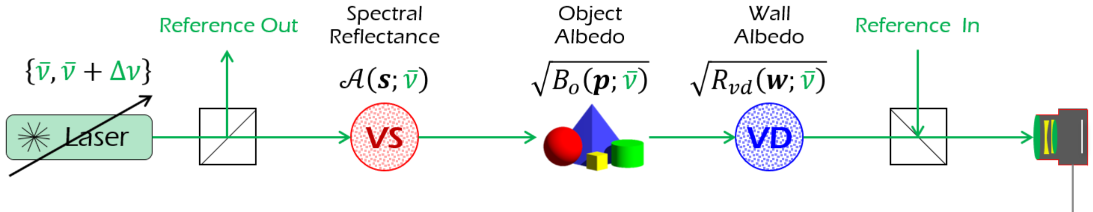

Sampled hologram at $\bar\nu$

$$\mathcal{U}_\Delta[\boldsymbol{n};\bar\nu] = \frac{N}{\nu_b}\mathcal{U}_r^*[\boldsymbol{n};\bar\nu]\left\{\int d\boldsymbol{p}\left(\mathcal{U}_{\text{ill}}(\boldsymbol{p};\bar\nu)\sqrt{B_o(\boldsymbol{p};\bar\nu)}\left(\int_{(\boldsymbol{n}-0.5F)\Delta}^{(\boldsymbol{n}+0.5F)\Delta} d\boldsymbol{x}\; h_{vd}(\boldsymbol{x};\boldsymbol{p},\bar\nu)\right)\right)\right\}$$

Indirect illumination $\mathcal{U}_{\text{ill}}(\boldsymbol{p};\bar\nu)$ represents complex-valued illumination incident on the object point $\boldsymbol{p}$, due to scattering at the Virtual Source.	$\overset{\text{def}}{=} \int d\boldsymbol{s}\,\mathcal{A}(\boldsymbol{s};\bar\nu)\frac{\exp\left(i2\pi\frac{\bar\nu}{c}[\Vert\boldsymbol{s}-\boldsymbol{p}\Vert]\right)}{\Vert\boldsymbol{s}-\boldsymbol{p}\Vert}\psi(\boldsymbol{s},\boldsymbol{p})$
Indirect Imaging PSF $h_{vd}(\boldsymbol{x};\boldsymbol{p},\bar\nu)$ represents Point-spread-function (PSF) of light transport from the obscured object point $\boldsymbol{p}$ to a point $\boldsymbol{x}$ on the sensor plane, following a scattering event at the Virtual Detector	$\overset{\text{def}}{=} \int d\boldsymbol{w}\frac{\exp\left(i2\pi\frac{\bar\nu}{c}\Vert\boldsymbol{p}-\boldsymbol{w}\Vert\right)}{\Vert\boldsymbol{p}-\boldsymbol{w}\Vert}\psi(\boldsymbol{p},\boldsymbol{w})\sqrt{R_{vd}(\boldsymbol{w};\bar\nu)}\,h_{\text{blur}}(\boldsymbol{x};\boldsymbol{w},\bar\nu)$

Sampled hologram at $\bar\nu+\Delta\nu$

$$\mathcal{U}_\Delta[\boldsymbol{n};\bar\nu+\Delta\nu] = \left(\frac{\bar\nu+\Delta\nu}{\bar\nu}\right)^4\frac{N}{\nu_b}\mathcal{U}_r^*[\boldsymbol{n};\bar\nu]\exp(i\phi[\boldsymbol{n};\Delta\nu])\left\{\int d\boldsymbol{p}\left(\exp(i\theta[\boldsymbol{n};\boldsymbol{p},\Delta\nu])\left(\mathcal{U}_{\text{ill}}(\boldsymbol{p};\bar\nu)\sqrt{B_o(\boldsymbol{p};\bar\nu)}\left(\int_{(\boldsymbol{n}-0.5F)\Delta}^{(\boldsymbol{n}+0.5F)\Delta} d\boldsymbol{x}\; h_{vd}(\boldsymbol{x};\boldsymbol{p},\bar\nu)\right)\right)\right)\right\}$$

Scene independent phasor $\phi[\boldsymbol{n};\Delta\nu]$ represents change in OPL associated with light transport from $\boldsymbol{n}^{th}$ Virtual Detector pixel to the $\boldsymbol{n}^{th}$ sensor pixel	$\overset{\text{def}}{=} 2\pi\frac{\Delta\nu}{c}\left(z_d+\frac{\Vert\boldsymbol{n}\Delta\Vert^2}{2z_d}+\bar w_3+\frac{\Vert z_d^{-1}\bar w_3\boldsymbol{n}\Delta\Vert^2}{2\bar w_3}\right)$	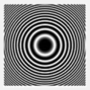 Quadratic Phase
Scene dependent phasor $\theta[\boldsymbol{n};\boldsymbol{p},\Delta\nu]$ represents change in OPL of all ray paths originating at Virtual Source, bouncing off the obscured object and terminating at the $\boldsymbol{n}^{th}$ Virtual Detector pixel	$\overset{\text{def}}{=} 2\pi\frac{\Delta\nu}{c}\left(\Vert\bar{\boldsymbol{s}}-\boldsymbol{p}\Vert+\left\Vert\boldsymbol{p}-\left[\frac{-z_d^{-1}\bar w_3\boldsymbol{n}\Delta}{\bar w_3}\right]\right\Vert\right)$	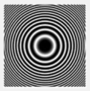 Quadratic Phase

Computational Mixing

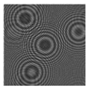

Latent Hologram

$$\mathcal{U}_{\text{latent}}[\boldsymbol{n};\Delta\nu] = \left(\left(\frac{\bar\nu+\Delta\nu}{\bar\nu}\right)^4\left(\frac{N}{\nu_b}\right)^2 I_r[\boldsymbol{n};\bar\nu]\right)\exp(i\phi[\boldsymbol{n};\Delta\nu])\left\{\int d\boldsymbol{p}\left(\left(|\mathcal{U}_{\text{ill}}(\boldsymbol{p};\bar\nu)|^2B_o(\boldsymbol{p};\bar\nu)\left|\int_{(\boldsymbol{n}-0.5)\Delta}^{(\boldsymbol{n}+0.5)\Delta}d\boldsymbol{x}\,h_{vd}(\boldsymbol{x};\boldsymbol{p},\bar\nu)\right|^2\right)\exp\left(i2\pi\frac{\Delta\nu}{c}\left[\Vert\bar{\boldsymbol{s}}-\boldsymbol{p}\Vert+\left\Vert\boldsymbol{p}-\left[\frac{-z_d^{-1}\bar w_3\boldsymbol{n}\Delta}{\bar w_3}\right]\right\Vert\right]\right)\right)\right\}$$

Supplementary Figure2: Key mathematical expressions

The terms $\psi(\boldsymbol{s},\boldsymbol{p}),\psi(\boldsymbol{p},\boldsymbol{w})$ represent the obliquity factors associated with field transport from the Virtual Source to the object, and the object to the Virtual Detector. The term N represents the number of periods of the Local Oscillator signal over which the Lock-In sensor operates, to compute the sampled hologram.

In the interest of clarity, the task of modeling light transport in the canonical scene of **Supplementary Figure1** is sub-divided into two tasks. The first of these tasks seeks to model the process of imaging the Virtual Detector surface including sampling at the image sensor. The second task models the physical propagation of light originating at the Virtual Source, bouncing off the obscured object and terminating at the Virtual Detector.

1.1 Imaging identity and optical blur

Without loss of generality, it is assumed that light emerging from the Virtual Detector surface is relayed to the Lock-In sensor using a well-corrected optic with focal length f and unity pupil magnification. The relation between a point on the Virtual Detector surface and its pixel projection on the image sensor satisfies the following geometric relation:

$$x_1 = -z_d \frac{w_1}{w_3} \quad , \; x_2 = -z_d \frac{w_2}{w_3} \tag{1}$$

The term z_d represents the distance from the sensor plane to the exit pupil plane of the imaging optics. The negative sign accommodates image inversion. If the imager optical axis and the macroscopic normal vector to the Virtual Detector surface (disregarding change in local surface normal due to microscopic roughness of VD surface) are aligned, then the magnification $z_d^{-1}w_3 \approx z_d^{-1}\overline{w}_3$, where $\overline{w}_3$ is the macroscopic perpendicular distance from the VD surface to the entrance pupil plane of the imaging optics.

The diffraction limited optical blur associated with imaging the VD surface may be modeled as a paraxial blur [3] with image side numerical aperture Dz_d^{-1}, where D is the diameter of the exit pupil. The resulting amplitude PSF is disclosed in Eq.(2). The term $P(u,v)$ represents the transmittance function of the exit pupil and modeled as an indicator function with diameter D. The term f represents the focal length of the imaging optic. The depth w_3 of a point on the VD surface is measured with respect to the entrance pupil plane of the imaging optic.

$$\begin{aligned} &\mathrm{h_{blur}}\left(\boldsymbol{x} \stackrel{\text{def}}{=} \begin{bmatrix} x_1 \\ x_2 \end{bmatrix}; \boldsymbol{w} \stackrel{\text{def}}{=} \begin{bmatrix} w_1 \\ w_2 \\ w_3 \end{bmatrix}, \bar{\nu}\right) \\ &= \left(\begin{array}{c} \left(\frac{\bar{\nu}}{ic}\right)^2 \frac{1}{z_d w_3} \exp\left(i\frac{2\pi\bar{\nu}}{c}\left[z_d + w_3 + \frac{[x_1^2 + x_2^2]}{2z_d} + \frac{[w_1^2 + w_2^2]}{2w_3}\right]\right) \times \\ \int dudv \left(P(u,v) \exp\left(i\frac{2\pi\bar{\nu}}{c}\left[\frac{1}{z_d} + \frac{1}{w_3} - \frac{1}{f}\right](u^2+v^2)\right) \exp\left(-i\frac{2\pi\bar{\nu}}{c}\left(\left[\frac{x_1}{z_d} + \frac{w_1}{w_3}\right]u + \left[\frac{x_2}{z_d} + \frac{w_2}{w_3}\right]v\right)\right)\right) \end{array} \right) \end{aligned} \tag{2}$$

1.2 Recording optical fields using the Lock-In sensor

As is common practice in holography and interferometry, we acquire optical fields by recording the interference of the said field with a reference beam whose temporal fluctuations are highly correlated with the desired optical field. To this end, the field incident on the Lock-In sensor may be expressed as the superposition of the field contributions from the indirectly illuminated object and a planar reference beam, as shown below:

$$\mathcal{U}(\boldsymbol{x},t;\bar{\nu}) = \mathcal{U}_o(\boldsymbol{x};\bar{\nu}) \exp(i\bar{\omega}t) + \mathcal{U}_r(\boldsymbol{x};\bar{\nu}) \exp(i[\bar{\omega} + \omega_b]t) \tag{3}$$

The term $\mathcal{U}_r(\boldsymbol{x};\bar{\nu})$ in Eq.(3) represents the baseband envelope of the reference field incident on the Lock-in sensor plane. The term $\mathcal{U}_o(\boldsymbol{x};\bar{\nu})$ represents the baseband envelope of the subjective speckle field due to the indirectly illuminated object/target. The angular frequency difference ω_b between the two arms of the interferometer helps in isolating the desired field component from the corrupting influence of the zeroth order and twin components that arise during square-law detection of the irradiance $|\mathcal{U}(\boldsymbol{x},t;\bar{\nu})|^2$. Insight into the process can be gleaned by examining the expression for the instantaneous irradiance incident on the Lock-In sensor plane, disclosed in Eq.(4).

$$\begin{aligned} I(\boldsymbol{x},t;\bar{\nu}) &= |\mathcal{U}(\boldsymbol{x},t;\bar{\nu})|^2 \\ &= |\mathcal{U}_o(\boldsymbol{x};\bar{\nu})|^2 + |\mathcal{U}_r(\boldsymbol{x};\bar{\nu})|^2 + 2\,\mathcal{R}eal\{\mathcal{U}_o(\boldsymbol{x};\bar{\nu})\mathcal{U}_r^*(\boldsymbol{x};\bar{\nu})\exp(-i\omega_b t)\} \\ &= |\mathcal{U}_o(\boldsymbol{x};\bar{\nu})|^2 + |\mathcal{U}_r(\boldsymbol{x};\bar{\nu})|^2 + 2|\mathcal{U}_o(\boldsymbol{x};\bar{\nu})| \times |\mathcal{U}_r(\boldsymbol{x};\bar{\nu})|\cos(\varphi_o(\boldsymbol{x};\bar{\nu}) - \varphi_r(\boldsymbol{x};\bar{\nu}) - \omega_b t) \\ &= I_o(\boldsymbol{x};\bar{\nu}) + I_r(\boldsymbol{x};\bar{\nu}) + 2\sqrt{I_o(\boldsymbol{x};\bar{\nu})}\sqrt{I_r(\boldsymbol{x};\bar{\nu})}\cos(\varphi_o(\boldsymbol{x};\bar{\nu}) - \varphi_r(\boldsymbol{x};\bar{\nu}) - \omega_b t) \end{aligned} \tag{4}$$

The instantaneous intensity recorded by the $\boldsymbol{n}^{th}$ Lock-In pixel is obtained by integrating the irradiance $I(\boldsymbol{x},t;\bar{\nu})$ incident on its active area. The corresponding expression is disclosed below:

$$\begin{aligned} I[\boldsymbol{n},t;\bar{\nu}] &= \int_{(\boldsymbol{n}-0.5F)\Delta}^{(\boldsymbol{n}+0.5F)\Delta} d\boldsymbol{x}\, I(\boldsymbol{x},t;\bar{\nu}) \\ &= \left\{\int_{(\boldsymbol{n}-0.5F)\Delta}^{(\boldsymbol{n}+0.5F)\Delta} d\boldsymbol{x}\,[I_o(\boldsymbol{x};\bar{\nu}) + I_r(\boldsymbol{x};\bar{\nu})]\right\} + \left\{2\int_{(\boldsymbol{n}-0.5F)\Delta}^{(\boldsymbol{n}+0.5F)\Delta} d\boldsymbol{x}\left[\sqrt{I_o(\boldsymbol{x};\bar{\nu})}\sqrt{I_r(\boldsymbol{x};\bar{\nu})}\cos(\varphi_o(\boldsymbol{x};\bar{\nu}) - \varphi_r(\boldsymbol{x};\bar{\nu}) - \omega_b t)\right]\right\} \end{aligned} \tag{5}$$

Following assumption-6, the envelope and phase of the reference beam does not change appreciably over the finite extent of a single detector pixel, so that:

$$\left.\begin{aligned} I_r(\boldsymbol{x};\bar{\nu}) \approx I_r(\boldsymbol{n}\Delta;\bar{\nu}) \stackrel{\text{def}}{=} I_r[\boldsymbol{n};\bar{\nu}] \\ \cos\big(\varphi_r(\boldsymbol{x};\bar{\nu})\big) \approx \cos\big(\varphi_r(\boldsymbol{n}\Delta;\bar{\nu})\big) \stackrel{\text{def}}{=} \cos(\varphi_r[\boldsymbol{n};\bar{\nu}]) \end{aligned}\right\} \quad \forall\, \boldsymbol{x} \in (\boldsymbol{n}\Delta - \mathbf{0.5}F\Delta, \boldsymbol{n}\Delta + \mathbf{0.5}F\Delta) \tag{6}$$

As a result, the reference beam's contribution to the integrated irradiance may be simplified as follows:

$$\left\{\int_{(\boldsymbol{n}-0.5F)\Delta}^{(\boldsymbol{n}+0.5F)\Delta} d\boldsymbol{x}\, I_r(\boldsymbol{x};\bar{\nu})\right\} \approx \left\{\int_{(\boldsymbol{n}-0.5F)\Delta}^{(\boldsymbol{n}+0.5F)\Delta} d\boldsymbol{x}\, I_r(\boldsymbol{n}\Delta;\bar{\nu})\right\} \approx I_r[\boldsymbol{n};\bar{\nu}]\left\{\int_{(\boldsymbol{n}-0.5F)\Delta}^{(\boldsymbol{n}+0.5F)\Delta} d\boldsymbol{x}\right\} \approx \Delta^2 \times I_r[\boldsymbol{n};\bar{\nu}] \tag{7}$$

Incorporating Eqs.(7) & (6) into Eq.(5) yields the following expression for the instantaneous irradiance recorded by the $\boldsymbol{n}^{th}$ lock-in pixel:

$$\begin{aligned} I[\boldsymbol{n},t;\bar{\nu}] &\approx \left\{\Delta^2 \times I_r(\boldsymbol{n}\Delta;\bar{\nu}) + \int_{(\boldsymbol{n}-0.5F)\Delta}^{(\boldsymbol{n}+0.5F)\Delta} d\boldsymbol{x}\,[I_o(\boldsymbol{x};\bar{\nu})]\right\} + \left\{2\sqrt{I_r[\boldsymbol{n};\bar{\nu}]}\int_{(\boldsymbol{n}-0.5F)\Delta}^{(\boldsymbol{n}+0.5F)\Delta} d\boldsymbol{x}\left[\sqrt{I_o(\boldsymbol{x};\bar{\nu})}\cos(\varphi_o(\boldsymbol{x};\bar{\nu}) - \varphi_r[\boldsymbol{n};\bar{\nu}] - \omega_b t)\right]\right\} \\ &\approx \left(\begin{aligned} &\left\{\Delta^2 \times I_r[\boldsymbol{n};\bar{\nu}] + \left[\int_{(\boldsymbol{n}-0.5F)\Delta}^{(\boldsymbol{n}+0.5F)\Delta} d\boldsymbol{x}\, I_o(\boldsymbol{x};\bar{\nu})\right]\right\} + \left\{2\sqrt{I_r[\boldsymbol{n};\bar{\nu}]}\cos(\omega_b t)\left[\int_{(\boldsymbol{n}-0.5F)\Delta}^{(\boldsymbol{n}+0.5F)\Delta} d\boldsymbol{x}\left[\sqrt{I_o(\boldsymbol{x};\bar{\nu})}\cos(\varphi_o(\boldsymbol{x};\bar{\nu}) - \varphi_r[\boldsymbol{n};\bar{\nu}])\right]\right]\right\} \\ &+ \left\{2\sqrt{I_r[\boldsymbol{n};\bar{\nu}]}\sin(\omega_b t)\left[\int_{(\boldsymbol{n}-0.5F)\Delta}^{(\boldsymbol{n}+0.5F)\Delta} d\boldsymbol{x}\left[\sqrt{I_o(\boldsymbol{x};\bar{\nu})}\sin(\varphi_o(\boldsymbol{x};\bar{\nu}) - \varphi_r[\boldsymbol{n};\bar{\nu}])\right]\right]\right\} \end{aligned}\right) \end{aligned} \tag{8}$$

The first term in Eq.(8) represents the zeroth-order time-invariant irradiance contribution of the hologram recorded at $\bar{\nu}$. The second and third terms in Eq.(8) represents the time-varying irradiance contribution associated with the detector integration of the real & imaginary part of the subjective speckle field $\mathcal{U}_o(\boldsymbol{x};\bar{\nu})$, respecively. The temporal carrier associated with these terms has angular frequency ω_b and phase $\frac{\pi}{2} - \varphi_r[\boldsymbol{n};\bar{\nu}]$ and $-\varphi_r[\boldsymbol{n};\bar{\nu}]$ respectively. The amplitude $\sqrt{I_r[\boldsymbol{n};\bar{\nu}]}$ of these terms describes the heterodyne gain

arising from synchronous demodulation at ω_b. The integral over spatial locations in the second and third terms signify the real and imaginary parts of the phasor sum of the subjective speckle cells that can be accommodated within the $\boldsymbol{n}^{th}$ detector pixel. The integration accommodates signal fading arising from the summation of a disproportionately large number of statistically independent speckle cells [4].

Each pixel of the Lock-in sensor functions as a homodyne reciever that accumlates the result of demodulating the received irradiance with two local oscillator (LO) signals $\sin(\omega_b t)$ and $\cos(\omega_b t)$ that are in quadrature phase. The demodulation is restricted to N periods of the LO signal, and yields a sampled representation of the in-phase (real part) & quadrature (imaginary part) components of the complex-valued optical field incident on the Lock-In sensor. The expression for the in-phase component of the optical field recorded by the Lock-In sensor is furnished below:

$$\begin{aligned}
I_I[\boldsymbol{n};\bar{\nu}] &= \int_{t=0}^{N\nu_b^{-1}} dt\,\cos(\omega_b t)\, I[\boldsymbol{n},t;\bar{\nu}] \\
&= \left\{\int_{t=0}^{N\nu_b^{-1}} dt\, cos(\omega_b t)\, cos(\omega_b t)\right\}\left\{2\sqrt{I_r[\boldsymbol{n};\bar{\nu}]}\int_{(\boldsymbol{n}-0.5F)\Delta}^{(\boldsymbol{n}+0.5F)\Delta} d\boldsymbol{x}\left[\sqrt{I_o(\boldsymbol{x};\bar{\nu})}\cos(\varphi_o(\boldsymbol{x};\bar{\nu})-\varphi_r[\boldsymbol{n};\bar{\nu}])\right]\right\} \\
&= \left\{\frac{1}{2}\int_{t=0}^{N\nu_b^{-1}} dt[1+cos(2\omega_b t)]\right\}\left\{2\sqrt{I_r[\boldsymbol{n};\bar{\nu}]}\int_{(\boldsymbol{n}-0.5F)\Delta}^{(\boldsymbol{n}+0.5F)\Delta} d\boldsymbol{x}\left[\sqrt{I_o(\boldsymbol{x};\bar{\nu})}\cos(\varphi_o(\boldsymbol{x};\bar{\nu})-\varphi_r[\boldsymbol{n};\bar{\nu}])\right]\right\} \\
&= N\nu_b^{-1}\sqrt{I_r[\boldsymbol{n};\bar{\nu}]}\left\{\int_{(\boldsymbol{n}-0.5F)\Delta}^{(\boldsymbol{n}+0.5F)\Delta} d\boldsymbol{x}\left[\sqrt{I_o(\boldsymbol{x};\bar{\nu})}\cos(\varphi_o(\boldsymbol{x};\bar{\nu})-\varphi_r[\boldsymbol{n};\bar{\nu}])\right]\right\}
\end{aligned} \tag{9}$$

The expression for the quadrature component of the optical field recorded by the Lock-In sensor is furnished below:

$$\begin{aligned}
I_Q[\boldsymbol{n};\bar{\nu}] &= \int_{t=0}^{N\nu_b^{-1}} dt\,\sin(\omega_b t)\, I[\boldsymbol{n},t;\bar{\nu}] \\
&= N\nu_b^{-1}\sqrt{I_r[\boldsymbol{n};\bar{\nu}]}\left\{\int_{(\boldsymbol{n}-0.5F)\Delta}^{(\boldsymbol{n}+0.5F)\Delta} d\boldsymbol{x}\left[\sqrt{I_o(\boldsymbol{x};\bar{\nu})}\sin(\varphi_o(\boldsymbol{x};\bar{\nu})-\varphi_r[\boldsymbol{n};\bar{\nu}])\right]\right\}
\end{aligned} \tag{10}$$

By combining the sampled in-phase and quadrature images, the sampled hologram at the optical wavelength may be reconstituted, as shown in Eq.(11).

$$\begin{aligned}
\mathcal{U}_\Delta[\boldsymbol{n};\bar{\nu}] &= I_I[\boldsymbol{n};\bar{\nu}] + \sqrt{-1}I_Q[\boldsymbol{n};\bar{\nu}] \\
&= N\nu_b^{-1}\sqrt{I_r[\boldsymbol{n};\bar{\nu}]}\left\{\int_{(\boldsymbol{n}-0.5F)\Delta}^{(\boldsymbol{n}+0.5F)\Delta} d\boldsymbol{x}\left[\sqrt{I_o(\boldsymbol{x};\bar{\nu})}\exp\big(i[\varphi_o(\boldsymbol{x};\bar{\nu})-\varphi_r[\boldsymbol{n};\bar{\nu}]]\big)\right]\right\} \\
&= N\nu_b^{-1}\sqrt{I_r[\boldsymbol{n};\bar{\nu}]}\exp(-i\varphi_r[\boldsymbol{n};\bar{\nu}])\left\{\int_{(\boldsymbol{n}-0.5F)\Delta}^{(\boldsymbol{n}+0.5F)\Delta} d\boldsymbol{x}\left[\sqrt{I_o(\boldsymbol{x};\bar{\nu})}\exp\big(i\varphi_o(\boldsymbol{x};\bar{\nu})\big)\right]\right\} \\
&= N\nu_b^{-1}\mathcal{U}_r^*[\boldsymbol{n};\bar{\nu}]\left\{\int_{(\boldsymbol{n}-0.5F)\Delta}^{(\boldsymbol{n}+0.5F)\Delta} d\boldsymbol{x}\,\mathcal{U}_o(\boldsymbol{x};\bar{\nu})\right\}
\end{aligned} \tag{11}$$

We conclude our discussion on recording optical fields by enumerating the principal benefits of a Lock-In sensor, namely:

- The ability to detect a weak sinusoidal signal buried in strong background and noise (accommodate a wide range of ambient illumination levels)
- The heterodyne gain afforded by combination of background subtraction and synchronous demodulation of the received irradiance at each pixel (eliminates need for longer exposure times and optical stabilization of interferometer)
- Eliminating need for matching the optical power in the two interferometer arms
- Snapshot hologram acquisition with maximal utilization of the limited spatial bandwidth of the FPA, (no mechanical movement or temporal phase-shifting required for isolating the hologram term).

The interested reader is referred to [5],[6] for details on the mechanics of recoding holograms using heterodyne interferometry and the Lock-In sensor.

1.3 Light transport from the Virtual Source to the Virtual Detector

The discussion in Sections-1.1,1.2 restricted attention to the process of recording optical fields. The present section is devoted to the development of a mathematical model for propagating quasi-monochromatic scalar optical fields in the canonical scene arrangement of **Supplementary Figure1**.

Our analysis begins with the observation that light scattered by the Virtual Source surface behaves as a partially coherent source of illumination for the obscured objects. Each point on the indirectly illuminated object may then be viewed as a secondary source of partially coherent light that directs spherical wavefronts towards the Virtual Detector surface. Each of these spherical wavefronts is additionally scattered by the Virtual Detector surface before being intercepted by the finite collection aperture of the imaging optic. The resulting spatial pattern is recorded by the image sensor, and exhibits a mottled appearance reminiscent of speckle [1].

The combined field contribution of the obscured object is obtained as a weighted superposition of the elementary phasors associated with light paths originating at a point $\boldsymbol{s}$ on the Virtual Source, bouncing off an obscured object point $\boldsymbol{p}$**,** and terminating at the Virtual Detector point $\boldsymbol{w}$**,** whose geometric projection onto the image sensor is $\boldsymbol{x}$. The expression for the obscured object field $\mathcal{U}_o(\boldsymbol{x};\bar{\nu})$ incident on the Lock-In sensor is disclosed below:

$$\begin{aligned}
&\mathcal{U}_o(\boldsymbol{x};\bar{\nu}) \\
&= \left(\frac{\bar{\nu}}{ic}\right)^2 \int d\boldsymbol{w} \int d\boldsymbol{p} \left(\left(\int d\boldsymbol{s}\, \mathcal{A}(\boldsymbol{s};\bar{\nu}) \frac{\exp\left(i2\pi\frac{\bar{\nu}}{c}\|\boldsymbol{s}-\boldsymbol{p}\|\right)}{\|\boldsymbol{s}-\boldsymbol{p}\|} \psi(\boldsymbol{s},\boldsymbol{p}) \right) \sqrt{B_o(\boldsymbol{p};\bar{\nu})} \frac{\exp\left(i2\pi\frac{\bar{\nu}}{c}\|\boldsymbol{p}-\boldsymbol{w}\|\right)}{\|\boldsymbol{p}-\boldsymbol{w}\|} \psi(\boldsymbol{p},\boldsymbol{w}) \right) \sqrt{R_{vd}(\boldsymbol{w};\bar{\nu})}\, h_{\text{blur}}(\boldsymbol{x};\boldsymbol{w},\bar{\nu}) \\
&= \left(\frac{\bar{\nu}}{ic}\right)^2 \int d\boldsymbol{p} \left(\int d\boldsymbol{w} \left(\int d\boldsymbol{s}\, \mathcal{A}(\boldsymbol{s};\bar{\nu}) \sqrt{B_o(\boldsymbol{p};\bar{\nu})} \psi(\boldsymbol{s},\boldsymbol{p}) \psi(\boldsymbol{p},\boldsymbol{w}) \frac{\exp\left(i2\pi\frac{\bar{\nu}}{c}[\|\boldsymbol{s}-\boldsymbol{p}\| + \|\boldsymbol{p}-\boldsymbol{w}\|]\right)}{\|\boldsymbol{s}-\boldsymbol{p}\| \times \|\boldsymbol{p}-\boldsymbol{w}\|} \right) \sqrt{R_{vd}(\boldsymbol{w};\bar{\nu})}\, h_{\text{blur}}(\boldsymbol{x};\boldsymbol{w},\bar{\nu}) \right)
\end{aligned} \tag{12}$$

The innermost integral of Eq.(12) which is colored in magenta represents the complex-valued illumination incident on the obscured object point $\boldsymbol{p}$, following scattering at the Virtual source. It encapsulates light transport from the physical source to the obscured object point. The term $\mathcal{A}(\boldsymbol{s};\bar{\nu})$ represents the spectral reflectance of the Virtual Source. It represents the combined influence of the real-valued albedo of the Virtual Source surface, and the complex-valued illumination beam incident on the Virtual Source surface. The term $\sqrt{B_o(\boldsymbol{p};\bar{\nu})}$ represents the square-root of the real-valued albedo of the object point $\boldsymbol{p}$. It encapsulates reflection from an infinitesimally small area element $d\boldsymbol{p}$ in the immediate vicinity of the object point $\boldsymbol{p}$. The term $\sqrt{R_{vd}(\boldsymbol{w};\bar{\nu})}$ represents the square-root of the real-valued albedo of a point $\boldsymbol{w}$ on the Virtual Detector surface. It encapsulates Lambertian reflection at the Virtual Detector surface. The term h_{blur} represents the

amplitude PSF of the imaging optic and encapsulates diffraction limited imaging under coherent illumination.

Accommodating scattering

The length of the ray paths $\|\boldsymbol{s}-\boldsymbol{p}\|$ encapsulate the microscopic roughness of the Virtual Source surface and the obscured object. The corresponding phase fluctuations given by $\frac{\bar{\nu}}{c}\|\boldsymbol{s}-\boldsymbol{p}\|$ induce randomized dephasing of the spherical waves arriving at the obscured object point, mainfesting as speckle illumination. In a similar fashion, the length of the ray paths $\|\boldsymbol{p}-\boldsymbol{w}\|$ encapsulate the microscopic roughness of the obscured object and the Virtual Detector surface. The corresponding phase fluctuations given by $2\pi\frac{\bar{\nu}}{c}\|\boldsymbol{p}-\boldsymbol{w}\|$ induce randomized dephasing in the spherical waves arriving at the Virtual Detector surface, irreversibly corrupting the phase of the obscured object field propagting towards the Virtual Detector surface. Phase fluctuations arising from scattering at the object are embedded in the path length calculations $\|\boldsymbol{s}-\boldsymbol{p}\|$, $\|\boldsymbol{p}-\boldsymbol{w}\|$.

Connection to conventional imaging

By decoupling ray paths from the Virtual Source → obscured object → Virtual Detector into its constituent paths namely: Virtual Source → obscured object, and obscured object → Virtual Detector, it is posssible to recast the expression for the object field contribution at the Lock-In sensor in a mathematical form that closely resembles the standard space-variant imaging formulation [7]. In particular, the field contribution of the obscured object may be expressed as a coherent superposition of stochastic patterns that are each weighted by the real-valued albedo of the obscured object:

$$\mathcal{U}_o(\boldsymbol{x};\bar{\nu}) = \int d\boldsymbol{p}\left[\mathcal{U}_{\text{ill}}(\boldsymbol{p};\bar{\nu})\sqrt{B_o(\boldsymbol{p};\bar{\nu})}\, h_{vd}(\boldsymbol{x};\boldsymbol{p},\bar{\nu})\right] \tag{13}$$

The definition of the various terms in Eq.(13) is furnished below:

$$\mathcal{U}_{\text{ill}}(\boldsymbol{p};\bar{\nu}) \stackrel{\text{def}}{=} \left(\frac{\bar{\nu}}{ic}\right)\left[\int d\boldsymbol{s}\,\mathcal{A}(\boldsymbol{s};\bar{\nu})\frac{\exp\left(i2\pi\frac{\bar{\nu}}{c}[\|\boldsymbol{s}-\boldsymbol{p}\|]\right)}{\|\boldsymbol{s}-\boldsymbol{p}\|}\psi(\boldsymbol{s},\boldsymbol{p})\right]$$

complex-valued speckle illumination incident on the object point $\boldsymbol{p}$, due to scattering at the Virtual Source.

$$h_{vd}(\boldsymbol{x};\boldsymbol{p},\bar{\nu}) \stackrel{\text{def}}{=} \left(\frac{\bar{\nu}}{ic}\right)\left[\int d\boldsymbol{w}\left(\frac{\exp\left(i2\pi\frac{\bar{\nu}}{c}\|\boldsymbol{p}-\boldsymbol{w}\|\right)}{\|\boldsymbol{p}-\boldsymbol{w}\|}\psi(\boldsymbol{p},\boldsymbol{w})\right)\sqrt{R_{vd}(\boldsymbol{w};\bar{\nu})}\, h_{\text{blur}}(\boldsymbol{x};\boldsymbol{w},\bar{\nu})\right]$$

Speckle point-spread-function (PSF) of light transport from the obscured object point $\boldsymbol{p}$ to a point $\boldsymbol{x}$ on the sensor plane, following a scattering event at the Virtual Detector.

The reformulation helps draw parallels between conventional imaging using physical sources/detectors and indirect imaging using vitualized sources/detectors. The term $\mathcal{U}_{\text{ill}}(\boldsymbol{p};\bar{\nu})$ represents the incident illumination on the obscured object, while $h_{vd}(\boldsymbol{x};\boldsymbol{p},\bar{\nu})$ represents the PSF associated with light transport from the object to the sensor. The PSF $h_{vd}(\boldsymbol{x};\boldsymbol{p},\bar{\nu})$ represents the combined influence of scattering at the Virtual Detector relay wall and blurring intrinsic to optical imaging. As a result, the indirect imaging PSF $h_{vd}(\boldsymbol{x};\boldsymbol{p},\bar{\nu})$ is stochastic in character, and its structure depends on the unknown roughness profile of the Virtual Detector surface.

1.4 Expression for sampled hologram recorded by Lock-In sensor

At this point, we have the necessary ingredients to identify the expression for the sampled hologram recorded by the Lock-In sensor. It is obtained by incorporating the expression for the obscured object field $\mathcal{U}_o(\boldsymbol{x};\bar{\nu})$ identified in Eq.(13) into Eq.(11). The resulting expression is furnished in Eq.(14).

$$\mathcal{U}_\Delta[\boldsymbol{n};\bar{\nu}] = \frac{N}{\nu_b}\mathcal{U}_r^*[\boldsymbol{n};\bar{\nu}]\left\{\int_{(\boldsymbol{n}-0.5F)\Delta}^{(\boldsymbol{n}+0.5F)\Delta} d\boldsymbol{x}\,\mathcal{U}_o(\boldsymbol{x};\bar{\nu})\right\}$$

$$= \frac{N}{\nu_b}\mathcal{U}_r^*[\boldsymbol{n};\bar{\nu}]\left\{\int_{(\boldsymbol{n}-0.5F)\Delta}^{(\boldsymbol{n}+0.5F)\Delta} d\boldsymbol{x}\left(\int d\boldsymbol{w}\left(\int d\boldsymbol{p}\sqrt{B_o(\boldsymbol{p};\bar{\nu})}\left(\int d\boldsymbol{s}\,\mathcal{A}(\boldsymbol{s};\bar{\nu})\frac{\exp\left(i2\pi\frac{\bar{\nu}}{c}[\|\boldsymbol{s}-\boldsymbol{p}\|+\|\boldsymbol{p}-\boldsymbol{w}\|]\right)}{\|\boldsymbol{s}-\boldsymbol{p}\|\times\|\boldsymbol{p}-\boldsymbol{w}\|}\psi_{sp}\psi_{pw}\right)\right)\sqrt{R_{vd}(\boldsymbol{w};\bar{\nu})}\,h_{\text{blur}}(\boldsymbol{x};\boldsymbol{w},\bar{\nu})\right)\right\} \quad (14)$$

$$= \frac{N}{\nu_b}\mathcal{U}_r^*[\boldsymbol{n};\bar{\nu}]\left\{\int d\boldsymbol{p}\left(\mathcal{U}_{\text{ill}}(\boldsymbol{p};\bar{\nu})\sqrt{B_o(\boldsymbol{p};\bar{\nu})}\right)\left(\int_{(\boldsymbol{n}-0.5F)\Delta}^{(\boldsymbol{n}+0.5F)\Delta} d\boldsymbol{x}\,h_{vd}(\boldsymbol{x};\boldsymbol{p},\bar{\nu})\right)\right\} \quad (15)$$

The innermost nested integrals in Eq.(14) represent the sum of elementary phasors associated with each ray path originating at the Virtual Source, bouncing off the obscured object and terminating at the $\boldsymbol{n}^{th}$ Virtual Detector pixel. The spatial extent of the $\boldsymbol{n}^{th}$ Virtual Detector pixel is determined by the geometric image of the $\boldsymbol{n}^{th}$ physical detector pixel (IFOV).

The integral identity of Eq.(15) recasts the expression for the sampled hologram in a mathematical form that closely resembles the standard space-variant imaging formulation [7]. The term within blue brackets captures the effect of detector aliasing, and describes the number of speckle cells within the PSF $h_{vd}(\boldsymbol{x};\boldsymbol{p},\bar{\nu})$ that can be accommodated in a single detector pixel. This number should not be excessively large (< 10) to avoid amplitude/signal fading due to coherent averaging of speckle amplitudes within a pixel [4].

It is also evident from Eq.(15) that the optical hologram recorded by the physical detector does not exhibit any deterministic relationship to the obscured object field $\mathcal{U}_{\text{ill}}(\boldsymbol{p};\bar{\nu})\sqrt{B_o(\boldsymbol{p};\bar{\nu})}$. This is due in large part to the stochastic character of the PSF $h_{vd}(\boldsymbol{x};\boldsymbol{p},\bar{\nu})$, and the additional randomization imposed by integration of the PSF within each sensor pixel. Fortunately, it is possible under restricted conditions, to recover a holographic representation of the obscured object by computational mixing of the sampled hologram at $\bar{\nu}$ with a second hologram acquired at a closely spaced frequency $\bar{\nu}+\Delta\nu$. The remainder of this section identifies the conditions under which a latent hologram of the obscured object may be recovered.

We begin by identifying the expression for the sampled hologram at the second optical frequency $\bar{\nu}+\Delta\nu$, and is disclosed below:

$$\mathcal{U}_\Delta[\boldsymbol{n};\bar{\nu}+\Delta\nu] = \frac{N}{\nu_b}\mathcal{U}_r^*[\boldsymbol{n};\bar{\nu}+\Delta\nu]\left\{\int_{(\boldsymbol{n}-0.5F)\Delta}^{(\boldsymbol{n}+0.5F)\Delta} d\boldsymbol{x}\,\mathcal{U}_o(\boldsymbol{x};\bar{\nu}+\Delta\nu)\right\}$$

$$= \frac{N}{\nu_b}\mathcal{U}_r^*[\boldsymbol{n};\bar{\nu}+\Delta\nu]\left\{\int d\boldsymbol{p}\left(\mathcal{U}_{\text{ill}}(\boldsymbol{p};\bar{\nu}+\Delta\nu)\sqrt{B_o(\boldsymbol{p};\bar{\nu}+\Delta\nu)}\right)\left(\int_{(\boldsymbol{n}-0.5F)\Delta}^{(\boldsymbol{n}+0.5F)\Delta} d\boldsymbol{x}\,h_{vd}(\boldsymbol{x};\boldsymbol{p},\bar{\nu}+\Delta\nu)\right)\right\} \quad (16)$$

The definition of the various terms in Eq.(16) mirrors Eq.(13), and is restated for the benefit of the reader:

$$\mathcal{U}_{\text{ill}}(\boldsymbol{p};\bar{\nu}+\Delta\nu) \stackrel{\text{def}}{=} \left(\frac{\bar{\nu}+\Delta\nu}{ic}\right)\left(\int d\boldsymbol{s}\,\mathcal{A}(\boldsymbol{s};\bar{\nu}+\Delta\nu)\frac{\exp\left(i2\pi\frac{\bar{\nu}+\Delta\nu}{c}[\|\boldsymbol{s}-\boldsymbol{p}\|]\right)}{\|\boldsymbol{s}-\boldsymbol{p}\|}\psi(\boldsymbol{s},\boldsymbol{p})\right)$$

$$h_{vd}(\boldsymbol{x};\boldsymbol{p},\bar{\nu}+\Delta\nu) \stackrel{\text{def}}{=} \left(\frac{\bar{\nu}+\Delta\nu}{ic}\right)\left[\int d\boldsymbol{w}\left(\frac{\exp\left(i2\pi\frac{\bar{\nu}+\Delta\nu}{c}\|\boldsymbol{p}-\boldsymbol{w}\|\right)}{\|\boldsymbol{p}-\boldsymbol{w}\|}\psi(\boldsymbol{p},\boldsymbol{w})\right)\sqrt{R_{vd}(\boldsymbol{w};\bar{\nu}+\Delta\nu)}\,h_{\text{blur}}(\boldsymbol{x};\boldsymbol{w},\bar{\nu}+\Delta\nu)\right]$$

Examination of the expressions for the sampled holograms disclosed in Eqs.(15),(16) fails to divulge any obvious relation between the detected fields at the two closely spaced optical frequencies $\bar{\nu}$ and $\bar{\nu}+\Delta\nu$. The relation between the sampled holograms may be elucidated by making simplifying assumptions regarding the indirect imaging geometry and the scattering properties of the Virtual Source/Detector surfaces.

The simplifying assumptions are enumerated below:

A1. $\mathcal{A}(\boldsymbol{s};\bar{\nu}+\Delta\nu)=\mathcal{A}(\boldsymbol{s};\bar{\nu})$ — *Spectral reflectance of Virtual Source surface is unchanged for small change in optical frequency*

A2. $B_o(\boldsymbol{p};\bar{\nu}+\Delta\nu)=B_o(\boldsymbol{p};\bar{\nu})$ — *Object albedo is unchanged for small change in optical frequency*

A3. $R_{vd}(\boldsymbol{p};\bar{\nu}+\Delta\nu)=R_{vd}(\boldsymbol{p};\bar{\nu})$ — *Virtual Detector albedo is unchanged for small change in optical frequency*

A4. $\mathcal{U}_r[\boldsymbol{n};\bar{\nu}+\Delta\nu]=\mathcal{U}_r[\boldsymbol{n};\bar{\nu}]$ — *Phase fluctuations induced by a small change in the optical frequency of the reference beam are negligibly small*

A5. *The Virtual Source subtends a small solid angle with respect to the object,* so that a small change in the optical frequency of the CW source induces a proportional change in the optical path length (OPL) of ray paths $\overrightarrow{\boldsymbol{sp}}$ originating at a point $\boldsymbol{s}$ on the Virtual Source and terminating at the object point $\boldsymbol{p}$. The excess phase induced by the change in OPL is given below:

$$\Phi(\overrightarrow{\boldsymbol{sp}};\Delta\nu)\stackrel{\text{def}}{=}2\pi\left(\left(\frac{\bar{\nu}+\Delta\nu}{c}\right)\|\boldsymbol{s}-\boldsymbol{p}\|\right)-\left(\left(\frac{\bar{\nu}}{c}\right)\|\boldsymbol{s}-\boldsymbol{p}\|\right)=2\pi\left(\frac{\Delta\nu}{c}\right)\|\boldsymbol{s}-\boldsymbol{p}\|$$

$$\approx 2\pi\left(\frac{\Delta\nu}{c}\right)\|\bar{\boldsymbol{s}}-\boldsymbol{p}\|\left(1+\frac{1}{2}\frac{\|\boldsymbol{s}-\bar{\boldsymbol{s}}\|^2}{\|\bar{\boldsymbol{s}}-\boldsymbol{p}\|^2}+\frac{(\boldsymbol{s}-\bar{\boldsymbol{s}})^T(\bar{\boldsymbol{s}}-\boldsymbol{p})}{\|\bar{\boldsymbol{s}}-\boldsymbol{p}\|^2}\right) \tag{17}$$

$$\approx 2\pi\left(\frac{\Delta\nu}{c}\right)\|\bar{\boldsymbol{s}}-\boldsymbol{p}\|+\epsilon \tag{18}$$

where $\bar{\boldsymbol{s}}$ is a fixed point on the Virtual Source surface, such as the centroid of the VS. The assumption of small solid-angle is evidenced in the binomial approximation of Eq.(17).

The first term in Eq.(18) is the phase associated with a spherical wave at the notional wavelength (or synthetic wavelength) $c\Delta\nu^{-1}$ meters. It encodes the position of the obscured object from the vantage point of the Virtual Source. The second term ϵ in Eq.(18) is the excess phase or wavefront aberration induced by a change in the optical frequency. The term is dominated by the phase component $2\pi\left(\frac{\Delta\nu}{c}\right)(\boldsymbol{s}-\bar{\boldsymbol{s}})^T\left(\frac{\bar{\boldsymbol{s}}-\boldsymbol{p}}{\|\bar{\boldsymbol{s}}-\boldsymbol{p}\|}\right)$. The wavefront error imparted by this component is negligible so long as $|\epsilon|\ll 1$ radian [3], for each ray-path $\overrightarrow{\boldsymbol{sp}}$ originating at the Virtual Source and terminating at the obscured object point $\boldsymbol{p}$. As a result,

$$|\epsilon|\ll 1 \quad\Rightarrow\quad \left(\frac{\Delta\nu}{c}\right)\left|(\boldsymbol{s}-\bar{\boldsymbol{s}})^T\left(\frac{\bar{\boldsymbol{s}}-\boldsymbol{p}}{\|\bar{\boldsymbol{s}}-\boldsymbol{p}\|}\right)\right|\ll\frac{1}{2\pi} \tag{19}$$

$$\therefore\quad \|\boldsymbol{s}-\bar{\boldsymbol{s}}\|\left|\left(\frac{\boldsymbol{s}-\bar{\boldsymbol{s}}}{\|\boldsymbol{s}-\bar{\boldsymbol{s}}\|}\right)^T\left(\frac{\bar{\boldsymbol{s}}-\boldsymbol{p}}{\|\bar{\boldsymbol{s}}-\boldsymbol{p}\|}\right)\right|\ll\frac{c\Delta\nu^{-1}}{2\pi}\quad\Rightarrow\quad\|\boldsymbol{s}-\bar{\boldsymbol{s}}\|<\frac{c\Delta\nu^{-1}}{2\pi}$$

$$\|\boldsymbol{s}-\bar{\boldsymbol{s}}\|\approx\sqrt{\left(\frac{\emptyset_{vs}}{2}\right)^2+{\sigma_h}^2}\quad\text{so that}\quad\sqrt{\left(\frac{\emptyset_{vs}}{2}\right)^2+\sigma_h^2}<\frac{c\Delta\nu^{-1}}{8}<\frac{c\Delta\nu^{-1}}{2\pi} \tag{20}$$

where-in $\emptyset_{vs}$ reperesents the diameter of the illumination beam incident on the Virtual Source surface and σ_h represents the RMS roughness of the Virtual Source surface. The term $c\Delta\nu^{-1}$ represents the change in wavelength resulting from a change in the optical frequency of the CW source by $\Delta\nu$ Hz. In deriving Eq.(20), the wavenumber associated with the synthetic wavelength $2\pi(c^{-1}\Delta\nu)$ is approximated as $8(c^{-1}\Delta\nu)$ meter^{-1}.

The empirical bound of Eq.(20) divulges a tradeoff between the maximum permitted frequency shift $\Delta\nu$, and the choice of beam waist and RMS roughness of the Virtual Source surface. For a Virtual Source surface with approximate knowledge of the RMS roughness, a larger frequency shift may only be admitted by reducing the size of the Virtual Source, a fact borne out in experiments.

A6. *A small change $\Delta\nu$ in the optical frequency of the CW illumination source induces longitudinal chromatic aberration in the diffraction limited optical blur at $\bar{\nu}$*, and is expressed as follows:

$$h_{\text{blur}}\left(\boldsymbol{x} \stackrel{\text{def}}{=} \begin{bmatrix} x_1 \\ x_2 \end{bmatrix}; \boldsymbol{w} \stackrel{\text{def}}{=} \begin{bmatrix} w_1 \\ w_2 \\ w_3 \end{bmatrix}, \bar{\nu} + \Delta\nu\right) \approx \left(\frac{\bar{\nu} + \Delta\nu}{\bar{\nu}}\right)^2 \left[\exp\left(i\frac{2\pi\Delta\nu}{c}\left[z_d + \frac{[x_1^2 + x_2^2]}{2z_d} + \bar{w}_3 + \frac{[w_1^2 + w_2^2]}{2\bar{w}_3}\right]\right)\right] h_{\text{blur}}(\boldsymbol{x}; \boldsymbol{w}, \bar{\nu}) \tag{21}$$

The term $\bar{w}_3$ represents the mean distance from the VD surface to the entrance pupil plane of the imaging optic. The approximation is valid so long as the peak-valley height fluctuations in the Virtual Detector surface are smaller than $\frac{1}{8^{\text{th}}}$ change in the wavelength of the interrogation source, namely $c\Delta\nu^{-1}$ meters. A formal proof is furnished in Section-3.

- *Finite extent of each detector pixel:* Over the finite extent of the $\boldsymbol{n}^{th}$ detector pixel, the quadratic phase variation $\frac{2\pi\Delta\nu}{c}\frac{\|x\|^2}{2z_d}$ imparted by the longitudinal chromatic aberration in the optical blur of Eq.(21), can be approximated by the complex valued constant shown below:

$$\frac{2\pi\Delta\nu}{c}\frac{\|\boldsymbol{x}\|^2}{2z_d} \approx \frac{2\pi\Delta\nu}{c}\frac{\|\boldsymbol{n}\boldsymbol{\Delta}\|^2}{2z_d} \qquad \forall \boldsymbol{x} \in (\boldsymbol{n}\Delta - \mathbf{0.5}F\Delta, \boldsymbol{n}\Delta + \mathbf{0.5}F\Delta) \tag{22}$$

- *Finite extent of the optical blur:* The finite spatial extent of the optical blur h_{blur} limits the set of Virtual Detector locations $\boldsymbol{w}: (w_1, w_2, w_3)$ that contribute to the integrated field amplitude at the $\boldsymbol{n}^{th}$ detector pixel. These locations are confined to a small region in the vicinity of the geometric image of the center of the $\boldsymbol{n}^{th}$ detector pixel, as observed on the Vitual Detector surface. Thus, the position vector $[w_1, w_2, w_3]^T$ associated with a point on the VD surface may instead be approximated by the vector $[-z_d^{-1}\bar{w}_3\boldsymbol{n}\Delta \quad \bar{w}_3]^T$, for all $\boldsymbol{x} \in (\boldsymbol{n}\Delta - \mathbf{0.5}F\Delta, \boldsymbol{n}\Delta + \mathbf{0.5}F\Delta)$. The scalar $z_d^{-1}\bar{w}_3$ in the approximation represents the transverse magnification associated with the imaging identity of Eq.(1), and $\bar{w}_3$ is the mean perpendicular distance from the VD surface to the entrance pupil plane of the imaging optics. As a consequence of this approximation, the quadratic phase variations $\frac{2\pi\Delta\nu}{c}\frac{[w_1^2+w_2^2]}{2\bar{w}_3}$ imparted by the longitudinal chromatic aberration in the optical blur of Eq.(21), can be approximated by the complex valued constant shown below:

$$\frac{2\pi\Delta\nu}{c}\frac{[w_1^2 + w_2^2]}{2\bar{w}_3} \approx \frac{2\pi\Delta\nu}{c}\frac{\|z_d^{-1}\bar{w}_3\boldsymbol{n}\Delta\|^2}{2\bar{w}_3} \qquad \forall \boldsymbol{x} \in (\boldsymbol{n}\Delta - \mathbf{0.5}F\Delta, \boldsymbol{n}\Delta + \mathbf{0.5}F\Delta) \tag{23}$$

A7. *Each Virtual Detector pixel subtends a small solid angle with respect to the object,* so that change a small change in the optical frequency of the CW source induces a proportional change in the optical path length (OPL) of ray paths $\overrightarrow{\boldsymbol{pw}}$ originating at a point $\boldsymbol{p}$ on the obscured object and terminating at the point $\boldsymbol{w}$ on the Virtual Detector surface. The excess phase induced by the change in OPL is given :

$$\Phi(\overrightarrow{\boldsymbol{pw}}; \Delta\nu) \stackrel{\text{def}}{=} 2\pi\left(\left(\frac{\bar{\nu} + \Delta\nu}{c}\right)\|\boldsymbol{p} - \boldsymbol{w}\|\right) - \left(\left(\frac{\bar{\nu}}{c}\right)\|\boldsymbol{p} - \boldsymbol{w}\|\right) = 2\pi\left(\frac{\Delta\nu}{c}\right)\|\boldsymbol{p} - \boldsymbol{w}\|$$

$$\approx 2\pi\left(\frac{\Delta\nu}{c}\right)\left\|\boldsymbol{p} - \begin{bmatrix} -z_d^{-1}\bar{w}_3\boldsymbol{n}\Delta \\ \bar{w}_3 \end{bmatrix}\right\|\left(1 + \frac{1}{2}\frac{\left\|\begin{bmatrix} -z_d^{-1}\bar{w}_3\boldsymbol{n}\Delta \\ \bar{w}_3 \end{bmatrix} - \boldsymbol{w}\right\|^2}{\left\|\boldsymbol{p} - \begin{bmatrix} -z_d^{-1}\bar{w}_3\boldsymbol{n}\Delta \\ \bar{w}_3 \end{bmatrix}\right\|^2} + \frac{\left(\begin{bmatrix} -z_d^{-1}\bar{w}_3\boldsymbol{n}\Delta \\ \bar{w}_3 \end{bmatrix} - \boldsymbol{w}\right)^T\left(\boldsymbol{p} - \begin{bmatrix} -z_d^{-1}\bar{w}_3\boldsymbol{n}\Delta \\ \bar{w}_3 \end{bmatrix}\right)}{\left\|\boldsymbol{p} - \begin{bmatrix} -z_d^{-1}\bar{w}_3\boldsymbol{n}\Delta \\ \bar{w}_3 \end{bmatrix}\right\|^2}\right) \tag{24}$$

$$\approx 2\pi\left(\frac{\Delta\nu}{c}\right)\left\|\boldsymbol{p} - \begin{bmatrix} -z_d^{-1}\bar{w}_3\boldsymbol{n}\Delta \\ \bar{w}_3 \end{bmatrix}\right\| + \epsilon \tag{25}$$

where $\begin{bmatrix} -z_d^{-1}\bar{w}_3\boldsymbol{n}\Delta \\ \bar{w}_3 \end{bmatrix}$ is the geometric center of the $\boldsymbol{n}^{th}$ Virtual Detector pixel. The assumption of small solid-angle is evidenced in the binomial approximation of Eq.(24).

The first term in Eq.(25) is the phase associated with a spherical wave at the notional wavelength (or synthetic wavelength) $c\Delta\nu^{-1}$ meters. It encodes the position of the obscured object from the vantage point of the Virtual Detector, and is essential to recovering a holographic description of the obscured object. The second term ϵ in Eq. (25) is the excess phase or wavefront aberration induced by a change in the optical frequency. The term is dominated by the third component within blue brackets. The wavefront error imparted by this component is negligible so long as $|\epsilon| \ll 1$ radian [3], for each ray-path $\overrightarrow{\boldsymbol{pw}}$ originating at the obscured object point $\boldsymbol{p}$ and terminating in the $\boldsymbol{n}^{th}$Virtual Detector pixel. As a result,

$$|\epsilon| \ll 1 \quad \Rightarrow \quad \left(\frac{\Delta\nu}{c}\right)\left(\left(\begin{bmatrix} -z_d^{-1}\overline{w}_3\boldsymbol{n}\Delta \\ \overline{w}_3 \end{bmatrix} - \boldsymbol{w}\right)^T \left(\frac{\boldsymbol{p} - \begin{bmatrix} -z_d^{-1}\overline{w}_3\boldsymbol{n}\Delta \\ \overline{w}_3 \end{bmatrix}}{\left\|\boldsymbol{p} - \begin{bmatrix} -z_d^{-1}\overline{w}_3\boldsymbol{n}\Delta \\ \overline{w}_3 \end{bmatrix}\right\|}\right)\right) \ll \frac{1}{2\pi} \tag{26}$$

$$\therefore \quad \left\|\begin{bmatrix} -z_d^{-1}\overline{w}_3\boldsymbol{n}\Delta \\ \overline{w}_3 \end{bmatrix} - \boldsymbol{w}\right\| \left(\left(\frac{\begin{bmatrix} -z_d^{-1}\overline{w}_3\boldsymbol{n}\Delta \\ \overline{w}_3 \end{bmatrix} - \boldsymbol{w}}{\left\|\begin{bmatrix} -z_d^{-1}\overline{w}_3\boldsymbol{n}\Delta \\ \overline{w}_3 \end{bmatrix} - \boldsymbol{w}\right\|}\right)^T \left(\frac{\boldsymbol{p} - \begin{bmatrix} -z_d^{-1}\overline{w}_3\boldsymbol{n}\Delta \\ \overline{w}_3 \end{bmatrix}}{\left\|\boldsymbol{p} - \begin{bmatrix} -z_d^{-1}\overline{w}_3\boldsymbol{n}\Delta \\ \overline{w}_3 \end{bmatrix}\right\|}\right)\right) \ll \frac{c\Delta\nu^{-1}}{2\pi}$$

$$\Rightarrow \quad \left\|\begin{bmatrix} -z_d^{-1}\overline{w}_3\boldsymbol{n}\Delta \\ \overline{w}_3 \end{bmatrix} - \boldsymbol{w}\right\| < \frac{c\Delta\nu^{-1}}{2\pi}$$

$$\left\|\begin{bmatrix} -z_d^{-1}\overline{w}_3\boldsymbol{n}\Delta \\ \overline{w}_3 \end{bmatrix} - \boldsymbol{w}\right\| \approx \sqrt{\left(\frac{z_d^{-1}\overline{w}_3 F\Delta}{2}\right)^2 + \sigma_h^2} \quad \text{so that} \quad \sqrt{\left(\frac{z_d^{-1}\overline{w}_3 F\Delta}{2}\right)^2 + \sigma_h^2} < \frac{c\Delta\nu^{-1}}{8} \tag{27}$$

where-in $z_d^{-1}\overline{w}_3 F\Delta$ represents the active area of the $\boldsymbol{n}^{th}$ Virtual Detector pixel and and σ_h represents the RMS roughness of the Virtual Detector surface. The term $c\Delta\nu^{-1}$ represents the change in wavelength resulting from a change in the optical frequency of the CW source by $\Delta\nu$ Hz. In deriving Eq.(27), the wavenumber associated with the synthetic wavelength $2\pi(c^{-1}\Delta\nu)$ is approximated as $8(c^{-1}\Delta\nu)$ meter^{-1}.

The empirical bound of Eq.(27) divulges a tradeoff between the maximum permitted frequency shift $\Delta\nu$, and the size of a Virtual Detector pixel and RMS roughness of the Virtual Source surface. For a Virtual Detector surface with approximate knowledge of the RMS roughness, a larger frequency shift may only be admitted by reducing the size of a Virtual Detector pixel, a fact borne out in experiments.

The primary consequences of the simplifying assumptions of A1-A7 are enumerated below,

- *Field propagation from Virtual Source to obscured object:* A small change $\Delta\nu$ in the optical frequency of the CW source induces a proportional change in the OPL of ray paths $\overrightarrow{\boldsymbol{sp}}$ originating at a point $\boldsymbol{s}$ on the VS and terminating at the object point $\boldsymbol{p}$. The change in OPL can be approximated as $\|\overline{\boldsymbol{s}} - \boldsymbol{p}\|$, where $\overline{\boldsymbol{s}}$ is a fixed point such as the centroid of the illuminated region that makes up the Virtual Source.
- *Field propagation from obscured object to Virtual Detector:* A small change $\Delta\nu$ in the optical frequency of the CW source induces a proportional change in the OPL of ray paths $\overrightarrow{\boldsymbol{pw}}$ originating at a point $\boldsymbol{p}$ on the obscured object and terminating at the point $\boldsymbol{w}$ on the Virtual Detector surface. The change can be approximated as $\left\|\boldsymbol{p} - \begin{bmatrix} -z_d^{-1}\overline{w}_3\boldsymbol{n}\Delta \\ \overline{w}_3 \end{bmatrix}\right\|$, where $\begin{bmatrix} -z_d^{-1}\overline{w}_3\boldsymbol{n}\Delta \\ \overline{w}_3 \end{bmatrix}$ is the geometric center of the nth Virtual Detector pixel (geometric image of center of the nth detector pixel on the Virtual Detector surface).

- *Field propagation from Virtual Detector to image sensor:* A small change $\Delta\nu$ in the optical frequency of the CW source induces longitudinal chromatic aberration in the diffraction limited optical blur associated with imaging the Virtual Detector surface at $\bar{\nu}$. The phase fluctuations due to chromatic aberration may be approximated as $\frac{2\pi\Delta\nu}{c}\left(\frac{\|z_d^{-1}\bar{w}_3\boldsymbol{n}\Delta\|^2}{2\bar{w}_3}+\frac{\|\boldsymbol{n}\Delta\|^2}{2z_d}\right)$ where-in
    - $z_d^{-1}\bar{w}_3$ is the transverse magnification of the imager,
    - $\bar{w}_3$ is the mean perpendicular distance from the VD surface to the entrance pupil plane of the imaging optics,
    - z_d is the distance from the sensor plane to the exit pupil plane of the imaging optics.

1.5 Redundant information in the digital holograms at optical frequencies $\bar{\nu}$ and $\bar{\nu}+\Delta\nu$

A crucial step in assembling the hologram of the obscured objects is identifying redundant information in the complex-valued holograms recorded at the optical frequencies $\bar{\nu}$ and $\bar{\nu}+\Delta\nu$. To this end, we impose the restrictions A1-A7 upon the expressions for the indirect illumination $\mathcal{U}_{\text{ill}}$ and the indirect imager PSF h_{vd} . The expressions relating the indirect illumination at the two optical frequencies is disclosed below:

$$\mathcal{U}_{\text{ill}}(\boldsymbol{p};\bar{\nu}+\Delta\nu) \stackrel{\text{def}}{=} \left(\frac{\bar{\nu}+\Delta\nu}{ic}\right)\int d\boldsymbol{s}\,\mathcal{A}(\boldsymbol{s};\bar{\nu}+\Delta\nu)\frac{\exp\left(i2\pi\frac{\bar{\nu}+\Delta\nu}{c}[\|\boldsymbol{s}-\boldsymbol{p}\|]\right)}{\|\boldsymbol{s}-\boldsymbol{p}\|}\psi(\boldsymbol{s},\boldsymbol{p})$$
$$=\left(\frac{\bar{\nu}+\Delta\nu}{\bar{\nu}}\right)\times\exp\left(i\frac{2\pi\Delta\nu}{c}\|\bar{\boldsymbol{s}}-\boldsymbol{p}\|\right)\left\{\left(\frac{\bar{\nu}}{ic}\right)\int d\boldsymbol{s}\,\mathcal{A}(\boldsymbol{s};\bar{\nu})\frac{\exp\left(i2\pi\frac{\bar{\nu}}{c}[\|\boldsymbol{s}-\boldsymbol{p}\|]\right)}{\|\boldsymbol{s}-\boldsymbol{p}\|}\psi(\boldsymbol{s},\boldsymbol{p})\right\}$$
$$=\left(\frac{\bar{\nu}+\Delta\nu}{\bar{\nu}}\right)\times\exp\left(i\frac{2\pi\Delta\nu}{c}\|\bar{\boldsymbol{s}}-\boldsymbol{p}\|\right)\times\mathcal{U}_{\text{ill}}(\boldsymbol{p};\bar{\nu}) \tag{28}$$

It is evident from Eq.(28) that a small change in optical frequency of the CW source imparts a spherical phase to the field incident on the obscured object point $\boldsymbol{p}$. The excess phase depends on the propagation distance from the centroid of the Virtual Source to the obscured object point, and the incremental change in the wavelength of the CW source (given by $c\Delta\nu^{-1}$ meters). The phase encodes the position of the obscured object from the vantage point of the Virtual Source. (***Observation-1***)

In a similar fashion, it can be shown (Eq.(29)) that a small change in the optical frequency imparts additional spherical phase to the indirect imager PSF associated with each obscured object point $\boldsymbol{p}$.

$$h_{vd}(\boldsymbol{x};\boldsymbol{p},\bar{\nu}+\Delta\nu) \stackrel{\text{def}}{=} \left(\frac{\bar{\nu}+\Delta\nu}{ic}\right)\int d\boldsymbol{w}\left(\frac{\exp\left(i2\pi\frac{\bar{\nu}+\Delta\nu}{c}\|\boldsymbol{p}-\boldsymbol{w}\|\right)}{\|\boldsymbol{p}-\boldsymbol{w}\|}\psi(\boldsymbol{p},\boldsymbol{w})\right)\sqrt{R_{vd}(\boldsymbol{w};\bar{\nu}+\Delta\nu)}\,h_{\text{blur}}(\boldsymbol{x};\boldsymbol{w},\bar{\nu}+\Delta\nu)$$
$$=\left(\frac{\bar{\nu}+\Delta\nu}{\bar{\nu}}\right)^3\times\left\{\exp\left(i\frac{2\pi\Delta\nu}{c}\left[\left\|\boldsymbol{p}-\left[\frac{-z_d^{-1}\bar{w}_3\boldsymbol{n}\Delta}{\bar{w}_3}\right]\right\|+z_d+\frac{\|\boldsymbol{n}\Delta\|^2}{2z_d}+\bar{w}_3+\frac{\|z_d^{-1}\bar{w}_3\boldsymbol{n}\Delta\|^2}{2\bar{w}_3}\right]\right)\right\}$$
$$\times\left\{\int d\boldsymbol{w}\left(\frac{\exp\left(i2\pi\frac{\bar{\nu}}{c}\|\boldsymbol{p}-\boldsymbol{w}\|\right)}{\|\boldsymbol{p}-\boldsymbol{w}\|}\psi(\boldsymbol{p},\boldsymbol{w})\right)\sqrt{R_{vd}(\boldsymbol{w};\bar{\nu})}\,h_{\text{blur}}(\boldsymbol{x};\boldsymbol{w},\bar{\nu})\right\}$$
$$=\left(\frac{\bar{\nu}+\Delta\nu}{\bar{\nu}}\right)^3\times\left\{\exp\left(i\frac{2\pi\Delta\nu}{c}\left(\left\|\boldsymbol{p}-\left[\frac{-z_d^{-1}\bar{w}_3\boldsymbol{n}\Delta}{\bar{w}_3}\right]\right\|+z_d+\frac{\|\boldsymbol{n}\Delta\|^2}{2z_d}+\bar{w}_3+\frac{\|z_d^{-1}\bar{w}_3\boldsymbol{n}\Delta\|^2}{2\bar{w}_3}\right)\right)\right\}h_{vd}(\boldsymbol{x};\boldsymbol{p},\bar{\nu}) \tag{29}$$

The additional phase contribution highlighted in yellow varies with the propagation distance from the obscured object point $\boldsymbol{p}$ to the center of the $\boldsymbol{n}^{th}$ Virtual Detector pixel. The excess phase contribution highligted in blue varies with the length of the chief ray originating at the center of the $\boldsymbol{n}^{th}$ Virtual Detector pixel and terminating at the center of $\boldsymbol{n}^{th}$ sensor pixel.

A small change in the optical frequency of the CW source imparts a spherical phase to the impulse response of the indirect imager. The excess phase depends on the incremental change in the wavelength of the CW

source (given by $c\Delta\nu^{-1}$ meters), and the cumulative propagation distance from the obscured object point to the center of the $\boldsymbol{n}^{th}$ Virtual Detector and sensor pixels. (***Observation-2)***

Incorporating Eqs.(28)-(29) into Eq.(16) yields the following revised expression for the sampled hologram at the optical frequency $\bar{\nu}+\Delta\nu$:

$$\mathcal{U}_\Delta[\boldsymbol{n};\bar{\nu}+\Delta\nu] = \left(\frac{\bar{\nu}+\Delta\nu}{\bar{\nu}}\right)^4 \frac{N}{\nu_b}\mathcal{U}_r^*[\boldsymbol{n};\bar{\nu}]\exp(i\phi[\boldsymbol{n};\Delta\nu])\left\{\int d\boldsymbol{p}\left(\exp(i\theta[\boldsymbol{n};\boldsymbol{p},\Delta\nu])\left(\mathcal{U}_{\mathrm{ill}}(\boldsymbol{p};\bar{\nu})\sqrt{B_o(\boldsymbol{p};\bar{\nu})}\left(\int_{(\boldsymbol{n}-0.5F)\Delta}^{(\boldsymbol{n}+0.5F)\Delta} d\boldsymbol{x}\, h_{vd}(\boldsymbol{x};\boldsymbol{p},\bar{\nu})\right)\right)\right)\right\} \quad (30)$$

The definition of the phasors $\phi[\boldsymbol{n};\Delta\nu]$ and $\theta[\boldsymbol{n};\boldsymbol{p},\Delta\nu]$ is furnished below:

$$\phi[\boldsymbol{n};\Delta\nu] \stackrel{\text{def}}{=} 2\pi\frac{\Delta\nu}{c}\left(z_d + \frac{\|\boldsymbol{n}\Delta\|^2}{2z_d} + \bar{w}_3 + \frac{\|z_d^{-1}\bar{w}_3\boldsymbol{n}\Delta\|^2}{2\bar{w}_3}\right)$$

scene-independent phasor field representing the change in OPL associated with light transport from $\boldsymbol{n}^{th}$ Virtual Detector pixel to the $\boldsymbol{n}^{th}$ sensor pixel.

$$\theta[\boldsymbol{n};\boldsymbol{p},\Delta\nu] \stackrel{\text{def}}{=} 2\pi\frac{\Delta\nu}{c}\left(\|\bar{\boldsymbol{s}}-\boldsymbol{p}\| + \left\|\boldsymbol{p} - \left[\frac{-z_d^{-1}\bar{w}_3\boldsymbol{n}\Delta}{\bar{w}_3}\right]\right\|\right)$$

scene dependent phasor field representing the change in the OPL of all ray paths originating at the Virtual Source, bouncing off the obscured object and terminating at the $\boldsymbol{n}^{th}$ Virtual Detector pixel.

A small change $\Delta\nu$ in the optical frequency of the CW source used to interrogate the hidden scene imparts an additional spherical phase to the field contribution of each obscured object point $\boldsymbol{p}$. The excess spherical phase encodes the position of the obscured object point $\boldsymbol{p}$ at the "synthetic" wavelength $c\Delta\nu^{-1}$ meters. (***Observation-3***)

1.6 Recovering the latent hologram at the synthetic wavelength $c\Delta\nu^{-1}$

Observation-3 forms the basis of our claim (Claim-1) that a holographic description of the obscured object can be recovered despite scattering at the Virtual Source and Virtual Detector surfaces. As a first step towards corroborating this claim, we seek to computationally mix the sampled holograms at $\bar{\nu},\bar{\nu}+\Delta\nu$. The resulting expression is shown below:

$$\begin{aligned}\mathcal{U}_\Delta[\boldsymbol{n},\bar{\nu}]\times\mathcal{U}_\Delta^*[\boldsymbol{n},\bar{\nu}+\Delta\nu] &= \left(\left(\frac{N}{\nu_b}\right)^2\mathcal{U}_r[\boldsymbol{n};\bar{\nu}]\,\mathcal{U}_r^*[\boldsymbol{n};\bar{\nu}+\Delta\nu]\right)\times\left\{\left(\int_{(\boldsymbol{n}-0.5F)\Delta}^{(\boldsymbol{n}+0.5F)\Delta} d\boldsymbol{x}\,\mathcal{U}_o(\boldsymbol{x};\bar{\nu})\right)\times\left(\int_{(\boldsymbol{n}-0.5F)\Delta}^{(\boldsymbol{n}+0.5F)\Delta} d\acute{\boldsymbol{x}}\,\mathcal{U}_o^*(\acute{\boldsymbol{x}};\bar{\nu}+\Delta\nu)\right)\right\}\\ &= \left(\left(\frac{\bar{\nu}+\Delta\nu}{\bar{\nu}}\right)^4\left(\frac{N}{\nu_b}\right)^2 I_r[\boldsymbol{n};\bar{\nu}]\right)\exp(i\phi[\boldsymbol{n};\Delta\nu])\left\{\iint d\boldsymbol{p}d\acute{\boldsymbol{p}}\begin{pmatrix}\mathcal{U}_{\mathrm{ill}}(\boldsymbol{p};\bar{\nu})\mathcal{U}_{\mathrm{ill}}^*(\acute{\boldsymbol{p}};\bar{\nu})\\ \times\sqrt{B_o(\boldsymbol{p};\bar{\nu})B_o(\acute{\boldsymbol{p}};\bar{\nu})}\end{pmatrix}\exp(i\theta[\boldsymbol{n};\acute{\boldsymbol{p}},\Delta\nu])\left(\iint_{(\boldsymbol{n}-0.5F)\Delta}^{(\boldsymbol{n}+0.5F)\Delta} d\boldsymbol{x}d\acute{\boldsymbol{x}}\begin{pmatrix}h_{vd}(\boldsymbol{x};\boldsymbol{p},\bar{\nu})\\ \times\, h_{vd}^*(\acute{\boldsymbol{x}};\acute{\boldsymbol{p}},\bar{\nu})\end{pmatrix}\right)\right\}\end{aligned} \quad (31)$$

Interchanging the order of integration in Eq.(31) yields the following expression for the computational hologram:

$$\begin{aligned}\mathcal{U}_\Delta[\boldsymbol{n},\bar{\nu}]\times\mathcal{U}_\Delta^*[\boldsymbol{n},\bar{\nu}+\Delta\nu] &= \left(\left(\frac{\bar{\nu}+\Delta\nu}{\bar{\nu}}\right)^4\left(\frac{N}{\nu_b}\right)^2 I_r[\boldsymbol{n};\bar{\nu}]\right)\left(\begin{matrix}\exp(i\phi[\boldsymbol{n};\Delta\nu])\displaystyle\int d\boldsymbol{p}\left(|\mathcal{U}_{\mathrm{ill}}(\boldsymbol{p};\bar{\nu})|^2 B_o(\boldsymbol{p};\bar{\nu})\left|\int_{(\boldsymbol{n}-0.5F)\Delta}^{(\boldsymbol{n}+0.5F)\Delta} d\boldsymbol{x}\,h_{vd}(\boldsymbol{x};\boldsymbol{p},\bar{\nu})\right|^2\right)\exp(i\theta[\boldsymbol{n};p,\Delta\nu]) \;+\\ \exp(i\phi[\boldsymbol{n};\Delta\nu])\displaystyle\iint_{\boldsymbol{p}\neq\acute{\boldsymbol{p}}} d\boldsymbol{p}d\acute{\boldsymbol{p}}\begin{pmatrix}\mathcal{U}_{\mathrm{ill}}(\boldsymbol{p};\bar{\nu})\mathcal{U}_{\mathrm{ill}}^*(\acute{\boldsymbol{p}};\bar{\nu})\\ \times\sqrt{B_o(\boldsymbol{p};\bar{\nu})B_o(\acute{\boldsymbol{p}};\bar{\nu})}\end{pmatrix}\exp(i\theta[\boldsymbol{n};\acute{\boldsymbol{p}},\Delta\nu])\displaystyle\iint_{(\boldsymbol{n}-0.5F)\Delta}^{(\boldsymbol{n}+0.5F)\Delta} d\boldsymbol{x}d\acute{\boldsymbol{x}}\begin{pmatrix}h_{vd}(\boldsymbol{x};\boldsymbol{p},\bar{\nu})\\ \times\, h_{vd}^*(\acute{\boldsymbol{x}};\acute{\boldsymbol{p}},\bar{\nu})\end{pmatrix}\end{matrix}\right) \quad (32)\\ &= \mathcal{U}_{\mathrm{latent}}[\boldsymbol{n},\Delta\nu] + \mathcal{U}_{\mathrm{parasitic}}[\boldsymbol{n},\bar{\nu}] \quad (33)\end{aligned}$$

Notice that the expression for the computational hologram is comprised of two terms. The first of these terms higlighted in yellow encapsulates spectral intreference of field contributions for a single obscured object point $\boldsymbol{p}$. This term is dubbed the latent hologram as it encodes a holographic description of the obscured object, at the synthetic wavelngth of $\Lambda = c\Delta\nu^{-1}$ meters.

The second term highlighted in gray encapsulates the spectral interference of field contributions of two spatially distinct object points $\boldsymbol{p}, \acute{\boldsymbol{p}}$. This term is a parasitic interference term that complicates the recovery of the latent hologram.

The exact expression for the latent hologram may be identified by incorporating the definition of the scene-dependent and scene-independent phasor fields into the expression for the latent hologram disclosed in Eq(32). The resulting expression is furnished in Eq.(34).

$$\mathcal{U}_{\text{latent}}[\boldsymbol{n};\Delta\nu] = \left(\left(\frac{\bar{\nu}+\Delta\nu}{\bar{\nu}}\right)^4 \left(\frac{N}{\nu_b}\right)^2 I_r[\boldsymbol{n};\bar{\nu}]\right) \exp(i\phi[\boldsymbol{n};\Delta\nu]) \int d\boldsymbol{p} \left\{ \begin{array}{c} \left(|\mathcal{U}_{\text{ill}}(\boldsymbol{p};\bar{\nu})|^2 B_o(\boldsymbol{p};\bar{\nu}) \left| \int_{(\boldsymbol{n}-0.5F)\Delta}^{(\boldsymbol{n}+0.5F)\Delta} d\boldsymbol{x}\, h_{vd}(\boldsymbol{x};\boldsymbol{p},\bar{\nu}) \right|^2 \right) \\ \exp\left(i2\pi \frac{\Delta\nu}{c} \left(\|\bar{\boldsymbol{s}}-\mathbf{p}\| + \left\| \boldsymbol{p} - \begin{bmatrix} -z_d^{-1}\bar{w}_3 \boldsymbol{n}\Delta \\ \bar{w}_3 \end{bmatrix} \right\| \right) \right) \end{array} \right\} \tag{34}$$

The spherical phase factors embedded in Eq.(34) fully encode the position of the obscured object point $\boldsymbol{p}$ at the synthetic wavelength $c\Delta\nu^{-1}$ meters. Consequently, numerical backpropagation of the latent hologram should permit reconstruction of the light distribution in the hiddden volume. However, the ability to localize the obscured object point $\boldsymbol{p}$ is limited by the amplitude/strength of its contribution to the latent hologram, and determined by the following factors:

- Intensity of the indirect illumination contribution from the Virtual Source, given by $|\mathcal{U}_{\text{ill}}(\boldsymbol{p};\bar{\nu})|^2$.
- Albedo of the obscured object $B_o(\boldsymbol{p};\bar{\nu})$.
- Number of speckle cells in the indirect imager PSF $h_{vd}(\boldsymbol{x};\boldsymbol{p},\bar{\nu})$ that can be accommodated within a single detector pixel. This number should not be large (< 100) to avoid amplitude/signal fading due to coherent averaging of speckle amplitudes within a detector pixel [4].
- Radiometric fall-off due to propagation from the Virtual Source to the obscured object point $\boldsymbol{p}$ (encapsulated in the definition of $\mathcal{U}_{\text{ill}}(\boldsymbol{p};\bar{\nu})$), and propagation from the obscured object point $\boldsymbol{p}$ to the $\boldsymbol{n}^{th}$ Virtual Detector pixel (encapsulated in the definition of $h_{vd}(\boldsymbol{x};\boldsymbol{p},\bar{\nu})$).

In practice, the recovery of the latent hologram at the synthetic wavelength is complicated by the presence of the parasitic interference term in the computationally assembled hologram of Eq.(32). Insight into isolating the contributions of this term can be gleaned by examining the exact expression for the parasitic interference component, which is furnished below:

$$\mathcal{U}_{\text{parasitic}}[\boldsymbol{n};\Delta\nu] = \left(\left(\frac{\bar{\nu}+\Delta\nu}{\bar{\nu}}\right)^4 \left(\frac{N}{\nu_b}\right)^2 I_r[\boldsymbol{n};\bar{\nu}]\right) \exp(i\phi[\boldsymbol{n};\Delta\nu]) \iint_{\boldsymbol{p}\neq\acute{\boldsymbol{p}}} d\boldsymbol{p}\,d\acute{\boldsymbol{p}} \left\{ \begin{array}{c} \left(\begin{array}{c} \mathcal{U}_{\text{ill}}(\boldsymbol{p};\bar{\nu})\mathcal{U}_{\text{ill}}^*(\acute{\boldsymbol{p}};\bar{\nu}) \\ \times \sqrt{B_o(\boldsymbol{p};\bar{\nu})B_o(\acute{\boldsymbol{p}};\bar{\nu})} \end{array} \right) \left(\iint_{(\boldsymbol{n}-0.5F)\Delta}^{(\boldsymbol{n}+0.5F)\Delta} d\boldsymbol{x}\,d\acute{\boldsymbol{x}} \left(\begin{array}{c} h_{vd}(\boldsymbol{x};\boldsymbol{p},\bar{\nu}) \\ \times\ h_{vd}^*(\acute{\boldsymbol{x}};\acute{\boldsymbol{p}},\bar{\nu}) \end{array} \right) \right) \\ \exp\left(i\frac{\Delta\nu}{c} \left(\|\bar{\boldsymbol{s}}-\mathbf{p}\| + \left\| \boldsymbol{p} - \begin{bmatrix} -z_d^{-1}\bar{w}_3 \boldsymbol{n}\Delta \\ \bar{w}_3 \end{bmatrix} \right\| \right) \right) \end{array} \right\} \tag{35}$$

It is observed that the product of the indirect imager PSF's for distinct object positions exhibits spatial fluctuations at a scale determined by the numerical aperture of the imaging optics, which is far smaller than the synthetic wavelength. Consequently, the contributions of the parasitic interference term can be suppressed by low-pass filtering the computationally assembled hologram $\mathcal{U}_\Delta[\boldsymbol{n},\bar{\nu}] \times \mathcal{U}_\Delta^*[\boldsymbol{n},\bar{\nu}+\Delta\nu]$ with a cutoff frequency of $\frac{\Delta\nu}{c}$ cycles/meter. The exact same behavior may alternatively be realized by numerical backpropagation of the computational hologram at the synthetic wavelength $\Lambda = c\Delta\nu^{-1}$ meters.

Sufficient condition for avoiding wavefront aberration in latent hologram

The ability to recover a latent hologram of the obscured objects by computational mixing of optical holograms recorded at two closely spaced frequencies is predicated on the validity of approximations A5 and A7. Violation of the assumptions (Eqs.(20) and (27)) underlying these approximations induces wavefront aberrations in the latent hologram. Wavefront aberrations in the latent hologram may be avoided by summing up the constraints of Eqs.(20) and (27), namely:

Rayleigh criterion for wavefront reconstruction by computational mixing of scattered fields at two optical frequencies

$$\left(\sqrt{\left(\frac{\emptyset_{vs}}{2}\right)^2 + \sigma_h^2} + \sqrt{\left(\frac{z_d^{-1}\overline{w}_3 F\Delta}{2}\right)^2 + \sigma_h^2}\right) < \frac{c\Delta\nu^{-1}}{4} \qquad (36)$$

The term $\emptyset_{vs}$ represents the diameter of the beam incident on the Virtual Source surface, while $z_d^{-1}\overline{w}_3 F\Delta$ represents the physical extent of the active area of a Virtual Detector pixel. The term σ_h represents the RMS roughness of the Virtual Source/Detector surfaces. It is worth noting that the right hand side of Eq.(36) mirrors the Rayleigh quarter wave criterion for the synthetic wavelength, in what can only be described as a serendipitous confluence of constraints.

The inequality of Eq.(36) also divulges a complex tradeoff between the synthetic wavelength $\Lambda = c\Delta\nu^{-1}$ and the Indirect Imaging system parameters namely: the spatial extent of the Virtual Source, the size of a Virtual Detector pixel, and the RMS roughness σ_h of the Virtual Source/Detector surfaces. It is observed that smaller Virtual Source diameters, smaller Virtual Detector pixels and smoother Virtual Source/Detector surfaces permit the use of a smaller synthetic wavelength, a fact borne out in experiments.

Furthermore, the inequality of Eq.(36) may be recast to obtain a bound on the largest change in optical frequency $\Delta\nu$, and thereby the smallest synthetic wavelength $\Lambda = c\Delta\nu^{-1}$ that is free of wavefront aberration. The resulting expression is shown below:

$$\Lambda \stackrel{\text{def}}{=} c\Delta\nu^{-1} > 4\left(\sqrt{\left(\frac{\emptyset_{vs}}{2}\right)^2 + \sigma_h^2} + \sqrt{\left(\frac{z_d^{-1}\overline{w}_3 F\Delta}{2}\right)^2 + \sigma_h^2}\right) \qquad (37)$$

It is evident from the above discussion that computational mixing of scattered optical field recorded at two closely spaced frequencies $\nu_1 = \bar{\nu}, \nu_2 = (\bar{\nu} + \Delta\nu)$ preserves phase information at scales smaller than the difference frequency $\Delta\nu$, provided the maximum change in path length induced by a change in the optical frequency of interrrogation is smaller than the Rayleigh criterion $\frac{c\Delta\nu^{-1}}{4}$ meters. (***Observation-4***)

1.7 Resolution limits

In the absence of wavefront aberrations, the resolving power of the latent hologram is fundamentally limited by the synthetic wavelength $\Lambda = c\Delta\nu^{-1}$ meters, where $\Delta\nu$ is spacing between the optical frequencies used to interrogate the hidden volume. This limit stems from the inability to reproduce spatial detail exceeding $\Lambda^{-1} = c^{-1}\Delta\nu$ cycles/meter, when replaying the latent hologram. In practice, the achievable lateral resolution is further limited by the spatial extent of the Virtual Detector, and scales inversely with the propagation distance. The behavior is fully consistent with established limits in classical holography [8]. The expression for the lateral resolution at a nominal backpropagation distance of Z meters from the Virtual Detector surface is given by $\Lambda\left(\frac{Z}{M\times(z_d^{-1}\overline{w}_3\Delta)}\right)$. The product term $M\Delta$ represents the physical dimension of the image sensor, which is assumed to be square for simplicity. The product term $z_d^{-1}\overline{w}_3$ represents the transverse magnificaton of the optics used to image the Virtual Detector surface. Consequently, $M \times (z_d^{-1}\overline{w}_3\Delta)$ represents the spatial extent of the Virtual Detector.

Space-bandwidth product (SBP) of latent hologram

The finite spatial extent of the latent hologram (given by $M \times (z_d^{-1}\overline{w}_3\Delta)$ meters) combined with the finite spatial frequency bandwidth (given by $2\Lambda^{-1} = 2c^{-1}\Delta\nu$ cycles/meter) of the latent hologram, imposes a hard limit on the complexity of obscured objects that can be faithfully recorded and reproduced. Their product is a measure of the number of degrees of freedom of the latent hologram, and is referred to as the Space-Bandwidth Product (SBP) [3],[9] in optics litearture. The SBP of an optical signal is a measure of its information carrying capacity, and provides an upper bound on system performance. The SBP of the latent hologram is disclosed below:

$$SBP(\mathcal{U}_{\text{latent}}) \stackrel{\text{def}}{=} \left(\left(M \times (z_d^{-1}\overline{w}_3\Delta)\right)\right) \times (2\Lambda^{-1}) \tag{38}$$

The product term $M\Delta$ represents the physical dimension of the image sensor, which is assumed to be square for simplicity. The product term $z_d^{-1}\overline{w}_3$ represents the transverse magnificaton of the optics used to image the Virtual Detector surface. The expression for SBP disclosed in Eq.(38) , is strictly valid when the recovered latent hologram is devoid of any wavefront aberration at the synthetic wavelength. The expression for the smallest synthetic wavelength that is devoid of wavefront aberration was first disclosed in Eq.(37). sufficient condition for avoiding wavefront errors in the latent hologram. Incorporating the aformentioned result into the expression for the SBP, yields an upper-bound on the SBP of the latent hologram:

Upper-bound of SBP of Indirect Imaging using Synthetic Wavelength Holography

$$SBP \leq \frac{\left(M \times (z_d^{-1}\overline{w}_3\Delta)\right)}{2\left(\sqrt{\left(\frac{\emptyset_{vs}}{2}\right)^2 + \sigma_h^2} + \sqrt{\left(\frac{z_d^{-1}\overline{w}_3F\Delta}{2}\right)^2 + \sigma_h^2}\right)} \tag{39}$$

The term $\emptyset_{vs}$ represents the diameter of the beam incident on the Virtual Source surface, while $z_d^{-1}\overline{w}_3\Delta$ and $z_d^{-1}\overline{w}_3F\Delta$ represents the active area and pixel pitch of a Virtual Detector pixel, respectively. The term σ_h represents the RMS roughness of the Virtual Source/Detector surfaces. It is worth noting that the expression for SBP disclosed in Eq.(39), closely resembles Eq.(6) of the main manuscript. The term in the denominator may be viewed as a proxy for the peak-valley wavefront aberration at the synthetic wavelength Λ.

The upper bound on SBP for Synthetic Wavelength Holography disclosed in Eq.(39) is by no means the best that can be achieved from a theoretical standpoint. The asymptotic limit of the SBP for Indirect Imaging using Synthetic Wavelength Holography is obtained as $F \to 1$, $\emptyset_{vs} \to c\overline{\nu}^{-1}$ and $z_d^{-1}\overline{w}_3F\Delta \to 0.5c\overline{\nu}^{-1}$. These system parameters represent the absolute best that can achieved from the standpoint of sensor fill factor, spot diameter of the Virtual Source and recording fields with the highest resolution using classical optics. Under these restrictions, the RMS roughness of the intermediary surface $\sigma_h \gg \emptyset_{vs}, z_d^{-1}\overline{w}_3F\Delta$ so that the asymptotic SBP is given by:

$$SBP < (Mc\overline{\nu}^{-1})\left(\frac{1}{4\sigma_h}\right) \tag{40}$$

Consequently, the information carrying capacity of Synthetic Wavelength Holography is fundamentally limited by the roughness of the intermediary scattering surfaces that are adapted to serve as the Virtual Source/Detector. A comparison of the definition of the Space-Bandwdth product, disclosed in Eq(38), and the expression for the asymptotic SBP disclosed in Eq.(40), reveals a fundamental limit to the achievable resolution in Synthetic Wavelength Holography, and is given by $\Lambda > 8\sigma_h$.

Localization accuracy of the latent hologram

In the absence of wavefront aberrations, the longitudinal resolution of the latent hologram is limited to $2\Lambda\left(\frac{Z}{M\times(z_d^{-1}\bar{w}_3\Delta)}\right)^2$, and fully consistent with established limits in classical holography [8]. This limits our ability to precisely localize obscured objects within the hidden volume, and its impact is best illustrated in Figure-5 of the main manuscript. The traditional approach to tackling this problem is time-resolved holography using pulsed sources. Unfortunately, pulsed sources lack the temporal coherence needed to record holograms of objects obscured from view. The problem may be remedied by examining a computational approach to synthesizing light pulses using a multitude of optical frequencies, and is the topic of Section-2.

2. *Optical sectioning using a multitude of regularly spaced frequencies*

It is common knowledge that an ultrashort pulse train admits a Fourier series decomposition in the optical frequency domain. Herein we seek to computationally mimic the behavior by independently interrogating the hidden scene using a countably finite number of regularly spaced optical frequencies, and record the corresponding holograms. By computationally delaying the hologram recorded at each optical frequency, and accumulating the result across optical frequencies, it is possible to mimic interogation of the hidden scene by a pulse train. The process is illustrated in **Supplementary Figure3**, and is inspired by work in Fourier Synthesis Holography [10], and holographic laser radar [11],[12].

The periodicity of the pulse train is determined by the smallest separation between the optical frequencies, while the pulse duration is determined by the largest separation between the optical frequencies. It can be shown that K optical frequencies spaced apart by $\Delta\nu$ Hz can be used to computationally synthesize a periodic optical pulse train, with pulse duration $\tau_p = (K\Delta\nu)^{-1}$and repetition rate $\tau_{\text{rep}} = \Delta\nu^{-1}$ seconds. The shape of the pulse may be manipulated by appropriately weighting the holograms recorded at each optical frequency, prior to accumulation. In the simple case that the weights are chosen uniformly, the pulse envelope resembles a sinc-like function. By additionally delaying the hologram at each optical frequency, it is possible to synthesize a delayed pulse train that preferentially selects ray paths from the Virtual Source to the Virtual Detector with a prescribed round-trip distance. The notion is illustrated in the gary box designated "Computational path-length filtering" in **Supplementary Figure3**.

Unfortunately, the computationally filtered hologram $\mathcal{U}_{\text{fltrd}}[\boldsymbol{n}]$ is still plagued by speckle at the optical frequency. The problem is remedied by computationally mixing the filtered hologram with the hologram recorded at the optical frequency $\bar{\nu}$. The expression for the resulting latent hologram closely resembles the expression for the two frequency latent hologram disclosed in Eq.(34). The distinction emerges in the additional attenuation introduced by the path length filter $g\left(\|\bar{\boldsymbol{s}}-\boldsymbol{p}\| + \left\|\boldsymbol{p}-\begin{bmatrix}-z_d^{-1}\bar{w}_3\boldsymbol{n}\Delta \\ \bar{w}_3\end{bmatrix}\right\| - D\right)$. This filter promotes constructive interference of light at the synthetic wavelength for select propagation distances, and promotes destructive interference at remaining propagation distances.

The periodicity of the synthesized pulse train introduces periodic ambiguites in the response of the path length filter. This fact is corroborated in the experimental results of Figure-4 in the main manuscript.

The latent hologram assembled using the proposed approach is insensitive to scattering at the Virtual Source and Virtual Detector, features a lateral resolution limit of $c\left(\frac{(K-1)}{2}\Delta\nu\right)^{-1}$ meters and a longitudinal resolution of $2c(K\Delta\nu)^{-1}$ meters.

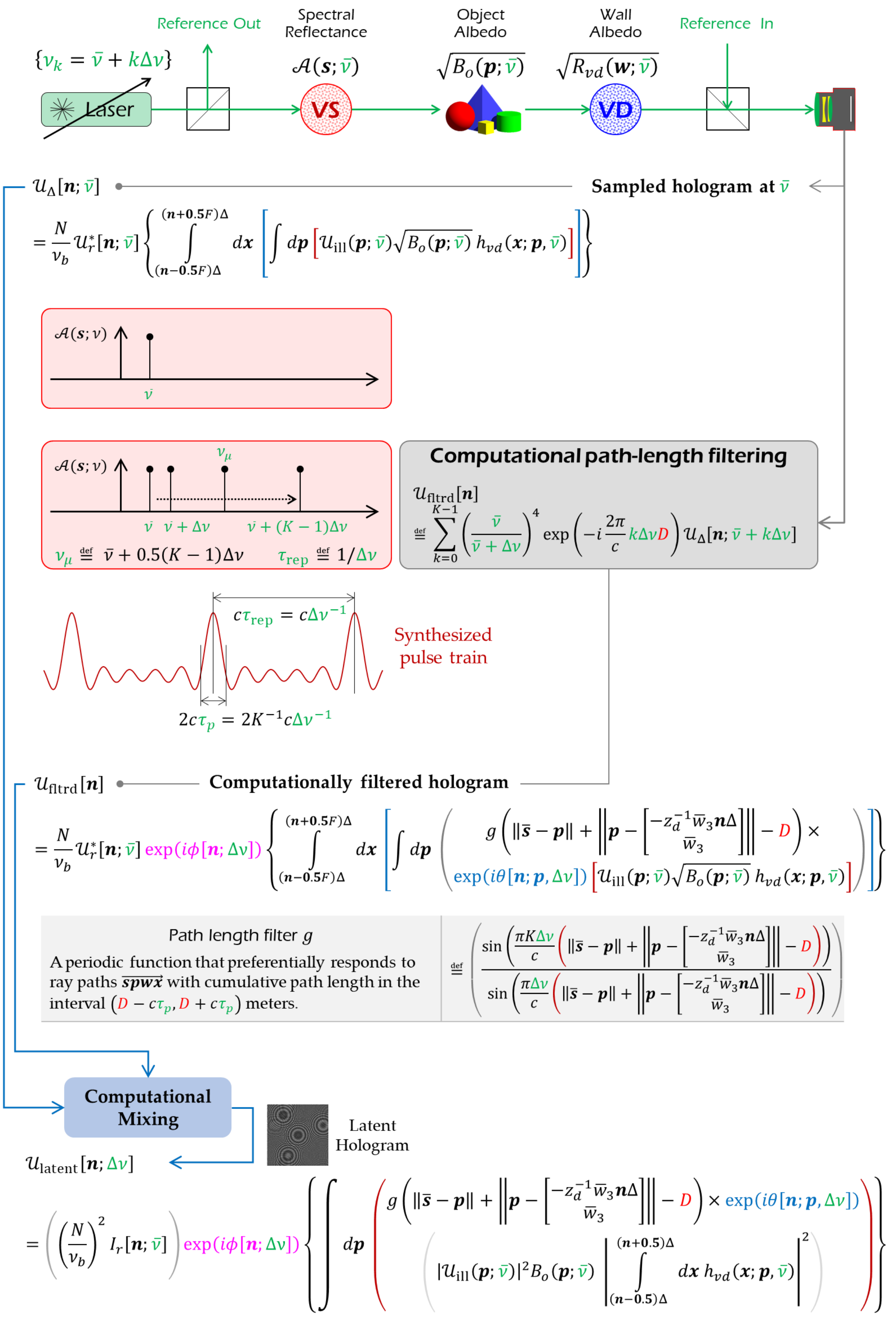

Supplementary Figure3: Optical sectioning using a multitude of regularly spaced frequencies

3. *Relating the optical blur at $\bar{\nu}+\Delta\nu$ to the optical blur at $\bar{\nu}$*

The optical blur associated with imaging the VD surface may be modeled as a paraxial blur [3] with image side numerical aperture Dz_d^{-1}, where D is the exit pupil diameter. The resulting amplitude PSF was originally disclosed in Eq.(2), and repeated below for the benefit of the reader.

$$h_{\text{blur}}\left(\boldsymbol{x} \stackrel{\text{def}}{=} \begin{bmatrix} x_1 \\ x_2 \end{bmatrix}; \boldsymbol{w} \stackrel{\text{def}}{=} \begin{bmatrix} w_1 \\ w_2 \\ w_3 \end{bmatrix}, \bar{\nu}\right) = \left(\begin{array}{c} \left(\frac{\bar{\nu}}{ic}\right)^2 \frac{1}{z_d w_3} \exp\left(i\frac{2\pi\bar{\nu}}{c}\left[z_d + w_3 + \frac{[x_1^2+x_2^2]}{2z_d} + \frac{[w_1^2+w_2^2]}{2w_3}\right]\right) \\ \int dudv \left[P(u,v)\exp\left(i\frac{2\pi\bar{\nu}}{c}\left[\frac{1}{z_d}+\frac{1}{w_3}-\frac{1}{f}\right](u^2+v^2)\right)\exp\left(-i\frac{2\pi\bar{\nu}}{c}\left(\left[\frac{x_1}{z_d}+\frac{w_1}{w_3}\right]u + \left[\frac{x_2}{z_d}+\frac{w_2}{w_3}\right]v\right)\right)\right] \end{array} \right) \tag{41}$$

The term $P(u,v)$ represents the transmittance function of the exit pupil and modeled as an indicator function with diameter D. The term f represents the focal length of the imaging optic. The depth w_3 of a point on the VD surface is measured with respect to the entrance pupil plane of the imaging optic.

The expression for the optical blur resulting from a small change in the optical frequency of the CW illumination source is disclosed below:

$$\begin{aligned} &h_{\text{blur}}\left(\boldsymbol{x} \stackrel{\text{def}}{=} \begin{bmatrix} x_1 \\ x_2 \end{bmatrix}; \boldsymbol{w} \stackrel{\text{def}}{=} \begin{bmatrix} w_1 \\ w_2 \\ w_3 \end{bmatrix}, \bar{\nu}+\Delta\nu\right) \\ &= \left(\begin{array}{c} \left(\frac{\bar{\nu}+\Delta\nu}{ic}\right)^2 \frac{1}{z_d w_3} \exp\left(i2\pi\frac{(\bar{\nu}+\Delta\nu)}{c}\left[z_d + w_3 + \frac{[x_1^2+x_2^2]}{2z_d} + \frac{[w_1^2+w_2^2]}{2w_3}\right]\right) \\ \int dudv \left[P(u,v)\exp\left(i2\pi\frac{(\bar{\nu}+\Delta\nu)}{c}\left[\frac{1}{z_d}+\frac{1}{w_3}-\frac{1}{f}\right](u^2+v^2)\right)\exp\left(-i2\pi\frac{(\bar{\nu}+\Delta\nu)}{c}\left(\left[\frac{x_1}{z_d}+\frac{w_1}{w_3}\right]u + \left[\frac{x_2}{z_d}+\frac{w_2}{w_3}\right]v\right)\right)\right] \end{array} \right) \\ &= \left(\begin{array}{c} \left(\begin{array}{c} \left\{\left(\frac{\bar{\nu}+\Delta\nu}{\bar{\nu}}\right)^2 \exp\left(i2\pi\frac{\Delta\nu}{c}\left[z_d + \frac{[x_1^2+x_2^2]}{2z_d} + w_3 + \frac{[w_1^2+w_2^2]}{2w_3}\right]\right)\right\} \times \\ \left\{\left(\frac{\bar{\nu}}{ic}\right)^2 \frac{1}{z_d w_3}\exp\left(i2\pi\frac{(\bar{\nu}+\Delta\nu)}{c}\left[z_d + w_3 + \frac{[x_1^2+x_2^2]}{2z_d} + \frac{[w_1^2+w_2^2]}{2w_3}\right]\right)\right\} \end{array} \right) \times \\ \left(\begin{array}{c} \left\{\int dudv \left[P(u,v)\exp\left(i2\pi\frac{\bar{\nu}}{c}\left[\frac{1}{z_d}+\frac{1}{w_3}-\frac{1}{f}\right](u^2+v^2)\right)\exp\left(-i2\pi\frac{\bar{\nu}}{c}\left(\left[\frac{x_1}{z_d}+\frac{w_1}{w_3}\right]u + \left[\frac{x_2}{z_d}+\frac{w_2}{w_3}\right]v\right)\right)\right]\right\} \otimes \\ \left\{\int dudv \left[\exp\left(i2\pi\frac{\Delta\nu}{c}\left[\frac{1}{z_d}+\frac{1}{w_3}-\frac{1}{f}\right](u^2+v^2)\right)\exp\left(-i2\pi\frac{\Delta\nu}{c}\left(\left[\frac{x_1}{z_d}+\frac{w_1}{w_3}\right]u + \left[\frac{x_2}{z_d}+\frac{w_2}{w_3}\right]v\right)\right)\right]\right\} \end{array} \right) \end{array} \right) \end{aligned} \tag{42}$$

The defocus aberration $\exp\left(i\frac{2\pi\Delta\nu}{c}\left[\frac{1}{z_d}+\frac{1}{w_3}-\frac{1}{f}\right](u^2+v^2)\right)$ induced by a small change in the optical frequency can be omitted so long as $\left[\frac{1}{z_d}+\frac{1}{w_3}-\frac{1}{f}\right]\frac{\Delta\nu}{c} \ll \frac{1}{2\pi}$. This implies that the defocus in waves $< \frac{1}{8^{\text{th}}}$ the synthetic wavelength $c\Delta\nu^{-1}$ meters. **(*Observation-5*)**

Incorporating the aforementioned constraint into Eq.(42) yields the revised expressions for optical blur disclosed in Eqs.(43)-(44).

$$\begin{aligned} &h_{\text{blur}}\left(\boldsymbol{x} \stackrel{\text{def}}{=} \begin{bmatrix} x_1 \\ x_2 \end{bmatrix}; \boldsymbol{w} \stackrel{\text{def}}{=} \begin{bmatrix} w_1 \\ w_2 \\ w_3 \end{bmatrix}, \bar{\nu}+\Delta\nu\right) \\ &= \left(\begin{array}{c} \left(\begin{array}{c} \left\{\left(\frac{\bar{\nu}+\Delta\nu}{\bar{\nu}}\right)^2 \exp\left(i2\pi\frac{\Delta\nu}{c}\left[z_d + \frac{[x_1^2+x_2^2]}{2z_d} + w_3 + \frac{[w_1^2+w_2^2]}{2w_3}\right]\right)\right\} \times \\ \left\{\left(\frac{\bar{\nu}}{ic}\right)^2 \frac{1}{z_d w_3}\exp\left(i2\pi\frac{(\bar{\nu}+\Delta\nu)}{c}\left[z_d + w_3 + \frac{[x_1^2+x_2^2]}{2z_d} + \frac{[w_1^2+w_2^2]}{2w_3}\right]\right)\right\} \end{array} \right) \times \\ \left(\begin{array}{c} \left\{\int dudv \left[P(u,v)\exp\left(i2\pi\frac{\bar{\nu}}{c}\left[\frac{1}{z_d}+\frac{1}{w_3}-\frac{1}{f}\right](u^2+v^2)\right)\exp\left(-i2\pi\frac{\bar{\nu}}{c}\left(\left[\frac{x_1}{z_d}+\frac{w_1}{w_3}\right]u + \left[\frac{x_2}{z_d}+\frac{w_2}{w_3}\right]v\right)\right)\right]\right\} \otimes \\ \left\{\int dudv \exp\left(-i2\pi\frac{\Delta\nu}{c}\left(\left[\frac{x_1}{z_d}+\frac{w_1}{w_3}\right]u + \left[\frac{x_2}{z_d}+\frac{w_2}{w_3}\right]v\right)\right)\right\} \end{array} \right) \end{array} \right) \end{aligned} \tag{43}$$

$$h_{\text{blur}}\left(\boldsymbol{x} \stackrel{\text{def}}{=} \begin{bmatrix} x_1 \\ x_2 \end{bmatrix}; \boldsymbol{w} \stackrel{\text{def}}{=} \begin{bmatrix} w_1 \\ w_2 \\ w_3 \end{bmatrix}, \bar{\nu} + \Delta\nu\right)$$

$$= \left(\begin{pmatrix} \left\{ \left(\frac{\bar{\nu}+\Delta\nu}{\bar{\nu}}\right)^2 \exp\left(i2\pi\frac{\Delta\nu}{c}\left[z_d + \frac{[x_1^2+x_2^2]}{2z_d} + w_3 + \frac{[w_1^2+w_2^2]}{2w_3}\right]\right)\right\} \times \\ \left\{ \left(\frac{\bar{\nu}}{ic}\right)^2 \frac{1}{z_d w_3} \exp\left(i2\pi\frac{(\bar{\nu}+\Delta\nu)}{c}\left[z_d + w_3 + \frac{[x_1^2+x_2^2]}{2z_d} + \frac{[w_1^2+w_2^2]}{2w_3}\right]\right)\right\} \end{pmatrix} \times \left(\begin{matrix} \left\{ \int dudv \left[P(u,v) \exp\left(i2\pi\frac{\bar{\nu}}{c}\left[\frac{1}{z_d} + \frac{1}{w_3} - \frac{1}{f}\right](u^2+v^2)\right) \exp\left(-i2\pi\frac{\bar{\nu}}{c}\left(\left[\frac{x_1}{z_d} + \frac{w_1}{w_3}\right]u + \left[\frac{x_2}{z_d} + \frac{w_2}{w_3}\right]v\right)\right)\right]\right\} \otimes \\ \left\{ \delta\left(\frac{\Delta\nu}{c}\left[\frac{x_1}{z_d} + \frac{w_1}{w_3}\right]u, \frac{\Delta\nu}{c}\left[\frac{x_2}{z_d} + \frac{w_2}{w_3}\right]v\right)\right\} \end{matrix} \right) \right) \quad (44)$$

The Dirac-delta function in Eq.(44) is a restatement of the imaging identity: $x_1 = -z_d \frac{w_1}{w_3}, x_2 = -z_d \frac{w_2}{w_3}$ first disclosed in Eq.(1), and is intrinsic to the process of imaging the Virtual Detector surface. The integral highlighted in blue yields a function of the form $g\left(\frac{\bar{\nu}}{c}\left(\left[\frac{x_1}{z_d} + \frac{w_1}{w_3}\right], \frac{\bar{\nu}}{c}\left[\frac{x_2}{z_d} + \frac{w_2}{w_3}\right]\right)\right)$, which when convolved with the the Dirac-delta function remains unchanged in functional form. This behavior can be attributed to the fact that the centroid of the optical blur spot $h_{\text{blur}}(\boldsymbol{x}; \boldsymbol{w}, \bar{\nu})$ also satifies the imaging identity. In view of this relation, the Dirac-delta function can be ignored from subsequent analysis. Consequently,

$$h_{\text{blur}}\left(\boldsymbol{x} \stackrel{\text{def}}{=} \begin{bmatrix} x_1 \\ x_2 \end{bmatrix}; \boldsymbol{w} \stackrel{\text{def}}{=} \begin{bmatrix} w_1 \\ w_2 \\ w_3 \end{bmatrix}, \bar{\nu} + \Delta\nu\right)$$
$$= \left(\frac{\bar{\nu}+\Delta\nu}{\bar{\nu}}\right)^2 \left[\exp\left(i2\pi\frac{\Delta\nu}{c}\left[z_d + \frac{[x_1^2+x_2^2]}{2z_d} + w_3 + \frac{[w_1^2+w_2^2]}{2w_3}\right]\right)\right] h_{\text{blur}}\left(\boldsymbol{x} \stackrel{\text{def}}{=} \begin{bmatrix} x_1 \\ x_2 \end{bmatrix}; \boldsymbol{w} \stackrel{\text{def}}{=} \begin{bmatrix} w_1 \\ w_2 \\ w_3 \end{bmatrix}, \bar{\nu}\right) \quad (45)$$

Further simplification is possible if it is assumed that the RMS roughness of the VD surface is much smaller than the mean depth of the Virtual Detector surface, so that

$$\frac{1}{w_3} \approx \frac{1}{\bar{w}_3} - \left(\frac{\delta w_3}{\bar{w}_3^2}\right) \quad (46)$$

where the depth w_3 of a point on the Virtual Detector surface is assumed to be a 2D random variable with mean value $\bar{w}_3$ and a zero-mean stochastic term δw_3 representing the surface height fluctuations. If additionally the peak height fluctuation in the VD surface $\frac{\delta w_3}{\bar{w}_3} < \frac{c(\Delta\nu)^{-1}}{8} \bar{w}_3$, then the linear phase fluctuation $\frac{2\pi\Delta\nu}{c}\frac{\delta w_3}{\bar{w}_3^2}$ induced by the optical roughness of the Virtual Detector surface may be omitted from further consideration. Incorporating the above into Eqs.(45)-(46) yields the following simplified relation between the optical blurs at $\bar{\nu}, \bar{\nu} + \Delta\nu$:

$$h_{\text{blur}}\left(\boldsymbol{x} \stackrel{\text{def}}{=} \begin{bmatrix} x_1 \\ x_2 \end{bmatrix}; \boldsymbol{w} \stackrel{\text{def}}{=} \begin{bmatrix} w_1 \\ w_2 \\ w_3 \end{bmatrix}, \bar{\nu} + \Delta\nu\right)$$
$$= \left(\frac{\bar{\nu}+\Delta\nu}{\bar{\nu}}\right)^2 \left[\exp\left(i2\pi\frac{\Delta\nu}{c}\left[z_d + \frac{[x_1^2+x_2^2]}{2z_d} + \bar{w}_3 + \frac{[w_1^2+w_2^2]}{2\bar{w}_3}\right]\right)\right] h_{\text{blur}}\left(\boldsymbol{x} \stackrel{\text{def}}{=} \begin{bmatrix} x_1 \\ x_2 \end{bmatrix}; \boldsymbol{w} \stackrel{\text{def}}{=} \begin{bmatrix} w_1 \\ w_2 \\ w_3 \end{bmatrix}, \bar{\nu}\right) \quad (47)$$

This concludes the proof.

4. *Experimental setups for NLoS Imaging using SWH*

Supplementary Figure 4 shows images and schematics of experimental setups used to demonstrate the ability to recover synthetic holograms of objects obscured from view. Details of the setups and interferometer architectures are described in the methods section of the main manuscript.

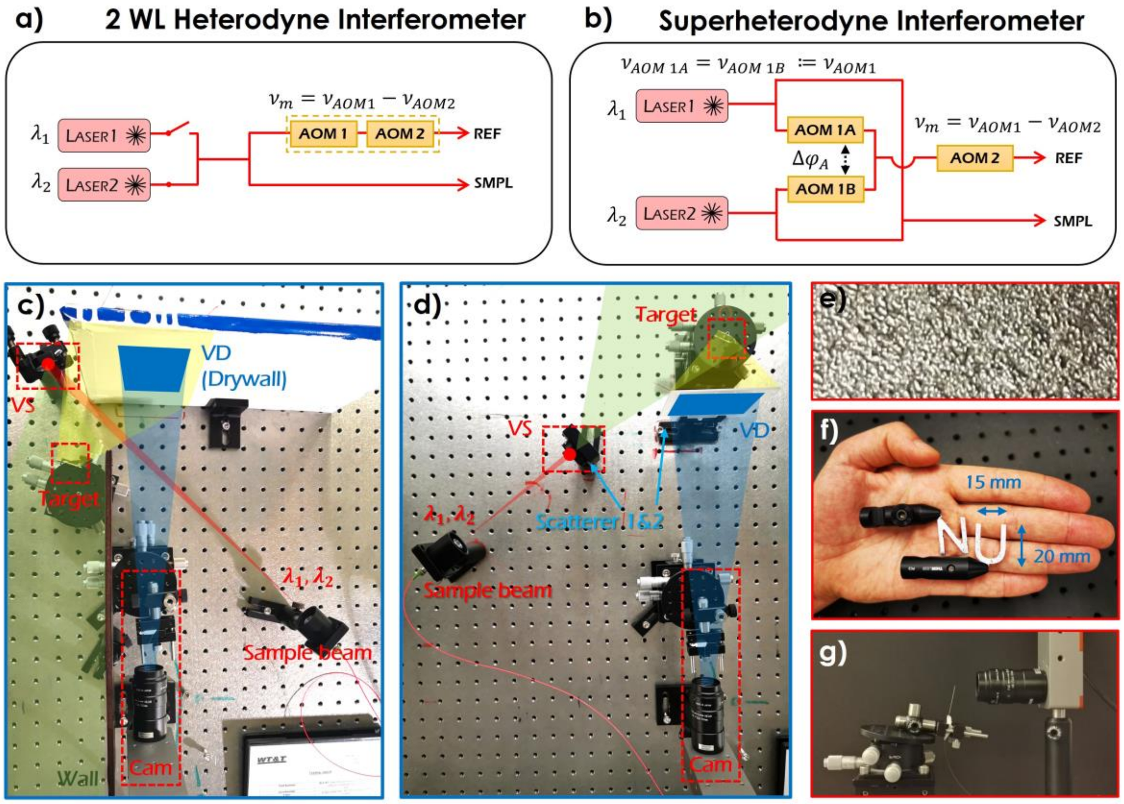

Supplementary Figure 4: Images and schematics of experimental setups used to validate NLoS Imaging using SWH. a) and b) Interferometer designs used to capture the 'Synthetic Wavelength Hologram' (SWH). Both interferometers introduce a small frequency shift of several kHz between sample and reference arm, used to demodulate the signal at the SWL. a) Dual Wavelength Heterodyne interferometer. b) Superheterodyne interferometer. c) Experimental layout for NLoS imaging in the 'imaging around corners' configuration. d) Experimental layout for NLoS imaging in the 'imaging through scattering media' configuration. e) Closeup image of the rough target surface and the Virtual Source (*VS*) surface used in experiments: sandblasted metal coated with silver. f) Image of the targets used in experiments: two characters 'N' and 'U 'with dimensions ~15mm×20mm (plus black optical mounts). g) Low insertion loss apparatus for injecting reference beam using a 'lensed fiber needle'.

5. *Comparison of Synthetic Wavelength Holography and Indirect Imaging Correlography*

There are two distinct approaches to exploiting corelations in scattered light for the purpose of NLoS imaging. These include:

- Synthetic Wavelength Holography (SWH), which exploits spectral correlations in scattered light, and
- Indirect Imaging Correlography (IIC) [15-17], which explots angular correlations in scattered light.

The difference in the correlation mechanism exploited by each of these NLoS imaging schemes imposes distinct limitations on their performance. The resolving power of SWH is limited by the spectral correlation bandwidth ('spectral memory effect'), while the angular Field of View (FoV) of Correlography is limited by the isoplanatic angle of the Virtual Detector relay wall (angular bandwidth of memory effect). The aforementioned angular-FoV versus angular-resolution tradeoff is reminiscent of the angular Space-Bandwidth tradeoff in classical imaging [15]. In an effort to clarify the implications of this FoV-Resolution tradeoff, we examine the NLoS imaging experiment of **Supplementary Figure 5**. The objective is to discern four US quarters obscured from direct view. The angular subtends of each US quarter with respect to the Virtual Detector relay wall is within the isoplanatic angle of the relay wall. However, the combined angular subtends of the coin ensemble significantly exceeds the isoplanatic angle (nearly 30°) of the relay wall. Subsequent paragraphs describe our attempts to recover hidden scene information using Indirect Imaging Correlography [15-17] and Synthetic Wavelength Holography.

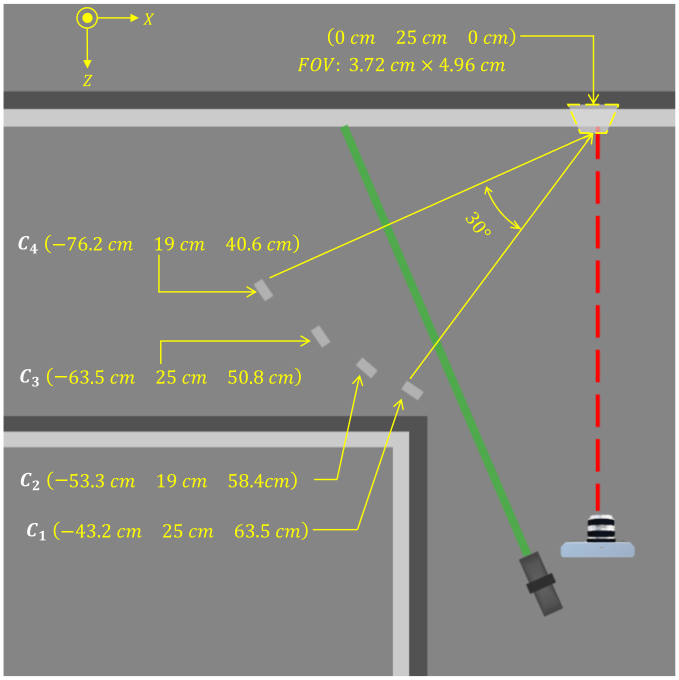

Supplementary Figure 5: A comparison of Non-Line-of-Sight using Synthetic Wavelength Holography and Indirect Imaging Correlography

Light from a tunable laser (Toptica SHG Pro, $560\ nm\ - 562\ nm$) focused on the Virtual Source surface serves to indirectly illuminate the coins obscured from view. An imager (Sigma 135mm f/1.8 DG HSM Art Lens + IDS UI-3272SE-M image sensor) observering the Virtual Detector surface (with a probe area of $37.2\ mm \times 49.6\ mm$) records the interference of the light scattered by the obscured object with a reference beam (not shown in the illustration). The recorded interferograms are processed using the scheme described in [18], to recover a hologram of the light scattered by the obscured coins. The squared magnitude of the speckle hologram yeilds an intensity image of the light scattered by the obscured coins and intercepred by the Virtual Detector. This image serves as input to Indirect Imaging Correlography [15-17]. The above process is repeated for different Virtual Source positions. The ensemble averaged power-spectrum of the intensity images may be used to recover an estimate of the auto-correlation of the obscured object albedo, as illustrated in **Supplementary Figure 6**. In the present example, the object albedo is

comprised of four blobs so that the auto-correlation is comprised of seven blobs. It is evident from **Supplementary Figure 7B** that the estimated auto-correlation bears no resemblance to seven blobs. The discrepancy arises from the fact that angular subtends of the object ensemble exceeds the isoplanatic angle of the Virtual Detector relay wall.

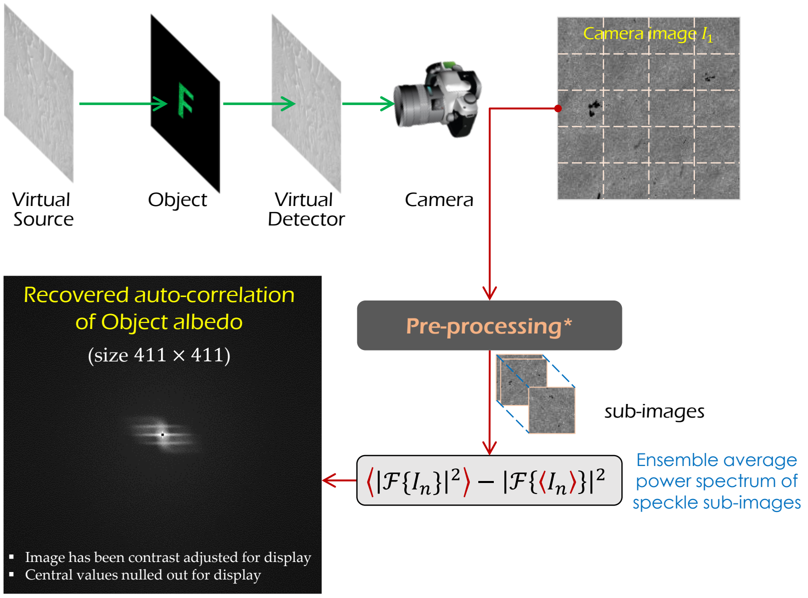

Supplementary Figure 6: Indirect Imaging Correlography (IIC) workflow outlined in [15-17]

By exploiting spectral correlations in scattered light, Synthetic Wavelength Holography is able to bypass restrictions imposed by the limited isoplanatic angle of the relay wall. To highlight this fact, we record a hologram of the light scattered by the obscured coins and intercepred by the Virtual Detector, at a second wavelength that is different from the first wavelength. By computationally mixing the two holograms, as described in the main manuscript, it is posible to assemble a synthetic holgram of the obscured coins.

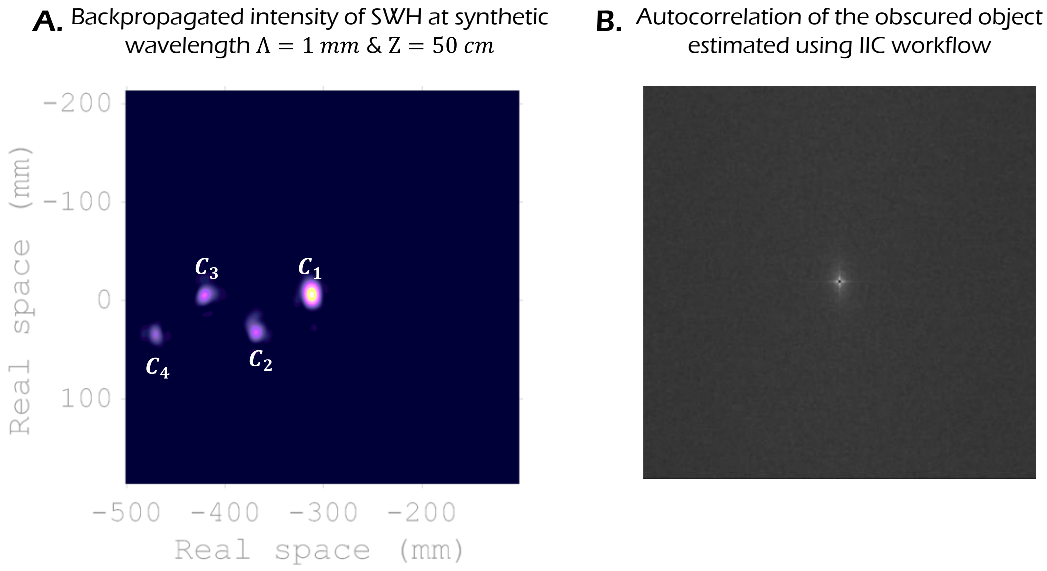

Supplementary Figure 7: Comparison of reconstructed images in a) SWH and b) IIC.

The phase and the Fourier modulus of the synthetic hologram observed at $\Lambda = 1$ mm is illustrated in **Supplementary Figure 8**. It is evident that SWH is able to clearly distinguish the four coins, unlike Correlography. The spatial coordinates of the four peaks visible in the Fourier modulus represent the direction of arrival of light from each obscured coin. Numerical back-propagation of the synthetic hologram to the plane of sharp focus ($Z = 50cm$) yields an image of the four coins, as illustrated in **Supplementary Figure 7A**.

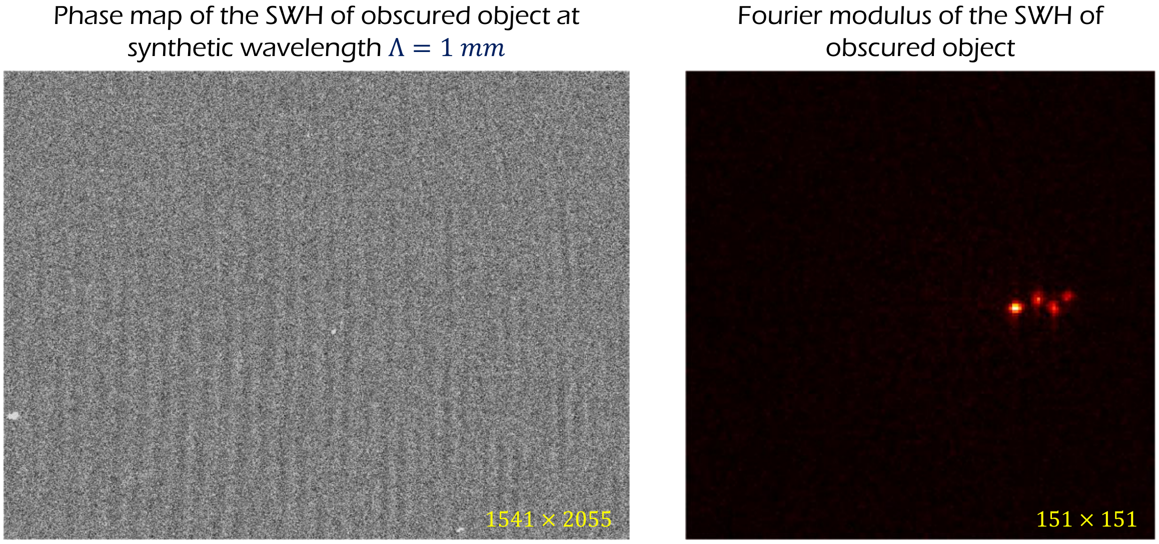

Supplementary Figure 8: Discerning four coins using Synthetic Wavelength Holography

The second experiment described herein highlights the hemispherical Field-of-View capability of Synthetic Wavelength Holography. The hidden scene is comprised of five US Quarters arranged as illustrated in **Supplementary Figure 9**. Three of the coins are fully obscured from direct view, while two are partially obscured from direct view. The angular extent of the coin ensemble exceeds 50°. A synthetic hologram of the obscured coins is assembled using the experimental procedure outlined in the previous example. Numerical back-propagation of the synthetic hologram to the plane of sharp focus ($Z = 50\ cm$) yields an image of the four coins, as evidenced in **Supplementary Figure 9**. The difference in the visiblity of the coins stems from radiometric losses.

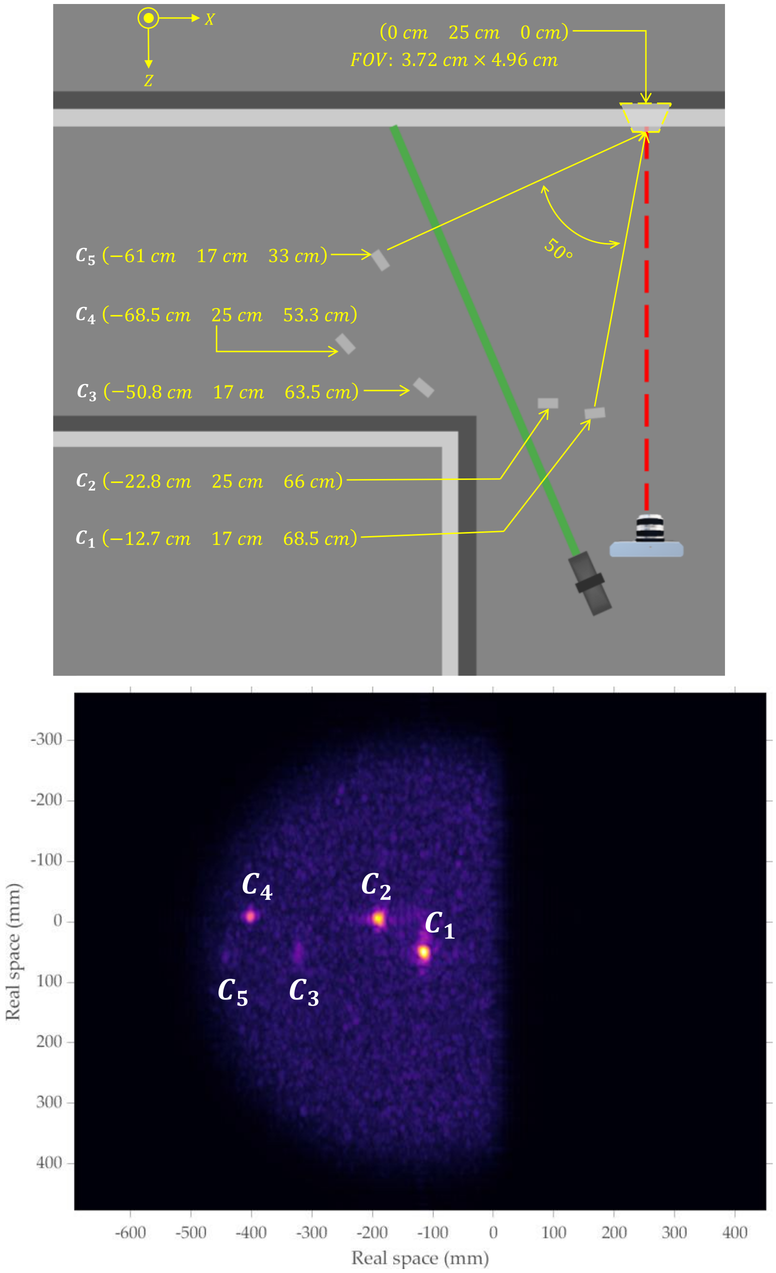

Supplementary Figure 9: Demonstration of the Wide Field of View capability of SWH

6. *Characterizing the severity of scatter using speckle correlations*

There are numerous approaches to estimating the transport properties of elastic scattering in the weak scattering limit (mean free path MFP far greater than wavelength). For samples (such as thin diffusers) whose physical thickness is much smaller than the Transport MFP (TMFP), it is common practice to estimate the MFP using Beer Lambert's law. For thicker samples (such as slabs) whose physical thickness exceeds multiple Transport Mean Free Paths, the TMFP is inferred by fitting the observed temporal response (Time-of-flight distribution) of the scattering medium to the response predicted by the diffusion equation. For samples such as the plastic plate used in our experiment, the ballistic contributions cannot be estimated reliably, limiting its use in estimating the MFP. Attempts to identify the temporal response of the

plastic plate require detectors with sub-picosecond resolution, presenting a challenge. We circumvent both problems, by relying on coherent approaches to estimating the transport properties of a scattering medium [1,19-22]. These techniques examine intensity fluctuations in the speckle field emerging from a scattering medium, as the frequency of the laser emission is varied. It is observed that the spectral correlation function disclosed below, provides all information pertinent to photon migration within the scattering medium.

$$C(\Delta\nu) = \frac{\langle\, \delta I(\bar{\nu})\, \delta I(\bar{\nu} + \Delta\nu)\,\rangle}{\langle\, \sigma(\bar{\nu})\,\rangle \langle\, \sigma(\bar{\nu} + \Delta\nu)\,\rangle} \quad (48)$$

The quantity $\Delta\nu = (\bar{\lambda})^{-1} - (\bar{\lambda} + \Delta\lambda)^{-1}$ represents the frequency difference in laser emission, as expressed in wavenumber units cm^{-1} [22]. The brackets $\langle\rangle$ in the above equation denote ensemble averaging over different realizations of disorder and is oftentimes replaced with spatial averaging for ergodic scattering processes. The term $\delta I(\bar{\nu}) = I(\bar{\nu}) - \langle I(\bar{\nu})\rangle$ denotes fluctuations in the intensity about the mean value, while $\sigma^2(\bar{\nu}) = \langle I^2(\bar{\nu})\rangle - \langle I(\bar{\nu})\rangle^2$ represents the variance of the intensity fluctuations. Qualitatively, one expects the intensity of a speckle cell to transition from bright to dark when the change in frequency $\Delta\nu_{opt}$ satisfies $C(\Delta\nu_{opt}) = 0.5$. The associated wavelength given by $\Lambda_{opt} = \Delta\nu_{opt}^{-1}$ represents the smallest synthetic wavelength for which the reconstructed synthetic hologram is devoid of speckle artifacts. For synthetic wavelengths smaller than Λ_{opt}, we should expect to observe increased speckle artifacts in the reconstructed synthetic hologram.

The apparatus of **Supplementary Figure 10** is used to characterize the severity of scatter within the plastic plate and the ground glass diffuser. Narrowband laser light (Toptica DFB pro 855nm) emerging from a single mode fiber is used to flood illuminate the proximal end of the scattering sample. A CMOS image sensor positioned at $850mm$ from the distal end of the scattering sample is used to record the objective speckle pattern emerging from the scattering sample. The standoff distance was chosen to ensure that the digitzed speckle recorded are spatially resolved. The wavelength of laser emission is controlled by tuning the temperature of the DFB laser diode, using the Toptica DLC Pro controller.

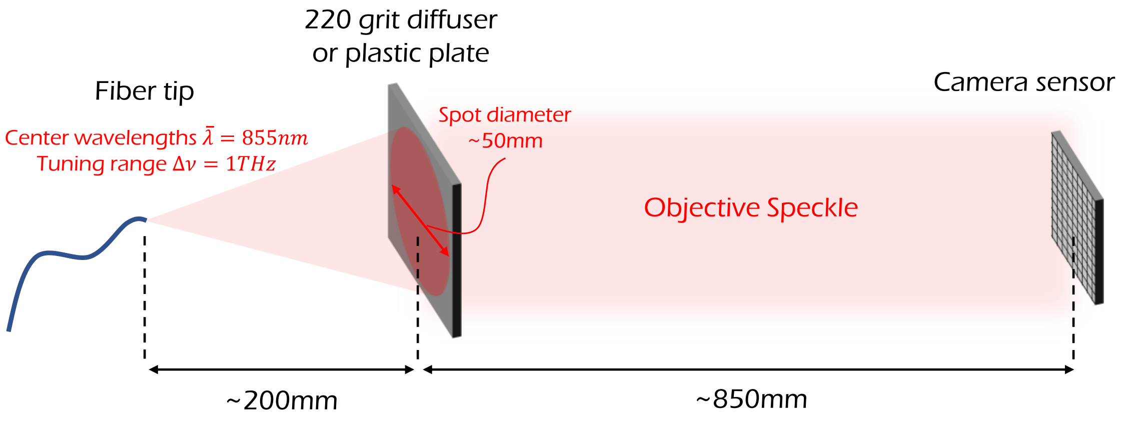

Supplementary Figure 10: Apparatus for characterizing transport properties of scattering samples

A series of speckle intensity images are recorded as the wavelength of emission is tuned over a spectral range of $\approx 1\,THz$, centered at $\bar{\lambda} = 855nm$. It was observed that the laser power varies as the emission wavelength is tuned over $1\,THz$. The variation is digitally compensated by processing the acquired images so that their histograms are identical. The image recorded at each wavelength is sub-divided into overlapping regions/sub-images of size 251×251 pixels. The process yields about 270 sub-images for each wavelength. The spectral correlation function $C(\Delta\nu)$ is estimated as the ensemble average ($\langle\rangle$ operator in Eq.(48) of the normalized cross-correlation between a sub-image at the reference wavelength $\bar{\lambda}$ and the

same sub-image at a second wavelength $\bar{\lambda} + \Delta\lambda$. The use of normalized cross-correlation accommodates any uncompensated changes in brightness and contrast between the sub-images at the two wavelengths.

The above procedure was used to obtain a plot of the spectral correlation function $C(\Delta\nu)$ for the scattering samples (diffuser and plastic plate) examined in the main manuscript. The result is furnished in **Supplementary Figure 11** and **Supplementary Figure 12**. The Full-Width Half Maximum (FWHM) of the spectral correlation function for each of the materials provides an estimate of the smallest synthetic wavelength for which the reconstruction is devoid of speckle artifacts (see Figure-4 of main manuscript). It is observed that the smallest synthetic wavelength for the plastic plate is $\Lambda_{opt} = 400\mu m$ $(\Delta\nu_{opt} = 25cm^{-1})$, and $\Lambda_{opt} = 300\mu m$ $\left(\Delta\nu_{opt} = 33.33cm^{-1}\right)$ for the 220-grit diffuser. This is in accordance to our experiments schown in Figure-4 of the main manuscript, where we observe increased synthetic speckle artifacts in the reconstructed synthetic hologram for employed synthetic wavelengths smaller than Λ_{opt}.

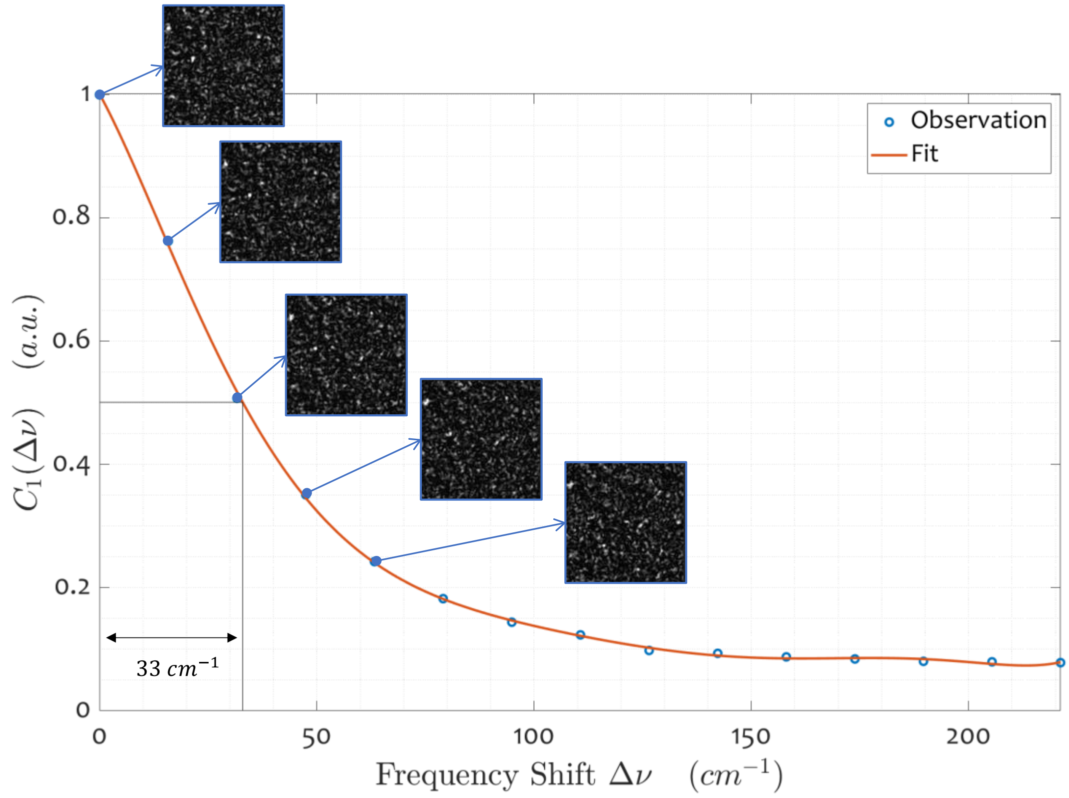

Supplementary Figure 11: Spectral Correlation function of the ground glass diffuser examined in the main manuscript

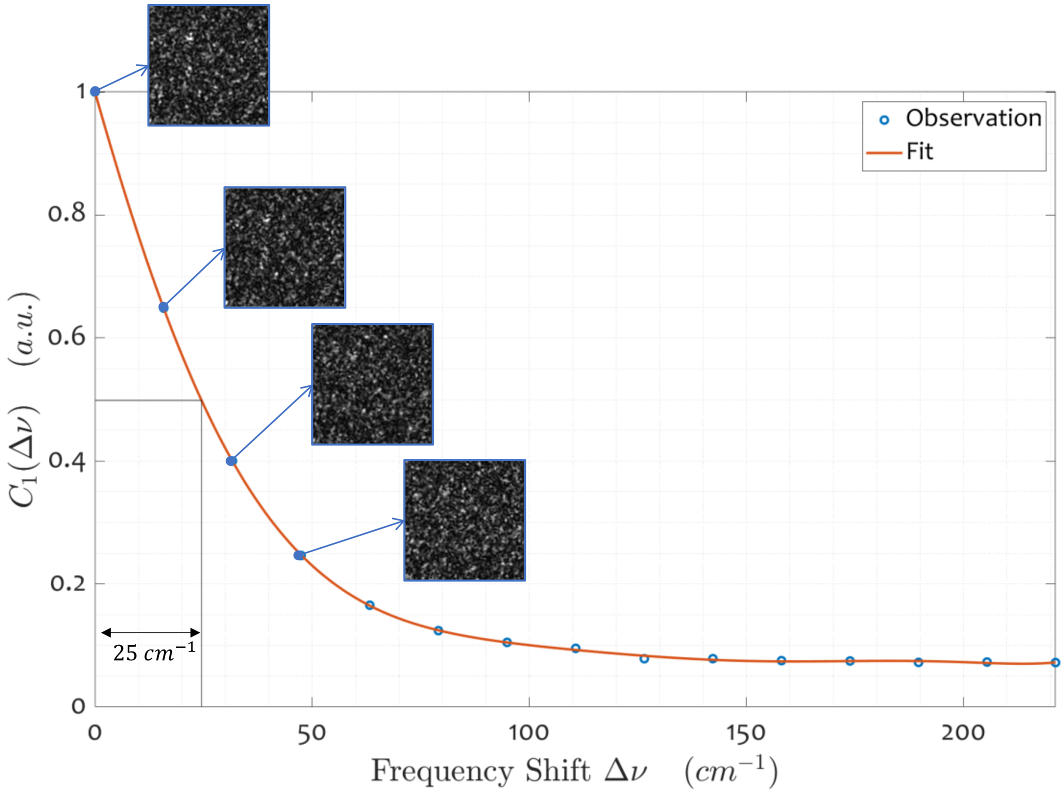

Supplementary Figure 12: Spectral Correlation function of the plastic plate examined in the main manuscript

Supplementary References

[1] Goodman JW, “Speckle phenomena in optics: theory and applications”, 2 ed., Roberts and Company Publishers, 2007.

[2] Heliotis Helicam C3 Manual (http://www.heliotis.ch/html/heliDocumentation.htm).

[3] J. W. Goodman, Introduction to Fourier Optics, McGraw-Hill, New York, NY, USA, 2ed edition, 1996.

[4] Aniceto Belmonte, "Statistical model for fading return signals in coherent lidars," Appl. Opt. 49, 6737-6748 (2010)

[5] Madabhushi, Balaji. "Indirect Imaging using Heterodyne Remote Digital Holography." MS thesis., SOUTHERN METHODIST UNIVERSITY, 2018.

[6] Liu, Y., Shen, Y., Ma, C., Shi, J., & Wang, L. V. (2016). Lock-in camera based heterodyne holography for ultrasound-modulated optical tomography inside dynamic scattering media. Applied physics letters, 108(23), 231106. doi:10.1063/1.4953630

[7] A. W. Lohmann and D. P. Paris, "Space-Variant Image Formation*," J. Opt. Soc. Am. 55, 1007-1013 (1965)

[8] Tatiana Latychevskaia, "Lateral and axial resolution criteria in incoherent and coherent optics and holography, near- and far-field regimes," Appl. Opt. 58, 3597-3603 (2019)

[9] Adolf W. Lohmann, Rainer G. Dorsch, David Mendlovic, Zeev Zalevsky, and Carlos Ferreira, "Space–bandwidth product of optical signals and systems," J. Opt. Soc. Am. A 13, 470-473 (1996)

[10] E. Arons, D. Dilworth, M. Shih, and P. C. Sun, "Use of Fourier synthesis holography to image through inhomogeneities," Opt. Lett. 18, 1852-1854 (1993)
[11] Joseph C. Marron and Kirk S. Schroeder, "Holographic laser radar," Opt. Lett. 18, 385-387 (1993)
[12] Joseph C. Marron and Kirk S. Schroeder, "Three-dimensional lensless imaging using laser frequency diversity," Appl. Opt. 31, 255-262 (1992)
[13] Mykola Kadobianskyi, Ioannis N. Papadopoulos, Thomas Chaigne, Roarke Horstmeyer, and Benjamin Judkewitz, "Scattering correlations of time-gated light," Optica 5, 389-394 (2018)
[14] F. Willomitzer, P. Rangarajan, F. Li, M. Balaji, M. Christensen, O. Cossairt, "Synthetic Wavelength Holography: An Extension of Gabor's Holographic Principle to Imaging with Scattered Wavefronts", arXiv:1912.11438 (2019)
[15] A. Viswanath, P. Rangarajan, D. MacFarlane, and M. P. Christensen, "Indirect imaging using correlography," in Computational Optical Sensing and Imaging (Optical Society of America, 2018), paper CM2E–3.
[16] Viswanath, Aparna. " Indirect Imaging Using Computational Imaging Techniques." MS thesis., SOUTHERN METHODIST UNIVERSITY, 2018.
[17] Christopher A. Metzler, Felix Heide, Prasanna Rangarajan, Muralidhar Madabhushi Balaji, Aparna Viswanath, Ashok Veeraraghavan, and Richard G. Baraniuk, "Deep-inverse correlography: towards real-time high-resolution non-line-of-sight imaging," Optica 7, 63-71 (2020)
[18] Vargas, Javier, J. Antonio Quiroga, and T. Belenguer. "Phase-shifting interferometry based on principal component analysis." Optics letters 36, no. 8 (2011): 1326-1328.
[19] A. Z. Genack, "Optical transmission in disordered media," Phys. Rev. Lett. 58, 2043–2046 (1987).
[20] G. H. Watson, S. L. McCall, P. A. Fleury, and K. B. Lyons, "Speckle autocorrelation spectroscopy and pulse transmission as probes of photon transport in strongly scattering random media", Phys. Rev. B 41, 10947 – Published 1 June 1990
[21] Mark A. Webster, Kevin J. Webb, Andrew M. Weiner, Junying Xu, and Hui Cao, "Temporal response of a random medium from speckle intensity frequency correlations," J. Opt. Soc. Am. A 20, 2057-2070 (2003)
[22] O. L. Muskens and A. Lagendijk, "Method for broadband spectroscopy of light transport through opaque scattering media," Opt. Lett. 34, 395-397 (2009)
